# New 2-aryl-6-methyl-3,4-dihydro-β-carbolin-2-iums as potential antifungal agents: Synthesis, bioactivity and structure-activity relationship

**DOI:** 10.1038/s41598-018-38222-x

**Published:** 2019-02-13

**Authors:** Xingqiang Li, Bingyu Zhang, Wei Zhao, Shanshan Yang, Xinjuan Yang, Le Zhou

**Affiliations:** College of Chemistry & Pharmacy, Northwest A&F University, Yangling, 712100 Shaanxi Province People’s Republic of China

## Abstract

Thirty new title compounds along with five known analogues were prepared from commercially available 2-arylhydrazin-1-ium chlorides and α-ketoglutaric acid. The mycelium growth rate method was used to evaluate inhibition activity against six strains of plant pathogenic fungi. Most of the compounds displayed the activity for each the fungi at 150 μΜ, higher than azoxystrobin, a positive drug. Compound 6-2 showed the lowest average IC_50_ value of 4.58 μg/mL for all the fungi where *F*. *solani* exhibited the highest susceptibility to most of the compounds. For *F*. *solani*, some compounds were more active with IC_50_ values of 2.67–8.48 μM than thiabendazole (IC_50_ = 9.30 μM) and/or carbendazim (IC_50_ = 3.36 μM). The SAR showed that the activity is significantly affected by substituents on the A-ring and/or D-ring along with the degree of unsaturation of the C-ring. Thus, a series of new β-carboline compounds with potent antifungal potential were found.

## Introduction

The most common plant disease is a variety of mycoses caused by plant pathogenic fungi, which only influence the output and quality of agricultural products but also lead to a food safety problem due to their mycotoxins harmful to animal and human health^1^. Therefore, plant mycosis is an important problem of agricultural production worldwide^2^. In current agriculture, the prevention and control of plant mycoses mainly depends on widespread application of various fungicides. However, due to the persistent and incorrect use of some commercial fungicides, drug resistance or environmental pollution has become an increasing concern^3^. Thus, it is necessary to develop new fungicides with novel molecular skeletons and environmentally friendly property. In this respect, natural product-based drug discovery has showed great potential due to the high environment compatibility of natural products in the last twenty years^4^.

**Figure 1:**
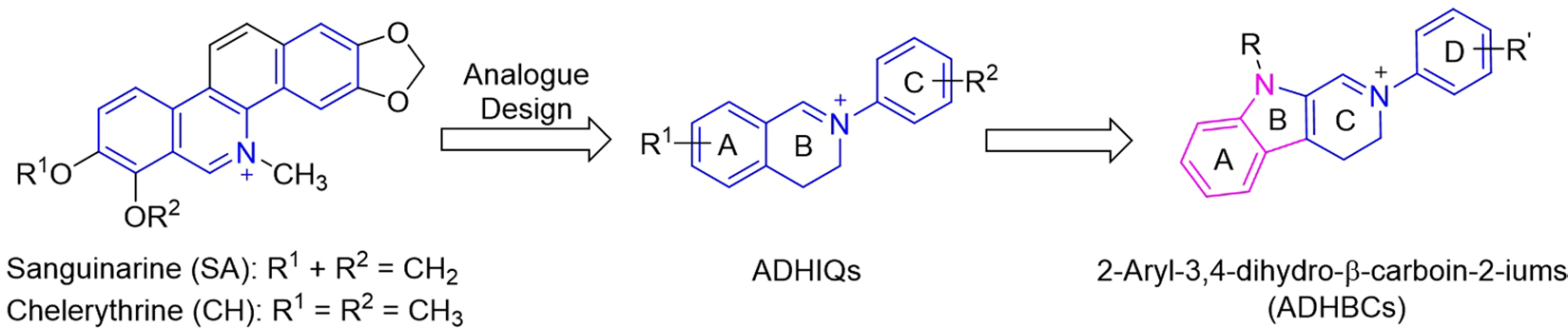
Design and Structures of 2-aryl-3,4-dihydro-*β*-carbolin-2-iums Previously Reported.

In recent years, our interests have focused on sanguinarine (SA) and chelerythrine (CH) (Fig. 1), two quaternary benzo[*c*]phenanthridine alkaloids (QBAs) with a variety of pharmacological activities, and their simple structural analogues. Our previous investigations demonstrated that the iminium moiety (C=N^+^) in SA and CH is a determinant factor for their various bioactivities^5–8^ including antifungal^5^. This result prompted us to design a class of 2-aryl-3,4-dihydroisoquinoliums (ADHIQs) as simple structural analogues of SA or CH (Fig. 1) with the aim of developing QBAs-like drugs. Compared with SA or CH, these analogues generally showed the higher anti-phytopathogenic^9–13^, anti-cancer^8,14^ and acaricidal activities^15,16^. Additionally, the analogues also showed high safety to plant growth^17,18^. Based on the results above and the antifungal activity of β-carbolines, we further designed a class of hybrids of ADHIQs and β-carbolines, i.e., 2-aryl-3,4-dihydro-β-carbolin-2-iums (ADHBCs) (Fig. 1) in order to find more potent antifungal compounds. Satisfyingly, some of the ADHBCs indeed showed the stronger activity than the corresponding ADHIQs^19^. Preliminary structure-activity relationship (SAR) showed that the substituents on the D-ring are able to significantly impact the activity of ADHBCs. As our continuing study, herein we reported a range of new ADHBCs with a structural feature of 6-methyl on the A-ring and their inhibition activity against phytopathogenic fungi. Furthermore, the SAR was also discussed. This investigation is aimed at finding more potent antifungal ADHBCs and further knowing the effect of the substituents on the A-ring the activity.

## Results and Discussion

### Design of compounds

We initially designed the primary parent compound, 9-methyl-2-phenyl-3,4-dihydro-β-carbolin-2-ium bromide (**6–34**) and its three derivatives only with one 6-methyl (**6–1**), 6-methoxy (**6–32**) or 7-fluoro (**6–33**) in order to inspect the effects of substituents on the A-ring on the antifungal activity. Meanwhile, compound **7** (Fig. 1) as the full aromatization derivative of **6–32** was also designed with the aim of inspecting the degree of unsaturation of the C-ring on the activity. Thereafter, we selected compound **6-1** with the higher activity as a secondary parent compound and designed a series of its derivatives (**6–2–6–31**) with various substituents on the D-ring to investigate the impact of the substituents of the D-ring on the activity as well as the interaction effect between substituents on the A-ring and the D-ring on the activity. The substitution patterns of target compounds are depicted in Table 1.

**Table 1:** Substitution patterns of compounds and their preliminary antifungal activity at 150 μmol/L.

Compound	Average inhibition rate ± SD (%) (*n* = 3) (around 150 μmol/L; 72 h)								Mean (%)
No.	R^1^	R^2^	CG	FOC	FOV	FON	FS	PP	
6-1	6-CH_3_	H	98.8 ± 0.9	92.8 ± 2.6	84.8 ± 2.5	94.6 ± 0.1	84.5 ± 3.3	69.1 ± 0.0	86.6
6-2	6-CH_3_	2′-F	94.6 ± 1.2	96.5 ± 0.8	98.9 ± 0.2	96.0 ± 0.0	94.1 ± 0.9	97.6 ± 0.2	96.3
6-3	6-CH_3_	3′-F	90.2 ± 0.0	83.6 ± 0.0	90.7 ± 0.8	80.8 ± 0.9	94.7 ± 0.9	94.7 ± 0.5	89.1
6-4	6-CH_3_	4′-F	100.0 ± 0.0	95.1 ± 0.7	100.0 ± 0.0	98.7 ± 0.9	96.3 ± 0.8	80.6 ± 1.6	95.1
6-5	6-CH_3_	2′-Cl	80.7 ± 0.9	48.5 ± 1.3	56.1 ± 0.5	39.1 ± 2.5	54.2 ± 2.7	74.6 ± 0.6	62.3
6-6	6-CH_3_	3′-Cl	84.9 ± 1.0	86.6 ± 0.8	81.7 ± 0.3	78.2 ± 1.5	79.6 ± 0.1	93.7 ± 0.6	85.7
6-7	6-CH_3_	4′-Cl	86.9 ± 1.1	77.4 ± 0.0	77.5 ± 0.2	83.9 ± 0.8	79.6 ± 0.1	93.2 ± 0.7	78.9
6-8	6-CH_3_	2′-Br	69.3 ± 1.1	39.4 ± 2.1	55.6 ± 0.5	23.1 ± 1.8	46.2 ± 0.9	37.6 ± 1.4	46.2
6-9	6-CH_3_	3′-Br	85.9 ± 1.1	86.0 ± 0.8	83.8 ± 1.1	76.7 ± 1.2	74.0 ± 1.1	93.1 ± 1.4	73.1
6-10	6-CH_3_	4′-Br	85.9 ± 1.1	81.6 ± 0.0	75.8 ± 1.9	81.3 ± 1.2	82.9 ± 0.0	96.8 ± 0.8	77.6
6-11	6-CH_3_	2′-I	14.6 ± 2.1	15.8 ± 1.2	<5	11.2 ± 1.6	8.2 ± 2.3	12.5 ± 3.0	<11.2
6-12	6-CH_3_	3′-I	77.1 ± 1.0	56.0 ± 0.8	74.1 ± 1.0	48.1 ± 2.5	67.4 ± 0.9	89.4 ± 0.6	66.1
6-13	6-CH_3_	4′-I	80.5 ± 1.8	67.0 ± 1.0	69.0 ± 0.0	74.8 ± 1.2	78.8 ± 1.0	86.1 ± 1.2	72.0
6-14	6-CH_3_	2′-CH_3_	87.2 ± 1.0	92.9 ± 0.9	99.5 ± 0.9	98.4 ± 1.0	99.0 ± 1.7	85.9 ± 0.6	93.8
6-15	6-CH_3_	3′-CH_3_	98.7 ± 1.2	95.0 ± 0.8	90.4 ± 1.2	94.6 ± 1.2	99.0 ± 1.7	99.1 ± 0.8	96.1
6-16	6-CH_3_	4′-CH_3_	88.2 ± 2.0	96.7 ± 0.0	86.2 ± 1.2	91.1 ± 0.2	94.6 ± 1.9	96.8 ± 0.7	92.3
6-17	6-CH_3_	2′-OMe	57.3 ± 1.4	86.4 ± 0.8	80.2 ± 0.9	90.7 ± 1.2	96.7 ± 0.0	81.7 ± 0.7	78.3
6-18	6-CH_3_	3′-OMe	100.0 ± 0.0	83.1 ± 0.0	83.1 ± 0.0	92.2 ± 1.0	93.5 ± 1.0	81.7 ± 0.7	87.5
6-19	6-CH_3_	4′-OMe	86.0 ± 0.0	74.1 ± 0.9	67.8 ± 1.0	91.2 ± 1.2	91.1 ± 1.0	89.5 ± 0.7	81.8
6-20	6-CH_3_	2′-OH	66.7 ± 1.0	83.5 ± 0.8	72.9 ± 0.9	90.7 ± 1.2	95.6 ± 1.0	80.5 ± 0.7	80.9
6-21	6-CH_3_	3′-OH	77.8 ± 0.0	73.9 ± 1.0	79.4 ± 1.0	87.0 ± 2.2	95.2 ± 1.0	76.6 ± 1.2	81.7
6-22	6-CH_3_	4′-OH	71.7 ± 0.0	76.1 ± 1.0	51.4 ± 1.3	91.6 ± 1.1	92.4 ± 1.5	82.3 ± 1.0	80.8
6-23	6-CH_3_	3′-CF_3_	73.6 ± 1.0	59.2 ± 1.2	57.0 ± 0.5	71.3 ± 0.8	60.9 ± 1.0	68.9 ± 0.0	65.4
6-24	6-CH_3_	4′-CF_3_	71.3 ± 1.1	32.0 ± 1.2	36.0 ± 1.9	70.5 ± 0.0	38.7 ± 0.4	49.2 ± 1.0	48.4
6-25	6-CH_3_	3′-NO_2_	<5	8.2 ± 0.8	9.2 ± 2.5	<5	8.8 ± 2.8	<5	<6.9
6-26	6-CH_3_	3′- Ac	92.0 ± 1.1	76.5 ± 0.8	74.0 ± 0.9	85.6 ± 0.8	88.4 ± 0.0	28.4 ± 5.0	71.3
6-27	6-CH_3_	2′,6′-2F	78.2 ± 0.0	57.3 ± 1.2	91.7 ± 0.9	58.7 ± 0.4	75.2 ± 0.8	90.6 ± 0.6	73.5
6-28	6-CH_3_	2′,4′-2Cl	23.4 ± 1.7	<5	<5	<5	15.0 ± 3.0	<5	<9.7
6-29	6-CH_3_	3′,5′-2Cl	<5	<5	<5	<5	<5	<5	<5.0
6-30	6-CH_3_	2′,4′-2Br	17.5 ± 1.8	<5	<5	<5	9.3 ± 8.7	<5	<7.8
6-31	6-CH_3_	2′-F-4′-Br	<5	<5	<5	<5	<5	<5	<5.0
6-32	7-F	H	99.6 ± 0.7	70.6 ± 5.9	58.7 ± 1.5	97.2 ± 0.1	82.4 ± 2.6	98.4 ± 1.1	83.0
6-33	6-OMe	H	73.9 ± 1.2	74.1 ± 0.8	49.4 ± 1.9	91.1 ± 1.4	82.4 ± 1.6	89.5 ± 1.1	76.7
6-34	H	H	94.7 ± 2.3	90.3 ± 3.3	64.6 ± 1.4	56.4 ± 5.8	88.5 ± 1.0	87.4 ± 0.9	78.9
7	7-F	H	18.0 ± 3.5	34.6 ± 3.2	25.1 ± 3.1	16.2 ± 0.6	24.5 ± 1.6	12.5 ± 3.7	21.6
Azoxystrobin			53.8 ± 0.0	73.3 ± 0.0	77.8 ± 1.9	56.0 ± 0.0	65.7 ± 0.0	60.2 ± 0.7	64.5
Thiabendazole			96.2 ± 0.0	100.0 ± 0.0	100.0 ± 0.0	100.0 ± 0.0	100.0 ± 0.0	97.9 ± 1.8	99.0
Carbendazim			100.0 ± 0.0	100.0 ± 0.0	100.0 ± 0.0	100.0 ± 0.0	100.0 ± 0.0	100.0 ± 0.0	100.0

^a^CG: *C. gloeosporioides*; FOC: F. oxysporum f. sp. cucumerinum; FOV: F. oxysporum f. sp. vasinfectum; FON: F. oxysporum f. sp. cucumerinum; FOV: F. oxysporum f. sp. vasinfectum; FON: F. oxysporum sp.

niveum; FS: F. solani; PP: P. piricola.

### Chemistry

**Figure 2:**
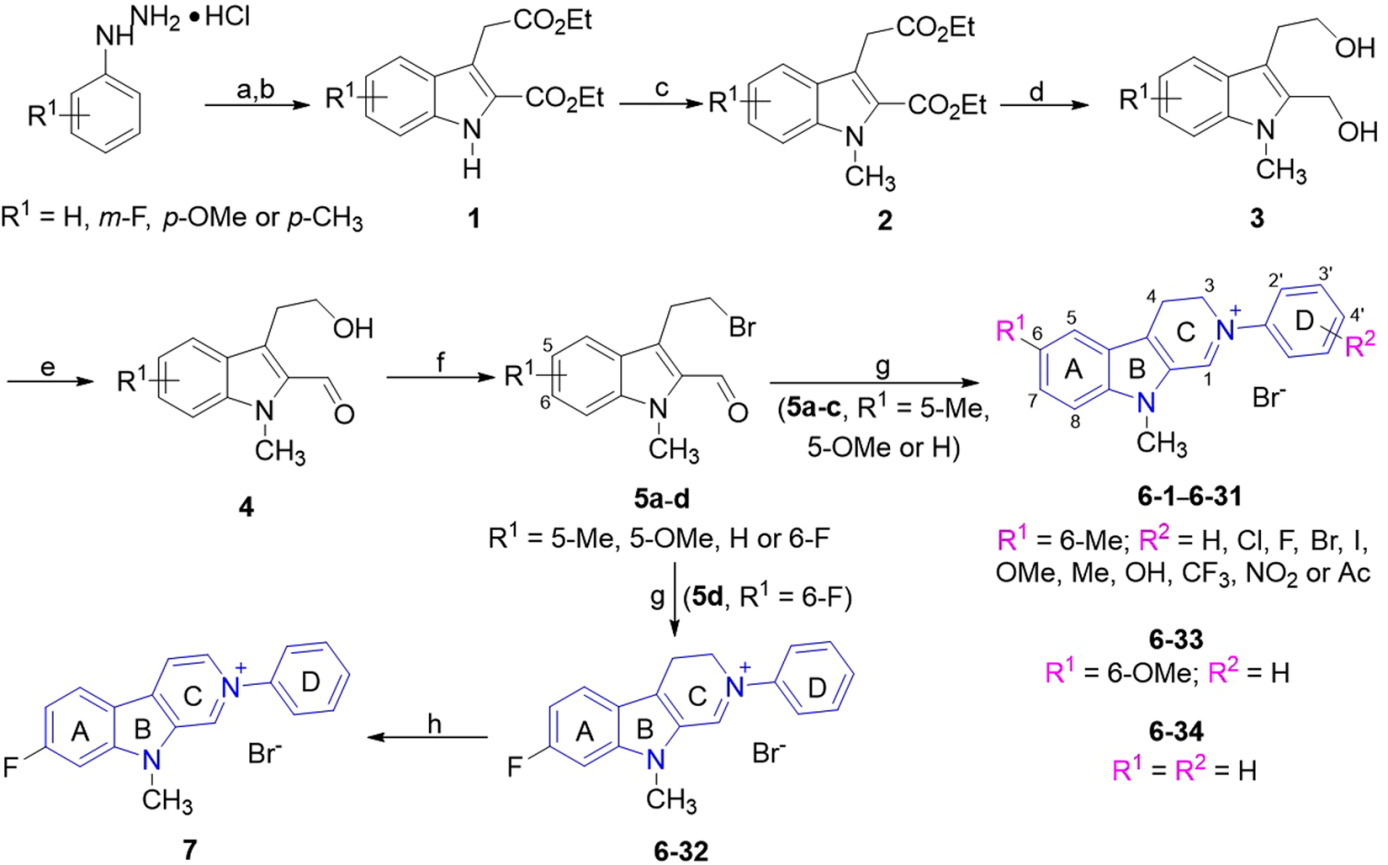
Synthesis of target compounds 6-1−6-34 and 7. Reagents and conditions: (**a**) *α*-ketoglutaric acid, H_2_O, 24 h at r.t.; (**b**) EtOH, con. H_2_SO_4_, reflux for 12 h; (**c**) CH_3_I, NaH, dry DMF, 0 °C; (**d**) LiAlH_4_, dry THF, 0 °C to r.t.; (**e**) active MnO_2_, CHCl_3_, r.t.; (**f**) MsCl, Et_3_N, LiBr, dry THF, 0 °C to r.t.; (**g**) R^2^-PhNH_2_, TsOH·H_2_O, EtOH, r.t.; (**h**) Pd/C, CH_3_CN, 80 °C.

Compounds **6-*n*** (*n* = 1–34) and **7** were synthesized according to our previous method^19^ outlined in Fig. 2. The key intermediates **5a**–**5d** were synthesized *via* 6 steps from commercially available 2-arylhydrazin-1-ium chlorides and α-ketoglutaric acid^20^. Target compounds **6-*n*** were obtained by reaction of **5a**–**5d** and primary aromatic amines in ethanol by using *p*-toluenesulfonic acid as catalyst in 35‒99% yields. Generally, anilines with electron-donating groups such as methyl, methoxy or hydroxyl gave the higher yield while electron-withdrawing groups such as nitro, trifluoromethyl or acetyl led to the lower yield. Unexpectedly, the method above did not afford some of the desired **6** with 2′-CF_3_, 2′- or 4′-NO_2_ or 2′- or 4′-acetyl, due to the lower reactivity of the corresponding aromatic amines. Compound **7** was obtained by treatment of **6–32** with Pd/C in 93% yield.

Except **6–34**^19,20^ and **7**^21^, all the target compounds are new compounds and their structures were structurally characterized by spectrometric methods including ^1^H NMR, ^13^C NMR, and MS. All compounds **6** displayed some similar spectroscopic features because of the same molecular skeleton. In ^1^H spectra, all the compounds revealed one singlet signals of H-1 in the range of *δ*_H_ 9.38 to 9.72 ppm, one singlet signal of (N)CH_3_ at *δ*_H_ ca. 3.97 (3H, s) and two triplet signals of one CH_2_CH_2_ moiety at *δ*_H_ ca. 3.5 (2 H, t, *J* = ca. 8.7 Hz) and ca. 4.5 (2 H, t, *J* = ca. 8.7 Hz); In ^13^C NMR, each the compound showed the signals of C-1 in the range of *δ*_C_ 153.2 to 163.6 ppm, (N)CH_3_ at *δ*_C_ ca. 30.9 and one CH_2_CH_2_ moiety at *δ*_C_ ca. 52.0 (C-3) and ca. 20.4 (C-4); Additionally, compounds **6-1**‒**6-31** also gave signals of 6-Me at *δ*_H_ ca. 2.43 (3H, s) and *δ*_C_ ca. 21.5. In positive HR-MS spectra, each compounds **6-*n*** showed a characteristic ion peak at *m*/*z* [M–Br]^+^. The presence of bromide ion had been confirmed by negative ESI-MS in our previous similar study^11,19,21^. Due to the similarity of the synthetic method and structures, we did not assay the bromide ion in the present compounds.

### Bioactivity

The antifungal activity *in vitro* of compounds was assayed according to the mycelium linear growth rate method^11^. Plant pathogenic fungi *Colletotrichum gloeosporioides*, *Fusarium oxysporum* f. sp. *cucumerinum*, *Fusarium oxysporum* f. sp. *vasinfectum*, *Fusarium oxysporum* sp. *niveum*, *Fusarium*.*solani* and *Physalospora piricola* were used as test fungi. The preliminary activities of compounds **6-*n*** and **7** were determined at 150 μM (ca. 50 μg/mL). Commercial fungicide standards thiabendazole (TBZ), azoxystrobin (ASB) and carbendazim (CBZ) were used as the reference. The results are shown in Table 1.

Satisfyingly, most of the compounds revealed the good to excellent activity with average inhibition rates of 72‒96% against all the fungi, superior to that of ASB (64.5%). According to the average inhibition rates, compounds **6-*n*** may be divided into four groups. The first group consisting of **6-2**, **6-4** and **6-14**–**6-16** showed the highest activity with average inhibition rates of 92–96%. The 2^nd^ group including 18 compounds (**6-1**, **6-3**, **6-6**, **6–7**, **6-9**, **6–10**, **6–13**, **6–17–6–22**, **6–26**, **6–27**, **6–32** and **6–33**) showed the higher activity (71–89%). The 3^rd^ group containing 5 compounds (**6–5**, **6–8**, **6–12**, **6–23** and **6–24**) displayed the moderate activity (46–66%). The last group consisting of the other (**6–11**, **6–25** and **6-28**–**6-31**) gave very low or less activity (≤11%). For compounds **6**, almost half of all the test items (100/204) revealed >80% of inhibition rate.

In order to explore the antifungal potential and SAR, the more active compounds in Table 1 were subjected to determination of median inhibition concentrations (IC_50_). Thiabendazole (TBZ) and carbendazim (CBZ) were used as positive controls. Compound **6–34** (R^1^ = R^2^ = H) were used as a reference control. The results are shown in Table 2.

**Table 2:** Median effective concentrations (IC_50_) of the compounds.

No.	IC_50_ [μmol/L (μg/mL)]^a^						Mean (μM)
	CG	FOC	FOV	FON	FS	PP	
6-1	39.4 n (14.0)	24.0 f (8.53)	22.1 ef (7.84)	31.8 i (11.3)	6.08 f (2.16)	50.4 p (17.9)	29.0
6-2	14.8 b (5.51)	11.7 b (4.37)	10.3 c (3.86)	19.2 d (7.15)	7.18 i (2.68)	10.4 c (3.90)	12.3
6-3	16.4 d (6.12)	28.9 gh (10.8)	30.6 i (11.0)	52.8 n (19.7)	6.43 g (2.40)	14.8 g (5.51)	25.0
6-4	30.8 k (11.5)	21.2 e (7.90)	21.3 e (7.95)	33.2 j (12.4)	5.44 e (2.03)	36.7 l (13.7)	24.8
6-5	18.7 f (7.29)	≈150^*^	100–150^*^	>150^*^	100–150^*^	24.5 i (9.55)	—
6-6	18.6 f (7.22)	22.4 e (8.74)	30.0 i (11.7)	28.0 g (10.9)	6.65 h (2.59)	12.4 d (4.82)	19.7
6-7	18.9 f (7.35)	37.5 jk (14.6)	40.0 l (15.6)	26.4 f (10.3)	2.68 a (1.05)	14.3 f g (5.58)	23.3
6-8	31.2 k (13.5)	>150^*^	100–150^*^	>150^*^	>150^*^	>150^*^	—
6-9	15.6 c (6.76)	21.3 e (9.25)	27.3 h (11.9)	33.1 j (14.3)	20.1 q (8.74)	10.9 c (4.71)	21.4
6-10	16.8 d (7.31)	29.8 h (13.0)	48.9 o (21.2)	33.2 j (14.4)	8.48 j (3.68)	10.1 c (4.37)	24.5
6-11	>150^*^	>150^*^	>150^*^	>150^*^	>150^*^	>150^*^	—
6-12	17.9 e (8.60)	100–150^*^	35.5 j (17.1)	≈150^*^	21.7 r (10.4)	14.4 g (6.91)	—
6-13	18.4 f (8.84)	46.7 l (22.4)	46.0 n (22.1)	34.5 k (16.6)	6.68 h (3.22)	12.9 de (6.19)	27.5
6-14	41.1 n (14.9)	15.9 d (5.87)	24.5 g (9.09)	30.1 h (11.1)	3.47 b (1.43)	41.4 n (15.0)	26.1
6-15	24.2 hi (6.27)	13.3 c (4.49)	28.4 h (9.53)	52.9 n (12.7)	2.67 a (0.99)	21.2 h (6.26)	23.8
6-16	35.6 m (14.5)	33.9 i (9.90)	22.6 f (12.8)	36.0 l (13.3)	4.00 c (1.48)	21.5 h (9.90)	25.6
6-17	100–150^*^	25.0 f (9.61)	22.8 f (8.78)	17.4 c (6.70)	9.95 l (3.83)	23.6 i (9.09)	—
6-18	32.4 l (12.4)	36.5 j (14.1)	37.6 k (14.5)	17.3 c (6.65)	13.7 mn (5.26)	26.0 j (10.0)	27.2
6-19	48.8 o (18.8)	47.5 l (18.3)	64.4 s (24.8)	23.7 e (9.15)	14.9 no (5.74)	21.8 h (8.40)	36.9
6-20	49.3 o (18.3)	38.2 k (14.2)	58.7 r (21.8)	26.6 f (9.88)	17.9 p (6.65)	62.8 q (23.3)	42.3
6-21	59.3 p (22.0)	64.1 n (23.8)	57.1 q (21.2)	44.4 m (16.5)	14.6 n (5.43)	29.6 k (11.0)	44.9
6-22	65.7 q (24.4)	57.1 m (21.2)	≈150^*^	25.4 ef (9.42)	18.3 p (6.80)	38.5 m (14.3)	—
6-23	15.3 c (6.49)	100–150^*^	100–150^*^	69.0 o (29.2)	15.5 o (6.55)	64.0 q (27.1)	—
6-24	27.8 j (11.8)	>150^*^	>150^*^	>150^*^	13.0 m (5.50)	≈150^*^	—
6-25	≫150^*^	≫150^*^	≫150^*^	≫150^*^	≫150^*^	≫150^*^	—
6-26	22.3 h (8.87)	36.5 j (14.5)	43.3 m (17.2)	19.7 d (7.84)	15.2 o (6.03)	>150^*^	—
6-27	24.3 i (9.50)	100–150^*^	16.0 d (6.27)	100–150^*^	18.4 p (7.19)	13.4 ef (5.23)	—
6-28	>150^*^	≫150^*^	≫150^*^	≫150^*^	≫150^*^	≫150^*^	—
6-29	≫150^*^	≫150^*^	≫150^*^	≫150^*^	≫150^*^	≫150^*^	—
6-30	>150^*^	≫150^*^	≫150^*^	≫150^*^	≫150^*^	≫150^*^	—
6-31	≫150^*^	≫150^*^	≫150^*^	≫150^*^	≫150^*^	≫150^*^	—
6-34	20.3 g (6.89)	27.7 g (9.41)	50.6 p (17.2)	44.7 m (15.2)	4.40 d (1.51)	48.3 o (16.4)	32.7
TBZ^b^	2.61 b (0.53)	12.7 bc (3.04)	7.78 b (1.31)	8.25 b (1.66)	9.30 k (2.52)	5.44 b (1.09)	7.68
CBD^c^	0.64 a (0.12)	4.14 a (0.79)	5.55 a (1.06)	3.56 a (0.68)	3.36 b (0.64)	0.50 a (0.10)	2.96

^a^The significant difference exists among the data without the same lowercase letters within a column (*P* < 0.05). CG: *C*. *gloeosporioides*; FOC: *F*. *oxysporum* f. sp. *cucumerinum*; FOV: *F*. *oxysporum* f. sp. *vasinfectum*; FON: *F*. *oxysporum* sp. *niveum*; FS: *F*.*solani*; PP: *P*. *piricola*. ^b^Thiabendazole. ^c^Carbendazim. ^*^Estimated values based on the results in Table 1.

Seventeen compounds showed average IC_50_ values of 12.3–45 μM against all the fungi. Among them, compounds **6–2** and **6-6** gave average IC_50_ values of <20 μM, eleven compounds (**6-1**, **6-3**, **6-4**, **6-7**, **6-9**, **6-10**, **6-13**−**6-16**, **6-18**) 20–29 μM and four compounds (**6-19**–**6-21**, **6-34**) 32–45 μM. Additionally, six compounds (**6-12**, **6-17**, **6-22**, **6-23**, **6-26**, **6-27**) exhibited the higher activity with average IC_50_ values of 18−41 μM against four or five strains out of the fungi. Among all the compounds, **6-2** showed the lowest average IC_50_ value of 12.3 μM and the highest activity against *F*. *oxysporum* f. sp. *cucumerinum* (IC_50_ = 11.7 μM), *C*. *gloeosporioides* (IC_50_ = 14.8 μM), *F*. *oxysporum* f. sp. *vasinfectum* (IC_50_ = 10.3 μM) and *P*. *piricola* (IC_50_ = 10.4 μM), while **6-6** and **6-9** gave the second highest average activity (IC_50_ = 19.7, 21.4 μM). **6-17** and **6-18** were most active against *F*. *oxysporum* sp. *niveum* (IC_50_ = 17.4, 17.3 μM) followed by **6-2** (IC_50_ = 19.2 μM) while **6-7** and **6-15** were most active against *F*. *solani* (IC_50_ = 2.68, 2.67 μM). Among all the test fungi, *F*. *solani* showed the highest susceptibility to most of the compounds, followed by *P*. *piricola*. For *F*. *solani*, 12 compounds (IC_50_ = 2.7−8.5 μM) were more active than TBZ (IC_50_ = 9.30 μM) and two compounds (**6-7**, **6-15**) (IC_50_ ≈ 2.7 μM) were superior to CBZ (IC_50_ = 3.36 μM). For *F*. *oxysporum* f. sp. *cucumerinum*, the activity of **6-2** (IC_50_ = 11.7 μM) was comparable with TBZ (IC_50_ = 12.7 μM).

## Discussions

### Structure-activity relationship

**Figure 3:**
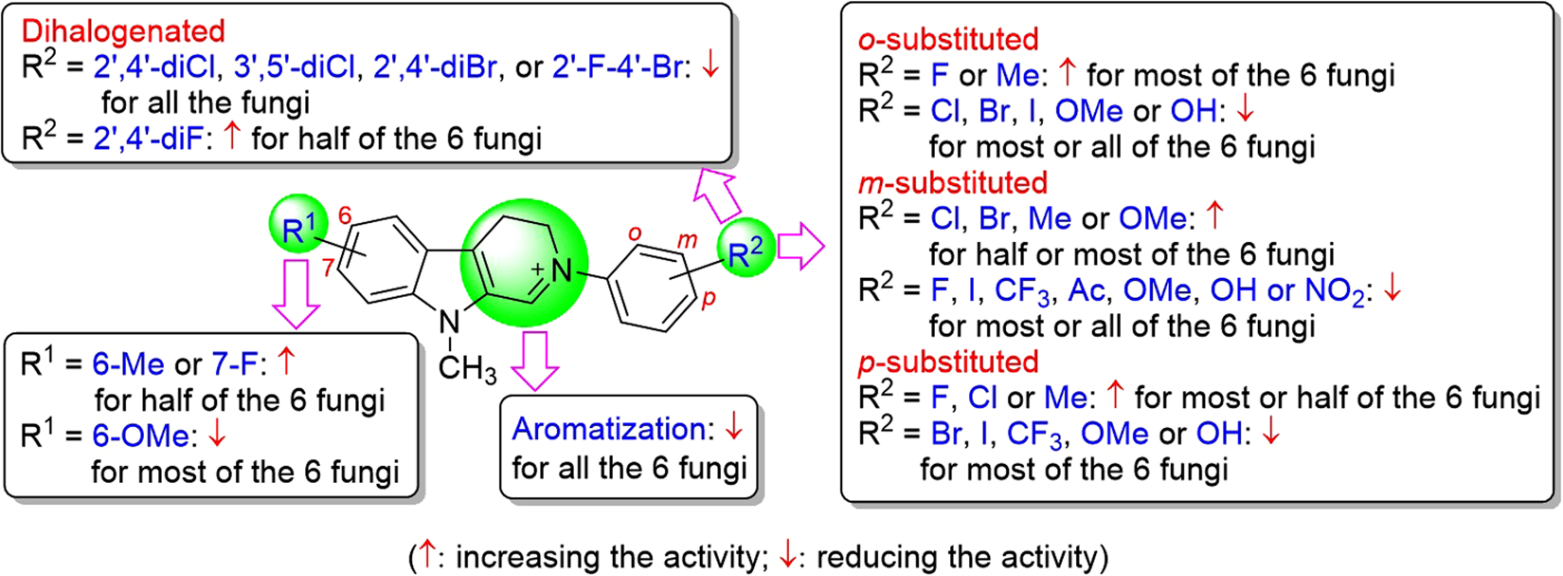
Structure-activity relationship of compounds 6-*n*.

Based on the results in Tables 1 and 2, we found that the presence of substituents on the A-ring and/or D-ring can significantly impact the activity (Fig. 3). But the influence varies with both substitution patterns of the substituents and the species of the fungi. For the A-ring, the presence of 6–Me can increase the activity against *F*. *oxysporum* f. sp. *cucumerinum*, *F*. *oxysporum* f. sp. *vasinfectum* and *F*. *oxysporum* sp. *niveum* but decrease the activity against the other fungi (**6-1** *vs* **6-34**) (*P* < 0.05) (Table 2). However, 6-OMe only significantly increases the activity against *F*. *oxysporum* sp. *niveum* (**6-1** *vs* **6-33**) (Table 1). By contrast, 7-F enhances the activity against *C*. *gloeosporioides*, *F*. *oxysporum* sp. *niveum* and *P*. *piricola* but reduces the activity against the other fungi (**6-1** *vs* **6-32**) (Table 1). Interestingly, 6-Me, 6-OMe and 7-F improve the activity against *F*. *oxysporum* sp. *niveum* (Table 1).

A similar case was also observed for substituents on the D-ring. For the halogenated compounds, compared with **6-1** (R^2^ = H), 2′-F (**6-2**), 4′-F (**6-4**), 3′-Cl (**6-6**), 4′-Cl (**6-7**) and 3′-Br (**6-9**) dramatically improve the activity against most or half of the fungi while 3′-F (**6-3**), 2′-Cl (**6-5**), 2′-Br (**6-8**), 4′-Br (**6-10**), 2′-I (**6-11**), 3′-I (**6-12**) and 4′-I (**6-13**) reduce the activity against most or all of the fungi. It is worth noting that all the mono-halogenated compounds except **6-8** (2′-Br) and **6-11** (2′-I) show enhancement effect of the activity against both *C*. *gloeosporioides* and *P*. *piricola* (**6-2**−**6-7**, **6-9**, **6-12**, **6-13** vs **6-1**). By contrast, only the minority of the mono-halogenated compounds display improvement of the activity on the other fungi (**6-2**−**6-13** vs **6-1**). Unexpectedly, dihalogenation of the D-ring causes dramatic decrease or loss of the activity in all the cases (**6-28**–**6-31** *vs* **6-1**) except 2′,6′-diF (**6-27**) increasing the activity against part of the fungi.

In addition, 2′-, 3′- or 4′-Me can also improve the activity against half or most of the fungi (**6-14**−**6-16** vs **6-1**), where three methylated compounds increase the activity on both *F*. *solani* and *P*. *piricola*. In contrast to methyl (a weak electron-donating group) or halogen atoms (a weak electron-withdrawing group), OH, OMe, CF_3_, NO_2_ or acetyl as strong electron-donating or electron-withdrawing groups decreases the activity against most or all of the fungi (**6-17**–**6-26** *vs* **6-1**). Interestingly, three methoxy-substituted isomers (**6-17**–**6-19**) and two out of three hydroxylation isomers (**6-20**–**6-22**) show improvement of the activity against both *F*. *oxysporum* sp. *niveum* and *P*. *piricola* compared with **6-1**.

The above SARs are similar but not the same as that of 2-aryl-3,4-dihydro-β-carbin-2-niums without substituents on the A-ring^19^, where the activity order of the three methylated compounds against *F*. *solani* is 2′-Me isomer ≈4′-Me isomer >3′-Me isomer. However, for the present compounds with 6-Me, three methylated compounds show the different activity order for *F*. *solani*: 3′-Me isomer (**6-15**) > 2′-Me isomer (**6-14**) > 4′-Me isomer (**6-16**). A similar trend of the activity was also found for hydroxyl-substituted isomers (**6-20**−**6-22**). Undoubtedly, the difference is caused by the introduction of 6-methyl to the A-ring, suggesting that substituents on the A-ring influence the effect of substituents of the D-ring on the activity. In other word, there exists an interaction effect between substituents of both the A-ring and the D-ring on the activity.

Comparison of the activity of the 3,4-dihydro compound **6-32** and its full aromatic derivative **7** found that the C-ring aromatization leads to dramatic decrease of the activity. This case is agreement with that of 2-aryl-3,4-dihydro-β-carbon-2-niums without substituents on the A-ring^19^. This result in combination with the aforementioned substituent effect again suggests that the activity of the compounds is affected by the electron density distribution on its molecular framwork, especially on the C=N^+^ bond as a structural determinant of the bioactivities including antimicrobial, acaricidal, anticancer and anti-acetylcholinesterase activity^6–16,19,21^.

**Figure 4:**
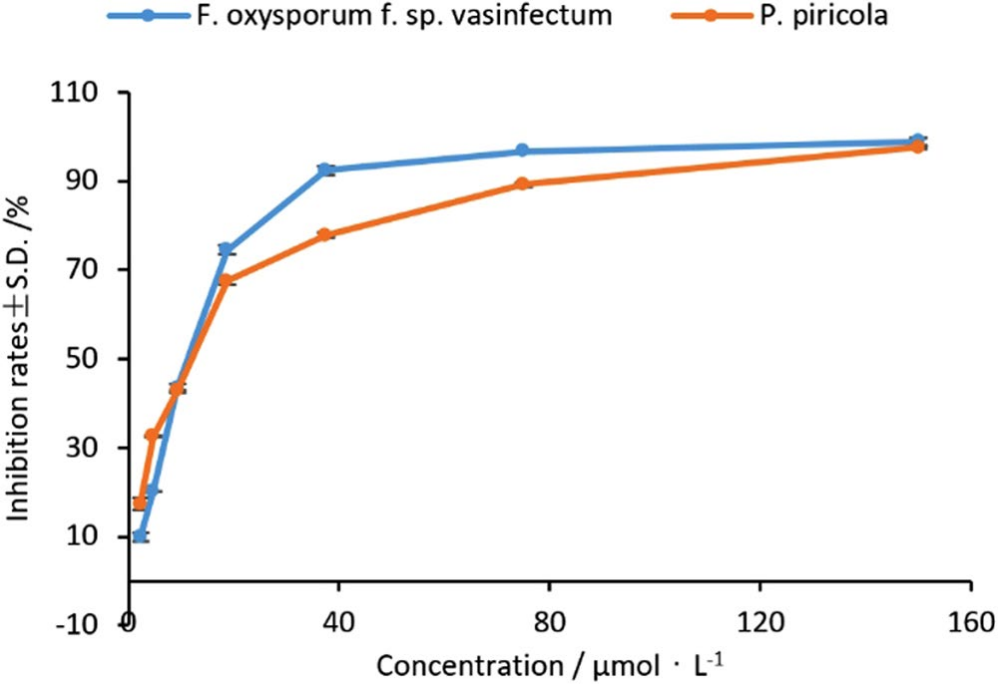
The concentration-effect curves of compound 6-2 against *F*. *oxysporum* f. sp. *vasinfectum* and *P*. *piricola*.

**Figure 5:**
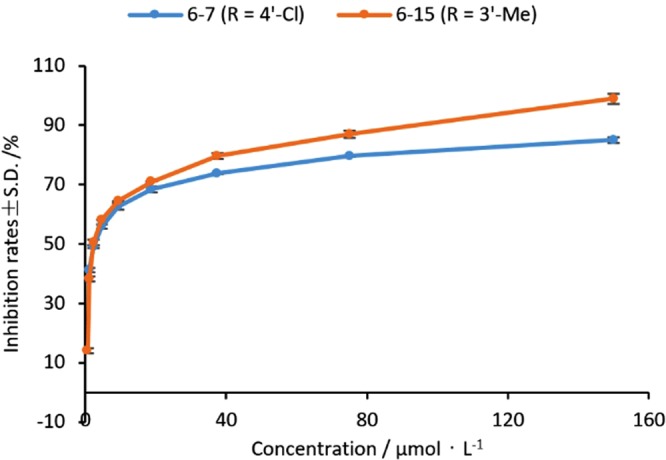
The concentration-effect curves of compounds 6-7 and 6-15 against F. solani.

Interestingly, **6-2** gave almost the same EC_50_ values (≈10.4 μM) for *F*. *oxysporum* f. sp. *vasinfectum* and *P*. *piricola*, but showed two obvious different concentration-effect curves for the two fungus species (Fig. 4). Similarly, compounds **6-7** and **6-15** having almost the same EC_50_ values (≈2.7 μM) for *F*. *solani* also showed the different concentration-effect curves (Fig. 5). The trend of two curves in Fig. 4 indicates that **6-2** is obviously more active against *F*. *oxysporum* f. sp. *vasinfectum* than *P*. *piricola* while Fig. 5 shows that **6-15** is more active than **6-7** against *F*. *solani*. The results above suggest that there are very likely more than one bio-target in the same fungal cell for this class of compounds. The speculation above may be supported to some extent by the fact that SA or CH, as the model compound of **6-n**, can affect the activities of various enzymes in cells^22^. For the fungus having multi-acceptors of the compounds, an IC_50_ value actually reflects a combined consequence of action of the compound and all the corresponding acceptors in the fungal cell. Although the same class of compounds may have different degree of biological effect for one specific bio-target in the same fungal cell, it is still possible for these compounds to have the same or similar overall biologic effect such as IC_50_ values for the fungus containing multi-acceptors. Obviously, for the fungi having multiple drug targets, IC_50_ values don’t fully or exactly reflect the difference of the activity of the different drug. However, to date, no representation methods of activity suitable for the situation above has been reported. In our opinion, using [IC_50_ × (IC_90_ − IC_50_)]^1/2^ instead of IC_50_ should be more reasonable to represent the strength of activity.

Compared with the isoquinoline analogues (ADHIQs) (Fig. 1) with the same 2-aryl, the present compounds are more active in most cases. For example, for *F*. *oxysporum* f. sp. *vasinfectum*, compound **6-2** (R^2^ = 2′-F) with an IC_50_ value of 10.3 μM was more active than the corresponding 2′-F-subtituted isoquinoline compound (R^1^ = H) (IC_50_ = 72.2 μM)^12^. A similar case was also observed in our previous study on antifungal activity of the other 9-methyl-3,4-dihydro-β-carboline compounds without substituents on the A-ring (Fig. 1, ADHBCs)^19^. The results above further indicate that 2-phenyl-3,4-dihydro-β-carboline is a more promising lead structure than 2-phenyl-3,4-dihydroisoquinoline for development of new antifungal agents.

In conclusion, we synthesized a range of new 2-aryl-3,4-dihydro-*β*-carboline bromides and evaluated for their antifungal activity *in vitro* against phytopathogenic fungi. The most of the compounds were found to be more active than azoxystrobin, a positive fungicide, in most cases. Among all the fungi, *F*. *solani* showed the highest susceptibility to most of compounds and some of the compounds were more active than positive drugs thiabendazole and/or carbendazim. Compound **6-2** showed the greatest potential with IC_50_ values of 7.18−19.2 μM for development of new antifungal agents. SAR analysis showed that substituents on both the A-ring and that the D-ring significantly impact the activity in an independent or combined manner, and the activity should be closely related with the electron density distribution of the molecular skeleton. Therefore, it is necessary to explore the *in vivo* activity of the compounds and conduct more extensive structure optimization.

## Methods

### Chemicals

Azoxystrobin (ASB, $\geqslant$98%), thiabendazole (TBZ, $\geqslant$99%) and carbendazim (CBZ, $\geqslant$99%), three commercial fungicide standards, were purchased from Sigma-Aldrich Trading Co. Ltd. (Shanghai, China). *p*-Tolyhydrazine hydrochloride, *p*-methoxyphenylhydrazine hydrochloride, *p*-fluorophenylhydrazine hydrochloride and 2-oxopentanedioic acid were bought from Aladdin Industrial Inc. (Shanghai, China). Other reagents and solvents were obtained locally and of analytical grade. The water used was ion-free.

### Instruments

Melting points of the compounds were determined on an XT-4 micro-melting point apparatus (Beijing Tech Instrument Co., Ltd. China) and uncorrected. Nuclear magnetic resonance (NMR) spectra were recorded on a Bruker Avance III instrument (^1^H, 500 MHz; ^13^C, 125 MHz). Chemical shift values (δ) and coupling constant values (J) were given in parts per million (ppm) and Hz, respectively. Mass spectroscopy were performed using a micrOTOF-Q II instrument (Bruker, Karlsruhe, Germany).

### Synthesis

#### General procedure for the synthesis of compounds 6-1−6-34

According to our previous method^19^, intermediates **5a** (R^1^ = 5-Me), **5b** (R^1^ = 5-OMe), **5c** (R^1^ = H) and **5d** (R^1^ = 5-F) were prepared *via* 6 steps starting from 2-oxopentanedioic acid and *p*-tolyhydrazine hydrochloride, *p*-methoxyphenylhydrazine hydrochloride, phenylhydrazine hydrochloride and *p*-fluorophenylhydrazine hydrochloride, respectively. Compound **5a**, **5b**, **5c** or **5d** (200 mg) reacted with aniline or substituted anilines in ethanol (20 mL) containing *p*-toluenesulfonic acid monohydrate (7.6 mg) at room temperature under stirring for 4 h to 3 d. After the end of reaction, the solvent was removed under reduced pressure. The resulting residue was suspended in ethyl acetate. After intensely stirring for about 10 min at room temperature, the solids were collected by filtration and repeatedly washed with ethyl acetate to provide the desired compounds **6**. The 1 H and/or 13C NMR data of the compounds above were showed in Supporting Information and consistent with those in literature.

#### 6,9-Dimethyl-2-phenyl-3,4-dihydro-β-carbolin-2-ium bromide (**6-1**)

Yield, 71%; orange powders; mp 235.2–236.2 °C; ^1^H NMR (500 MHz, DMSO-d_6_): δ 9.50 (s, 1H), 7.87 (d, J* = *7.7 Hz, 2H), 7.67 (t, J = 7.8 Hz, 2H), 7.63-7.60 (m, 3H), 7.44 (d, J = 8.8 Hz, 1H), 4.60 (t, J = 8.7 Hz, 2H), 3.97 (s, 3H, CH_3_), 3.50 (t, J = 8.7 Hz, 2H), 2.43 (s, 3H, CH_3_); ^13^C NMR (126 MHz, DMSO-*d*_6_): δ 153.8, 143.9, 142.4, 132.9, 131.7, 130.3, 130.3, 129.1, 128.5, 126.0, 124.5, 123.9, 123.2, 121.7, 112.2, 51.9, 30.9, 21.5, 20.3; HR-ESI-MS (*m*/*z*): [M–Br]^+^ calcd. for C_19_H_19_N_2_^+^, 275.1543; found, 275.1538.

#### 6,9-Dimethyl-2-(2-fluorophenyl)-3,4-dihydro-β-carbolin-2-ium bromide (**6-2**)

Yield, 54%; orange powders; mp 327.5–328.6 °C; ^1^H NMR (500 MHz, DMSO-d_6_): δ 9.57 (s, 1H), 7.98 (t, J = 7.5 Hz, 1H), 7.69–7.59 (m, 4 H), 7.50 (t, J = 7.5 Hz, 1H), 7.47 (d, J = 8.7 Hz, 1H), 4.49 (t, J = 8.4 Hz, 2H), 3.96 (s, 3H, CH_3_), 3.50 (t, J = 8.4 Hz, 2H), 2.43 (s, 3H, CH_3_); ^13^C NMR (126 MHz, DMSO-*d*_6_): δ 156.6, 155.3 (d, J = 251.5 Hz), 142.9, 133.6, 132.7 (d, J = 8.6 Hz), 132.0, 131.5 (d, J = 10.3 Hz), 129.0, 127.5, 126.2, 125.7, 124.1, 121.9, 117.7 (d, J = 19.1 Hz), 112.3, 52.8, 31.0, 21.4, 20.4; HR-ESI-MS (*m*/*z*): [M–Br]^+^ calcd. for C_19_H_18_FN_2_^+^, 293.1449; found, 293.1445.

#### 6,9-Dimethyl-2-(3-fluorophenyl)-3,4-dihydro-β-carbolin-2-ium bromide (**6-3**)

Yield, 62%; orange powders; mp 268.3–269.5 °C; ^1^H NMR (500 MHz, DMSO-d_6_): δ 9.54 (s, 1H), 7.90 (dt, J = 10.2, 2.1 Hz, 1H), 7.77-7.69 (m, 2H), 7.64 (s, 1H), 7.61 (d, J = 8.8 Hz, 1H), 7.49-7.43 (m, 2H), 4.59 (t, J = 8.7 Hz, 2H), 3.98 (s, 3H, CH_3_), 3.50 (t, J = 8.7 Hz, 2H), 2.42 (s, 3H, CH_3_); ^13^C NMR (126 MHz, DMSO-d_6_): δ 162.6 (d, J = 245.9 Hz), 154.2, 145.0 (d, J = 10.1 Hz), 142.8, 133.3, 132.1 (d, J = 9.1 Hz), 131.9, 129.1, 125.3, 124.0, 121.8, 119.4 (d, J = 2.8 Hz), 117.1 (d, J = 20.9 Hz),112.3, 111.1 (d, J = 26.1 Hz), 51.8, 31.0, 21.5, 20.3; HR-ESI-MS (*m*/*z*): [M–Br]^+^ calcd. for C_19_H_18_FN_2_^+^, 293.1449; found, 293.1446.

#### 6,9-Dimethyl-2-(4-fluorophenyl)-3,4-dihydro-β-carbolin-2-ium bromide (**6-4**)

Yield, 76%; yellow powders; mp 257.4–258.2 °C; ^1^H NMR (500 MHz, DMSO-d_6_): δ 9.50 (s, 1H), 7.97-7.93 (m, 2H), 7.63 (s, 1H), 7.60 (d, J = 8.8 Hz, 1H), 7.55 (d, J = 8.8 Hz, 2H), 7.44 (dd, J = 8.8, 1.3 Hz, 1H), 4.57 (t, J = 8.7 Hz, 2H), 3.97 (s, 3H, CH_3_), 3.49 (t, J = 8.7 Hz, 2H), 2.42 (s, 3H, CH_3_); ^13^C NMR (126 MHz, DMSO-*d*_6_): δ 162.7 (d, J = 247.8 Hz), 154.0, 142.4, 140.3(d, J = 2.8 Hz), 132.9, 131.8, 129.0, 125.8 (d, J = 9.1 Hz), 124.5, 124.0, 121.7, 117.2(d, J = 23.5 Hz), 112.2, 52.1, 30.9, 21.5, 20.3; HR-ESI-MS (*m*/*z*): [M–Br]^+^ calcd. for C_19_H_18_FN_2_^+^, 293.1449; found, 293.1446.

#### 6,9-Dimethyl-2-(2-chlorophenyl)-3,4-dihydro-β-carbolin-2-ium bromide (**6-5**)

Yield, 62%; orange powders; mp 292.3–293.1 °C; ^1^H NMR (500 MHz, DMSO-d_6_): δ 9.58 (s, 1H), 8.00-7.96 (m, 1H), 7.85−7.82 (m, 1H), 7.70−7.66 (m, 2H), 7.65 (s, 1H), 7.62 (d, J = 8.8 Hz, 1H), 7.47 (dd, J = 8.9, 1.3 Hz, 1H), 4.42 (t, J = 8.7 Hz, 2H), 3.94 (s, 3H, CH_3_), 3.54 (t, J = 8.7 Hz, 2H), 2.43 (s, 3H, CH_3_); ^13^C NMR (126 MHz, DMSO-d_6_): *δ* 157.3, 142.9, 141.1, 133.6, 132.6, 132.1, 131.3, 129.4, 128.7, 128.6, 125.6, 124.1, 123.3, 121.9, 112.4, 53.0, 30.9, 21.5, 20.5; HR-ESI-MS (*m*/*z*): [M–Br]^+^ calcd. for C_19_H_18_ClN_2_^+^, 309.1153, 309.1124; found, 309.1148, 309.1120.

#### 6,9-Dimethyl-2-(3-chlorophenyl)-3,4-dihydro-β-carbolin-2-ium bromide (**6-6**)

Yield, 55%; orange powders; mp 191.0–192.1 °C; ^1^H NMR (500 MHz, DMSO-d_6_): δ 9.53 (s, 1H), 8.07 (s, 1H), 7.85 (d, J = 7.6 Hz, 1H), 7.73-7.65 (m, 2H), 7.63 (s, 1H), 7.61 (d, J = 8.8 Hz, 1H), 7.45 (d, J = 8.8 Hz, 1H), 4.58 (t, J = 8.7 Hz, 2H), 3.97 (s, 3H, CH_3_), 3.50 (t, J = 8.7 Hz, 2H), 2.42 (s, 3H, CH_3_); ^13^C NMR (126 MHz, DMSO-d_6_): δ 154.3, 144.9, 142.7, 134.5, 133.4, 132.0, 131.9, 130.0, 129.1, 125.3, 124.0, 123.5, 122.0, 121.8, 112.3, 51.8, 30.9, 21.5, 20.3; HR-ESI-MS (*m*/*z*): [M–Br]^+^ calcd. for C_19_H_18_ClN_2_^+^, 309.1154, 309.1124; found, 309.1137, 309.1126.

#### 6,9-Dimethyl-2-(4-chlorophenyl)-3,4-dihydro-β-carbolin-2-ium bromide (**6-7**)

Yield, 73%; red powders; mp 199.9–201.7 °C; ^1^H NMR (500 MHz, DMSO-d_6_): δ 9.53 (s, 1H), 7.94 (d, J = 8.9 Hz, 2H), 7.76 (d, J = 8.9 Hz, 2H), 7.63 (s, 1H), 7.61 (d, J = 8.8 Hz, 1H), 7.44 (d, J = 7.6 Hz, 1H), 4.57 (t, J = 8.7 Hz, 2H), 3.98 (s, 3H, CH_3_), 3.54 (t, J = 8.7 Hz, 2H), 2.42 (s, 3H, CH_3_); ^13^C NMR (126 MHz, DMSO-d_6_): δ 153.9, 142.6, 142.5, 134.6, 133.1, 131.8, 130.2, 129.2, 125.2, 124.9, 124.0, 121.8, 112.2, 51.9, 31.0, 21.5, 20.3; HR-ESI-MS (*m*/*z*): [M–Br]^+^ calcd. for C_19_H_18_ClN_2_^+^, 309.1153, 309.1124; found, 309.1151, 309.1121.

#### 6,9-Dimethyl-2-(2-bromophenyl)-3,4-dihydro-β-carbolin-2-ium bromide (**6-8**)

Yield, 80%; yellow powders; mp 310.1–312.0 °C; ^1^H NMR (500 MHz, DMSO-d_6_): δ 9.59 (s, 1H), 8.00-7.95 (m, 2H), 7.71 (td, J = 7.7, 1.2Hz, 1H), 7.65 (s, 1H), 7.62 (d, J = 8.8 Hz, 1H), 7.61-7.57 (m, 1H), 7.47 (dd, J = 8.8, 1.3 Hz, 1H), 4.41 (t, J = 8.7 Hz, 2H), 3.94 (s, 3H, CH_3_), 3.56 (t, J = 8.7 Hz, 2H), 2.43 (s, 3H, CH_3_); ^13^C NMR (126 MHz, DMSO-d_6_): δ 157.4, 142.9, 142.7, 134.3, 133.6, 132.7, 132.1, 130.0, 128.7, 128.6, 125.4, 124.2, 121.8, 118.8, 112.4, 53.2, 31.0, 21.5, 20.6; HR-ESI-MS (*m*/*z*): [M–Br]^+^ calcd. for C_19_H_18_BrN_2_^+^, 353.0648, 355.0628; found, 353.0649, 355.0628.

#### 6,9-Dimethyl-2-(3-bromophenyl)-3,4-dihydro-β-carbolin-2-ium bromide (**6-9**)

Yield, 58%; orange powders; mp 184.3–185.6 °C; ^1^H NMR (500 MHz, DMSO-d_6_): δ 9.53 (s, 1H), 8.19 (t, J = 1.9 Hz, 1H), 7.89 (dd, J = 8.1, 1.7 Hz, 1H), 7.80 (d, J = 8.0 Hz, 1H), 7.63 (dd, J = 7.9, 2.8 Hz, 2H) 7.60 (d, J = 3.4 Hz, 1H), 7.45 (dd, J = 8.8, 1.2Hz, 1H), 4.57 (t, J = 8.7 Hz, 2H), 3.97 (s, 3H, CH_3_), 3.49 (t, J = 8.7 Hz, 2H), 2.42 (s, 3H, CH_3_); ^13^C NMR (126 MHz, DMSO-d_6_): δ 154.2, 145.0, 142.7, 133.3, 133.0, 132.1, 132.0, 129.1, 126.3, 125.3, 124.0, 122.8, 122.4, 121.8, 112.3, 51.9, 31.0, 21.5, 20.3; HR-ESI-MS (*m*/*z*): [M–Br]^+^ calcd. for C_19_H_18_BrN_2_^+^, 353.0648, 355.0628; found, 353.0610, 355.0632.

#### 6,9-Dimethyl-2-(4-bromophenyl)-3,4-dihydro-β-carbolin-2-ium bromide (**6-10**)

Yield, 90%; orange powders; mp 201.0–202.1 °C; ^1^H NMR (500 MHz, DMSO-d_6_): δ 9.54 (s, 1H), 7.88 (m, 4 H), 7.62 (s, 1H), 7.60 (d, J = 8.8 Hz, 1H), 7.43 (d, J = 8.6 Hz, 1H), 4.57 (t, J = 8.7 Hz, 2H), 3.98 (s, 3H, CH_3_), 3.49 (t, J = 8.7 Hz, 2H), 2.42 (s, 3H, CH_3_); ^13^C NMR (126 MHz, DMSO-d_6_): δ 153.8, 153.7, 143.0, 142.6, 133.1, 131.8, 129.2, 128.5, 125.9, 125.4, 124.9, 124.0, 123.1, 121.8, 112.2, 51.8, 31.1, 21.5, 20.3; HR-ESI-MS (*m*/*z*): [M–Br]^+^ calcd. for C_19_H_18_BrN_2_^+^, 353.0648, 355.0628; found, 353.0628, 355.0615.

#### 6,9-Dimethyl-2-(2-iodophenyl)-3,4-dihydro-β-carbolin-2-ium bromide (**6-11**)

Yield, 78%; orange powders; mp 330.4–331.8 °C; ^1^H NMR (500 MHz, DMSO-d_6_): δ 9.59 (s, 1H), 8.15 (dd, J = 7.9, 0.9 Hz, 1H), 7.93 (dd, J = 7.9, 1.2 Hz, 1H), 7.70 (td, J = 7.8, 1.0 Hz, 1H), 7.66 (s, 1H), 7.62 (d, J = 8.8 Hz, 1H), 7.47 (dd, J = 8.8, 1.0 Hz, 1H), 7.40 (td, J = 7.8, 1.3 Hz, 1H), 4.38 (t, J = 8.7 Hz, 2H), 3.95 (s, 3H, CH_3_), 3.61 (t, J = 8.7 Hz, 2H), 2.43 (s, 3H, CH_3_); ^13^C NMR (126 MHz, DMSO-d_6_): δ 157.3, 146.2, 142.8, 140.4, 133.5, 132.5, 132.1, 130.5, 128.6, 127.8, 125.0, 124.2, 121.8, 112.4, 96.4, 53.4, 31.0, 21.5, 20.8; HR-ESI-MS (*m*/*z*): [M–Br]^+^ calcd. for C_19_H_18_IN_2_^+^, 401.0509; found, 401.0511.

#### 6,9-Dimethyl-2-(3-iodophenyl)-3,4-dihydro-β-carbolin-2-ium bromide (**6-12**)

Yield, 62%; orange powders; mp 276.7–277.5 °C; ^1^H NMR (500 MHz, DMSO-d_6_): δ 9.51 (s, 1H), 8.31-8.27 (m, 1H), 7.95 (d, J = 7.9 Hz, 1H), 7.88 (dd, J = 8.1, 1.9 Hz, 1H), 7.62 (d, J = 5.2 Hz, 1H), 7.61 (d, J = 8.8 Hz, 1H), 7.47-7.42 (m, 2H), 4.56 (t, J = 8.7 Hz, 2H), 3.97 (s, 3H, CH_3_), 3.48 (t, J = 8.7 Hz, 2H), 2.42 (s, 3H, CH_3_); ^13^C NMR (126 MHz, DMSO-d_6_): δ 154.1, 144.8, 142.6, 138.8, 133.2, 132.0, 131.9, 131.6, 129.1, 125.1, 124.0, 122.7, 121.8, 112.3, 95.9, 51.5, 31.0, 21.5, 20.3; HR-ESI-MS (*m*/*z*): [M–Br]^+^ calcd. for C_19_H_18_IN_2_^+^, 401.0509; found, 401.0515.

#### 6,9-Dimethyl-2-(4-iodophenyl)-3,4-dihydro-β-carbolin-2-ium bromide (**6-13**)

Yield, 51.6%; orange powder; mp 294.1–295.4 °C; ^1^H NMR (500 MHz, DMSO-d_6_): δ 9.51 (s, 1H), 8.04 (d, J = 8.7 Hz, 2H), 7.69 (d, J = 8.7 Hz, 2H), 7.63 (s, 1H), 7.60 (d, J = 8.8 Hz, 1H), 7.44 (d, J = 8.8 Hz, 1H), 4.56 (t, J = 8.7 Hz, 2H), 3.97 (s, 3H, CH_3_), 3.48 (t, J = 8.7 Hz, 2H), 2.42 (s, 3H, CH_3_); ^13^C NMR (126 MHz, DMSO-d_6_): δ 153.6, 143.5, 142.6, 139.0, 133.1, 131.8, 129.2, 125.2, 124.9, 124.0, 121.8, 112.2, 96.7, 51.7, 31.0, 21.5, 20.3; HR-ESI-MS (*m*/*z*): [M–Br]^+^ calcd. for C_19_H_18_IN_2_^+^, 401.0509; found, 401.0512.

#### 6,9-Dimethyl-2-(2-methylphenyl)-3,4-dihydro-β-carbolin-2-ium bromide (**6-14**)

Yield, 91%; orange powders; mp 168.4–169.5 °C; ^1^H NMR (500 MHz, DMSO-d_6_): δ 9.45 (s, 1H), 7.70 (d, J = 7.7 Hz, 1H), 7.65 (s, 1H), 7.61 (d, J = 8.8 Hz, 1H), 7.53 (d, J = 4.3 Hz, 2H), 7.48 (dd, J = 7.9, 3.9 Hz, 1H), 7.44 (dd, J = 8.8, 1.3 Hz, 1H), 4.39 (t, J = 8.7 Hz, 2H), 3.93 (s, 3H, CH_3_), 3.51 (t, J = 8.7 Hz, 2H), 2.45 (s, 3H, CH_3_), 2.43 (s, 3H, CH_3_); ^13^C NMR (126 MHz, DMSO-d_6_): δ 156.6, 143.3, 142.3, 133.2, 132.8, 132.2, 131.8, 130.8, 128.7, 127.9, 126.4, 124.4, 124.1, 121.7, 112.2, 52.9, 30.9, 21.5, 20.3, 17.8; HR-ESI-MS (*m*/*z*): [M–Br]^+^ calcd. for C_20_H_21_N_2_^+^, 289.1699; found, 289.1689.

#### 6,9-Dimethyl-2-(3-methylphenyl)-3,4-dihydro-β-carbolin-2-ium bromide (**6-15**)

Yield, 90%; orange powders; mp 174.0–175.4 °C; ^1^H NMR (500 MHz, DMSO-d_6_): δ 9.49 (s, 1H), 7.73 (s, 1H), 7.69-7.65 (m, 1H), 7.63 (s, 1H), 7.60 (d, J = 8.8 Hz, 1H), 7.55 (t, J = 7.8 Hz, 1H), 7.42 (t, J = 8.8 Hz, 2H), 4.58 (t, J = 8.7 Hz, 2H), 3.98 (s, 3H, CH_3_), 3.49 (t, J = 8.7 Hz, 2H), 2.45 (3H, s, CH_3_), 2.42 (s, 3H, CH_3_); ^13^C NMR (126 MHz, DMSO-d_6_): δ 153.5, 143.8, 142.4, 140.1, 132.9, 131.8, 130.9, 130.1, 129.1, 124.5, 124.0, 123.5, 121.7, 120.2, 112.2, 51.9, 30.9, 21.5, 21.4, 20.3; HR-ESI-MS (*m*/*z*): [M–Br]^+^ calcd. for C_20_H_21_N_2_^+^, 289.1699; found, 289.1703.

#### 6,9-Dimethyl-2-(4-methylphenyl)-3,4-dihydro-β-carbolin-2-ium bromide (**6-16**)

Yield, 82%; orange powders; mp 135.8–136.5 °C; ^1^H NMR (500 MHz, DMSO-d_6_): δ 9.45 (s, 1H), 7.76 (d, *J* = 8.5 Hz, 2H), 7.62 (s, 1H), 7.60 (d, J = 8.8 Hz, 1H), 7.47 (d, J = 8.3 Hz, 2H), 7.43 (d, J = 8.8 Hz, 1H), 4.57 (t, J = 8.7 Hz, 2H), 3.96 (s, 3H, CH_3_), 3.48 (t, J = 8.7 Hz, 2H), 2.42 (d, 6 H, CH_3_); ^13^C NMR (126 MHz, DMSO-d_6_): δ 153.2, 142.3, 141.5, 140.3, 132.7, 131.7, 130.6, 129.1, 128.5, 126.0, 124.2, 124.0, 122.9, 121.7, 112.2, 51.9, 30.9, 21.5, 21.2, 20.2; HR-ESI-MS (*m*/*z*): [M–Br]^+^ calcd. for C_20_H_21_N_2_^+^, 289.1699; found, 289.1694.

#### 6,9-Dimethyl-2-(2-methoxylphenyl)-3,4-dihydro-β-carbolin-2-ium bromide (**6-17**)

Yield, 70%; orange powders; mp 279.0–280.1 °C; ^1^H NMR (500 MHz, DMSO-d_6_): δ 9.46 (s, 1H), 7.78 (dd, J = 7.8, 1.5 Hz, 1H), 7.65-7.57 (m, 3H), 7.44 (d, J = 9.9 Hz, 1H), 7.37 (d, *J* = 8.0 Hz, 1H), 7.20 (t, J = 7.6 Hz, 1H), 4.37 (t, J = 8.6 Hz, 2H), 3.95 (s, 3H, CH_3_), 3.94 (s, 3H, CH_3_), 3.46 (t, J = 8.6 Hz, 2H), 2.43 (s, 3H, CH_3_); ^13^C NMR (126 MHz, DMSO-d_6_): δ 156.3, 152.9, 142.3, 132.9, 132.5, 132.3, 131.8, 128.8, 126.9, 124.7, 124.1, 121.7, 121.5, 113.7, 112.2, 57.0, 52.8, 30.9, 21.5, 20.4; HR-ESI-MS (*m*/*z*): [M–Br]^+^ calcd. for C_20_H_21_N_2_O^+^, 305.1648; found, 305.1654.

#### 6,9-Dimethyl-2-(3-methoxylphenyl)-3,4-dihydro-β-carbolin-2-ium bromide (**6-18**)

Yield, 70%; orange powders; mp 241.9−242.9 °C; ^1^H NMR (500 MHz, DMSO-d_6_): δ 9.50 (1H, s), 7.63 (s, 1H), 7.60 (t, J = 6.6 Hz, 1H), 7.57 (t, J = 8.2Hz, 1H,), 7.52 (t, J = 2.2Hz, 1H), 7.45-7.41 (m, 2H), 7.17 (dd, J = 8.3, 2.2 Hz, 1H), 4.59 (t, J = 8.6 Hz, 2H), 3.98 (s, 3H, CH_3_), 3.89 (s, 3H, CH_3_), 3.49 (t, J = 8.6 Hz, 2H), 2.42 (s, 3H, CH_3_); ^13^C NMR (126 MHz, DMSO-d_6_): δ 160.6, 153.8, 145.0, 142.4, 132.9, 131.8, 131.2, 129.1, 124.7, 124.0, 121.8, 115.9, 115.1, 112.2, 109.3, 56.4, 52.0, 31.0, 21.5, 20.3; HR-ESI-MS [M–Br]^+^: Calcd for C_20_H_21_N_2_O^+^, 305.1648; found, 305.1646.

#### 6,9-Dimethyl-2-(4-methoxylphenyl)-3,4-dihydro-β-carbolin-2-ium bromide (**6-19**)

Yield, 83%; orange powders; mp 204.5−205.8 °C; ^1^H NMR (500 MHz, DMSO-d_6_): δ 9.42 (s, 1H), 7.82 (d, J = 8.9 Hz, 2H), 7.62 (s, 1H), 7.59 (d, J = 8.8 Hz, 1H), 7.42 (d, J = 8.7 Hz, 1H), 7.20 (d, J = 8.9 Hz, 2H), 4.56 (t, J = 8.6 Hz, 2H), 3.96 (s, 3H, CH_3_), 3.86 (s, 3H, CH_3_), 3.47 (t, J = 8.6 Hz, 2H), 2.42 (s, 3H, CH_3_); ^13^C NMR (126 MHz, DMSO-d_6_): δ 160.6, 152.8, 142.1, 136.9, 132.5, 131.6, 129.1, 128.5, 126.0, 124.7, 124.0, 123.7, 121.6, 115.3, 112.1, 56.3, 52.2, 30.9, 21.5, 20.2; HR-ESI-MS (*m*/*z*): [M–Br]^+^ calcd. for C_20_H_21_N_2_O^+^, 305.1648; found, 305.1643.

#### 6,9-Dimethyl-2-(2-hydroxyphenyl)-3,4-dihydro-β-carbolin-2-ium bromide (**6-20**)

Yield, 82.0%; yellow powder; mp 259.1–261.3 °C; ^1^H NMR (500 MHz, DMSO-d_6_): δ 10.87 (s, 1H, OH), 9.45 (s, 1H), 7.71 (dd, J = 7.9, 1.3 Hz, 1H, Ar-H), 7.62 (s 1H), 7.60 (d, J = 8.9 Hz, 1H), 7.46-7.40 (m, 2H), 7.17 (d, J = 8.2 Hz, 1H), 7.05 (t, J = 7.6 Hz, 1H), 4.39 (t, J = 8.7 Hz, 2H), 3.94 (s, 3H, CH_3_), 3.45 (t, J = 8.7 Hz, 2H), 2.42 (s, 3H, CH_3_); ^13^C NMR (125 MHz, DMSO-d_6_): δ 155.9, 151.3, 142.2, 132.7, 132.0, 131.7, 131.5, 128.8, 126.8, 124.3, 124.1, 121.6, 120.2, 117.6, 112.2, 52.6, 30.9, 21.5, 20.4; HR-ESI-MS (*m*/*z*): [M–Br]^+^ calcd. for C_19_H_19_N_2_O^+^, 291.1492; found, 291.1497.

#### 6,9-Dimethyl-2-(3-hydroxyphenyl)-3,4-dihydro-β-carbolin-2-ium bromide (**6-21**)

Yield, 99%; orange powders; mp 296.7–298.3 °C; ^1^H NMR (500 MHz, DMSO-d_6_): δ 10.18 (s, 1H, OH), 9.44 (s, 1H), 7.62 (s, 1H), 7.60 (d, J = 8.8 Hz, 1H), 7.47-7.40 (m, 2H), 7.27 (dd, J = 7.9, 1.8 Hz, 1 H), 7.22 (t, J = 2.1 Hz, 1H), 7.00 (dd, J = 8.2, 1.8 Hz, 1H), 4.55 (t, J = 8.7 Hz, 2H), 3.96 (s, 3H, CH_3_), 3.47 (t, J = 8.7 Hz, 2H), 2.42 (s, 3H, CH_3_); ^13^C NMR (126 MHz, DMSO-d_6_): δ 158.9, 153.5, 145.0, 142.4, 132.9, 131.7, 131.1, 129.1, 124.5, 124.0, 121.7, 117.3, 113.5, 112.2, 110.1, 51.9, 30.9, 21.5, 20.3; HR-ESI-MS (*m*/*z*): [M–Br]^+^ calcd. for C_19_H_19_N_2_O^+^, 291.1492; found, 291.1490.

#### 6,9-Dimethyl-2-(4-hydroxyphenyl)-3,4-dihydro-β-carbolin-2-ium bromide (**6-22**)

Yield, 82%; yellow powders; mp 315.4–316.5 °C; ^1^H NMR (500 MHz, DMSO-d_6_): δ 10.21 (s, 1H, OH), 9.38 (s, 1H), 7.72-7.68 (m, 2H), 7.61 (s, 1H), 7.58 (d, J = 8.8 Hz, 1H), 7.40 (dd, J = 8.8, 1.3 Hz, 1H), 7.01-6.97 (m, 2H), 4.53 (t, J = 8.7 Hz, 2H), 3.96 (s, 3H, CH_3_), 3.46 (t, J = 8.7 Hz, 2H), 2.42 (s, 3H, CH_3_); ^13^C NMR (126 MHz, DMSO-d_6_): δ 159.3, 152.2, 142.0, 135.6, 132.3, 131.6, 129.1, 124.7, 124.0, 123.4, 121.6, 116.5, 112.1, 52.2, 30.9, 21.5, 20.2; HR-ESI-MS (*m*/*z*): [M–Br]^+^ calcd. for C_19_H_19_N_2_O^+^, 291.1492; found, 291.1487.

#### 6,9-Dimethyl-2-(3-trifluoromethylphenyl)-3,4-dihydro-β-carbolin-2-ium bromide (**6-23**)

Yield, 63%; yellow powders; mp 295.8–296.3 °C; ^1^H NMR (500 MHz, DMSO-d_6_): δ 9.62 (s, 1H), 8.35 (s, 1H), 8.22 (d, J = 8.7 Hz, 1H), 7.97 (d, J = 7.9 Hz, 1H), 7.91 (t, J = 7.9 Hz, 1H), 7.65 (s, 1H), 7.62 (d, J = 8.8 Hz, 1H), 7.45 (dd, J = 8.8, 1.3 Hz, 1H), 4.63 (t, J = 8.7 Hz, 2H), 3.99 (s, 3H, CH_3_), 3.52 (t, J = 8.7 Hz, 2H), 2.43 (s, 3H, CH_3_); ^13^C NMR (126 MHz, DMSO-d_6_): δ 154.6, 144.4, 142.7, 133.3, 131.9, 131.6, 130.8 (d, J = 32.7 Hz), 129.2, 127.6, 126.7, 125.4, 125.2, 124.0, 121.9, 120.7 (d, J = 3.9 Hz), 112.3, 52.0, 31.1, 21.5, 20.3; HR-ESI-MS (*m*/*z*): [M–Br]^+^ calcd. for C_20_H_18_F_3_N_2_^+^, 343.1417; found, 343.1427.

#### 6,9-Dimethyl-2-(4-trifluoromethylphenyl)-3,4-dihydro-β-carbolin-2-ium bromide (**6-24**)

Yield, 37%; yellow powders; mp 255.4–256.6 °C; ^1^H NMR (500 MHz, DMSO-d_6_): δ 9.59 (s, 1H), 8.09 (q, J = 8.8 Hz, 4 H), 7.65 (s, 1H), 7.62 (d, J = 8.8 Hz, 1H), 7.49-7.44 (m, 1H), 4.63 (t, J = 8.7 Hz, 2H), 3.98 (s, 3H, CH_3_), 3.52 (t, J = 8.7 Hz, 2H), 2.43 (s, 3H, CH_3_); ^13^C NMR (126 MHz, DMSO-d_6_): δ 154.5, 146.8, 142.9, 133.6, 132.0,130.0, 129.3, 127.5, 127.4, 125.5, 124.2, 124.0, 121.9, 112.3, 51.7, 31.0, 21.5, 20.3; HR-ESI-MS (*m*/*z*): [M–Br]^+^ calcd. for C_20_H_18_F_3_N_2_^+^, 343.1417; found, 343.1432.

#### 6,9-Dimethyl-2-(3-nitrophenyl)-3,4-dihydro-β-carbolin-2-ium bromide (**6-25**)

Yield, 68%; yellow powders; mp 254.1–255.8 °C; ^1^H NMR (500 MHz, DMSO-d_6_): δ 9.69 (s, 1H), 8.81 (t, J = 2.0 Hz, 1H), 8.42 (dd, J = 8.2, 1.6 Hz, 1H), 8.38 (dd, J = 8.1, 1.7 Hz, 1H), 7.97 (t, J = 8.2 Hz, 1H). 7.65 (s, 1H), 7.62 (d, J = 8.8 Hz, 1H), 7.46 (d, J = 8.8 Hz, 1H), 4.64 (t, J = 8.7 Hz), 4.00 (s, 3H, CH_3_), 3.54 (t, J = 8.7 Hz, 2H), 2.43 (s, 3H, CH_3_); ^13^C NMR (126 MHz, DMSO-d_6_): δ 154.8, 148.7, 144.7, 142.9, 133.5, 132.0, 131.8, 130.0, 129.2, 125.7, 124.6, 124.1, 121.9, 119.0, 112.3, 52.0, 31.1, 21.5, 20.4; HR-ESI-MS (*m*/*z*): [M–Br]^+^ calcd. for C_19_H_18_N_3_O_2_^+^, 320.1394; found, 320.1395.

#### 6,9-Dimethyl-2-(3-acetylphenyl)-3,4-dihydro-β-carbolin-2-ium bromide (**6-26**)

Yield, 57%; orange powders; mp 242.2–243.3 °C; ^1^H NMR (500 MHz, DMSO-d_6_): δ 9.62 (s, 1H, H-1), 8.45-8.41 (m, 1H), 8.17-8.13 (m, 2H), 7.83 (t, J = 7.9 Hz, 1H), 7.64 (s, 1H), 7.62 (d, J = 8.8 Hz, 1H), 7.45 (dd, J = 8.8, 1.2 Hz, 1H,), 4.63 (t, J = 8.7 Hz, 2H), 4.00 (s, 3H, CH_3_), 3.53 (t, J = 8.7 Hz, 2H), 2.71 (s, 3H, CH_3_), 2.43 (s, 3H, CH_3_); ^13^C NMR (126 MHz, DMSO-d_6_): δ 197.6, 154.3, 144.2, 142.6, 138.6, 133.1, 131.8, 130.8, 129.7, 129.2, 127.8, 125.0, 124.0, 123.1, 121.8, 112.3, 52.0, 31.1, 27.6, 21.5, 20.3; HR-ESI-MS (*m*/*z*): [M–Br]^+^ calcd. for C_21_H_21_N_2_O^+^, 317.1648; found, 317.1634.

#### 6,9-Dimethyl-2-(2,6-difluorophenyl)-3,4-dihydro-β-carbolin-2-ium bromide (**6-27**)

Yield, 48%; red powders; mp 288.6–289.9 °C; ^1^H NMR (500 MHz, DMSO-d_6_): δ 9.72 (s, 1H), 7.78-7.70 (m, 1H), 7.65 (s, 1H), 7.63 (d, J = 8.9 Hz, 1H), 7.51 (dd, J = 16.6, 8.2 Hz, 3H), 4.47 (t, J = 8.6 Hz, 2H), 3.94 (s, 3H, CH_3_), 3.52 (t, J = 8.6 Hz, 2H), 2.42 (s, 3H,CH_3_); ^13^C NMR (126 MHz, DMSO-d_6_): δ 158.3, 156.7 (d, J = 252.8 Hz), 143.7, 134.5, 132.9, 132.4, 128.9, 127.3, 124.3, 122.1, 113.7, 113.6, 112.6, 52.7, 31.0, 21.4, 20.6; HR-ESI-MS (*m*/*z*): [M–Br]^+^ calcd. for C_19_H_17_F_2_N_2_^+^, 311.1354; found, 311.1360.

#### 6,9-Dimethyl-2-(2,4-dichlorophenyl)-3,4-dihydro-β-carbolin-2-ium bromide (**6-28**)

Yield, 45%; orange powders; mp 282.1–283.3 °C; ^1^H NMR (500 MHz, DMSO-d_6_): δ 9.58 (s, 1H), 8.09-8.03 (m, 2H,), 7.80 (dd, J = 8.6, 2.3 Hz, 1H), 7.64 (s, 1H), 7.62 (d, J = 8.9 Hz, 1H), 7.47 (dd, J = 8.8, 1.1 Hz, 1H), 4.40 (t, J = 8.7 Hz, 2H), 3.94 (s, 3H, CH_3_), 3.52 (t, J = 8.7 Hz, 2H), 2.43 (s, 3H, CH_3_); ^13^C NMR (126 MHz, DMSO-d_6_): δ 157.4, 143.0, 140.0, 136.2, 133.8, 132.1, 130.7, 130.3, 130.0, 129.5, 128.7, 125.9, 124.2, 121.9, 112.4, 52.9, 31.0, 21.5, 20.5; HR-ESI-MS (*m*/*z*): [M–Br]^+^ calcd. for C_19_H_17_Cl_2_N_2_^+^, 343.0764, 345.0734, found 343.0769, 345.0736.

#### 6,9-Dimethyl-2-(3,5-dichlorophenyl)-3,4-dihydro-β-carbolin-2-ium bromide (**6-29**)

Yield, 53%; yellow powders; mp 279.0–280.1 °C; ^1^H NMR (500 MHz, DMSO-d_6_): δ 9.58 (s, 1H), 8.10 (d, J = 1.5 Hz, 2H), 7.87 (s, 1H), 7.63 (s, 1H), 7.61 (d, J = 8.9 Hz, 1H), 7.46 (d, J = 8.7 Hz, 1H), 4.56 (t, J = 8.7 Hz, 2H), 3.98 (s, 3H, CH_3_), 3.49 (t, J = 8.7 Hz, 2H), 2.42 (s, 3H, CH_3_); ^13^C NMR (126 MHz, DMSO-d_6_): δ 154.5, 145.5, 143.0, 135.4, 133.7, 132.0, 129.5, 129.1, 126.0, 124.1, 122.6, 121.9, 112.4, 51.8, 31.0, 21.5, 20.3; HR-ESI-MS (*m*/*z*): [M–Br]^+^ calcd. for C_19_H_17_Cl_2_N_2_^+^, 343.0764, 345.0734; found, 343.0662, 345.0687.

#### 6,9-Dimethyl-2-(2,4-dibromophenyl)-3,4-dihydro-β-carbolin-2-ium bromide (**6-30**)

Yield, 35%; yellow powders; mp 269.1–270.8 °C; ^1^H NMR (500 MHz, DMSO-d_6_): δ 9.59 (s, 1H), 8.28 (d, J = 1.8 Hz, 1H1H), 7.99-7.95 (m, 2H), 7.64 (s, 1H), 7.62 (d, J = 8.8 Hz, 1H), 7.47 (dd, J = 8.8, 1.2 Hz, 1H), 4.38 (t, J = 8.7 Hz, 2H), 3.94 (s, 3H, CH_3_), 3.56 (t, J = 8.7 Hz, 2H), 2.43 (s, 3H, CH_3_); ^13^C NMR (126 MHz, DMSO-d_6_): δ 157.3, 143.0, 142.0, 136.2, 133.7, 132.9, 132.1, 130.2, 128.6, 125.6, 124.7, 124.2, 121.8, 120.5, 112.4, 53.0, 31.0, 21.5, 20.6; HR-ESI-MS (*m*/*z*): [M–Br]^+^ calcd. for C_19_H_17_Br_2_N_2_^+^, 432.9733, 430.9753, 434.9712; found, 432.9716, 430.9753, 434.9692.

#### 6,9-Dimethyl-2-(2-fluoro-4-bromophenyl)-3,4-dihydro-β-carbolin-2-ium bromide (**6-31**)

Yield, 63%; orange powders; mp 237.0–238.1 °C; ^1^H NMR (500 MHz, DMSO-d_6_): δ 9.56 (s, 1H), 8.02 (dd, J = 10.5, 2.0 Hz, 1H,Ar-H), 7.94 (t, J = 8.5 Hz, 1H), 7.76 (dd, J = 8.6, 1.2 Hz, 1H), 7.63 (s, 1H), 7.62 (d, J = 9.0 Hz, 1H), 7.47 (dd, J = 8.8, 1.1 Hz, 1 H), 4.46 (t, J = 8.7 Hz, 2H), 3.95 (s, 3H, CH_3_), 3.48 (t, J = 8.7 Hz, 2H), 2.42 (s, 3H, CH_3_); ^13^C NMR (126 MHz, DMSO-d_6_): δ 156.5, 155.4 (d, J = 256.1 Hz), 143.0, 133.7, 132.1, 131.0 (d, J = 10.0 Hz), 129.3 (d, J = 3.5 Hz), 129.03, 129.01, 126.0, 124.1, 124.0 (d, J = 9.0 Hz), 121.9, 121.1 (d, J = 22.7 Hz), 112.4, 52.6, 31.0, 21.4, 20.4; HR-ESI-MS (*m*/*z*): [M–Br]^+^ calcd. for C_19_H_17_BrFN_2_^+^, 371.0554, 373.0534; found, 371.0534, 373.0528.

#### 7-Fluoro-9-methyl-2-phenyl-3,4-dihydro-β-carbolin-2-ium bromide (**6-32**)

Yield, 68%; orange powders; mp 173.4–174.5; ^1^H NMR (500 MHz, DMSO-d_6_): δ 9.53 (s, 1H, H-1), 7.96 (dd, J = 9.0, 5.6 Hz, 1H, Ar-H), 7.89 (d, J = 7.9 Hz, 2H, Ar-H), 7.67 (t, J = 7.8 Hz, 2H), 7.61 (d, J = 7.0 Hz, 1H), 7.45 (dt, J = 7.7, 3.7 Hz, 1H) 7.18 (td, J = 9.3, 2.1 Hz), 4.61 (t, J = 8.8 Hz, 2H), 3.97 (s, 3H, CH_3_), 3.54 (t, J = 8.8 Hz, 2H); ^13^C NMR (126 MHz, DMSO-d_6_): δ 164.2 (d, J = 247.7 Hz), 153.7, 143.8, 130.4, 130.3, 130.2 (d, J = 3.5 Hz), 128.5, 126.1, 126.0, 125.6, 125.5, 123.2, 120.9, 112.7 (d, J = 26.3 Hz), 98.3 (d, J = 27.2 Hz), 51.8, 31.2, 20.3; HR-ESI-MS (*m*/*z*): [M–Br]^+^ calcd. for C_18_H_16_FN_2_^+^, 279.1292; found, 279.1292.

#### 6-Methoxy-9-methyl-2-phenyl-3,4-dihydro-β-carbolin-2-ium bromide (**6-33**)

Yield, 88%; orange powders; mp 208.9–209.4 °C; ^1^H NMR (500 MHz, DMSO-d_6_): δ 9.48 (s, 1H), 7.87 (d, J = 8.1 Hz, 2H), 7.67 (t, J = 7.8 Hz, 2H), 7.63 (d, J = 9.2 Hz, 1H), 7.59 (t, J = 7.4 Hz, 1H), 7.29-7.24 (m, 2H), 4.60 (t, J = 8.7 Hz, 2H), 3.97 (s, 3H, CH_3_), 3.84 (s, 3H, CH_3_), 3.49 (t, J = 8.7 Hz, 2H); ^13^C NMR (126 MHz, DMSO-d_6_): δ 155.6, 153.3, 143.9, 139.9, 130.3, 130.2, 130.1, 129.3, 128.5, 124.2, 124.0, 123.7, 123.1, 113.7, 101.2, 56.1, 52.0, 31.0, 20.3; HR-ESI-MS (*m*/*z*): [M–Br]^+^ calcd. for C_19_H_19_N_2_O^+^, 291.1492; found, 291.1509.

#### 9-methyl-2-phenyl-3,4-dihydro-β-carbolin-2-ium bromide (**6-34**)

Yield, 94%; orange powders; The NMR data and melting point were consistent with those previously reported by us^19^.

#### Synthesis of 7-fluoro-9-methyl-2-phenyl-β-carbolin-2-ium bromide (**7**)

To a solution of compound **6-32** (0.2 mmol) in acetonitrile (40 mL) was added 5% Pd/C (wetted with ca. 55% water) (0.17 mmol, 80 mg). The mixture was refluxed at 80 °C for about 3 days to complete the reaction. The Pd/C powders in the reaction solution was filtered off through a sand core funnel and completely washed with methanol. The combined solution was evaporated under vacuum to yield the desired compound **7**. The ^1^H and ^13^C NMR data matched those previously reported by us^21^.

### Antifungal Assay

Antifungal activity was determined according to the method previously reported by us^11^. Briefly, the solution of 10 mL of the compound in 4% DMSO aqueous solution (v/v) was fully mixed with 150 mL of melted PDA agar (50 °C) to provide a medium containing 150 μM of the compounds and 0.25% DMSO (v/v). The mixture was poured into a sterilized Petri dish (ca. 20 mL each plate). Thiabendazole, azoxystrobin and carbendazim (150 μM) were used as positive controls. The medium only containing 0.25% DMSO served as a blank control and no observable effect on the growth of tested fungi was found. A fungus disc (d = 5 mm) cut from beforehand subcultured Petri dishes was placed at the center of the partially solidified medium. The dishes were kept in an incubator at 25 °C for 72 h. Each experiment was conducted in triplicate. The growth inhibition rates were calculated according our previously reported^11^.

A set of stork solutions with different concentrations of the compound in 5% DMSO aqueous solution was prepared by double-fold dilution method and used to determine median effective concentrations (IC_50_). According to the same procedure described above, each the stock solution (10 mL) was determined in triplicate for the growth inhibition rates against the fungi. Antifungal toxicity regression equations and the corresponding IC_50_ values were obtained by using PRISM software ver. 7.00 (GraphPad Software Inc., San Diego, CA, USA)^11^. Significant difference among IC_50_ values of various compounds against the same fungus species was analyzed by Duncan’s multiple comparisons using PRISM software ver. 7.0.

## Supplementary information

SUPPLEMENTARY DATASET

## Data Availability

Data will be made available upon request to the corresponding authors.
