# Supplementary material for: New 2-aryl-6-methyl-3,4-dihydro-β-carbolin-2-iums as potential antifungal agents: Synthesis, bioactivity and structure-activity relationship
Source: Sci Rep. 2019 Feb 13;9:1941. doi: 10.1038/s41598-018-38222-x (PMC6374477; doi:10.1038/s41598-018-38222-x)
Supplement: Supplementary file 1 — SUPPLEMENTARY DATASET [file 41598_2018_38222_MOESM1_ESM.pdf]

Supporting information

New 2-aryl-6-methyl-3,4-dihydro-β-carbolin-2-iums as potential antifungal agents: Synthesis, bioactivity and structure-activity relationship

Xingqiang Li, Bingyu Zhang, Wei Zhao, Shanshan Yang, Xinjuan Yang* and Le Zhou*

College of Chemistry & Pharmacy, Northwest A&F University, Yangling 712100, Shaanxi Province, People's Republic of China

Correspondence and requests for materials should be addressed to L.Z. (email: zhoulechem@nwsuaf.edu.cn) or X.Y. (email: yxjsn2@163.com).

Xingqiang Li and Bingyu Zhang contributed equally to this work.

Contents

^{1}H NMR and ^{13}C NMR spectra of compounds 1a, 5a……………………………………………….3

^{1}H NMR and ^{13}C NMR spectra of compounds 6-1 to 6-33………………………………………….5

HRMS of 6-1 to 6-33……………………………………………………………………………38

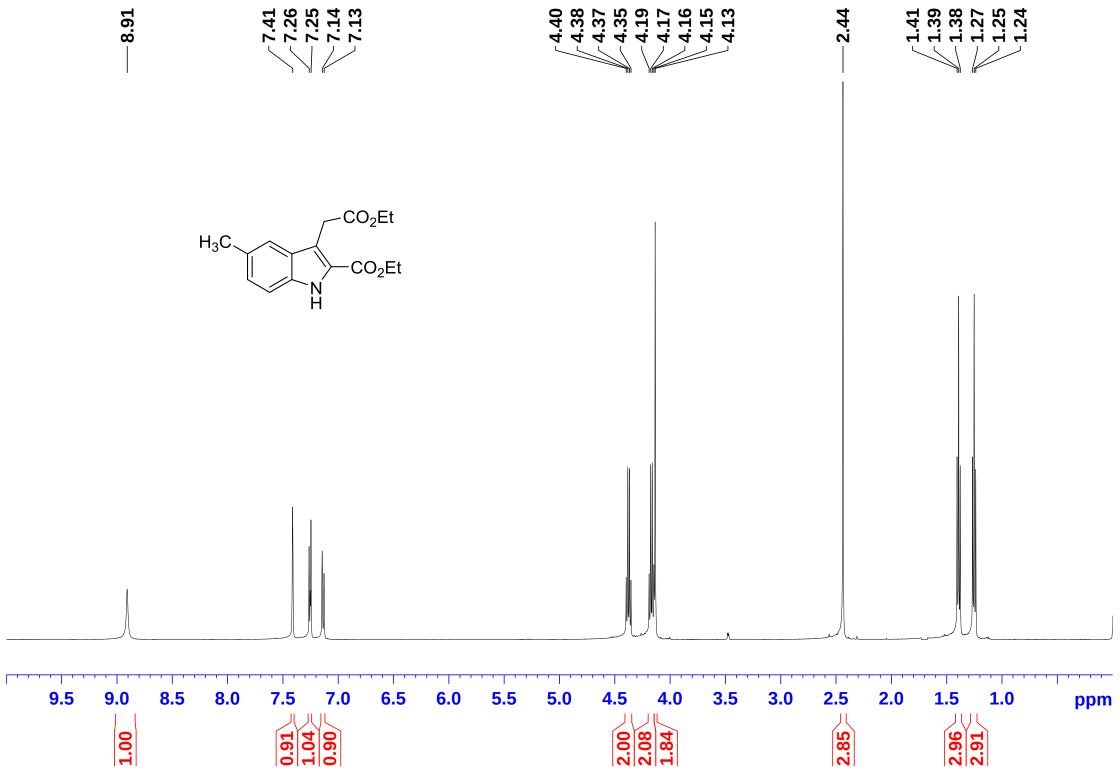

^{1}H NMR of Compound 1a

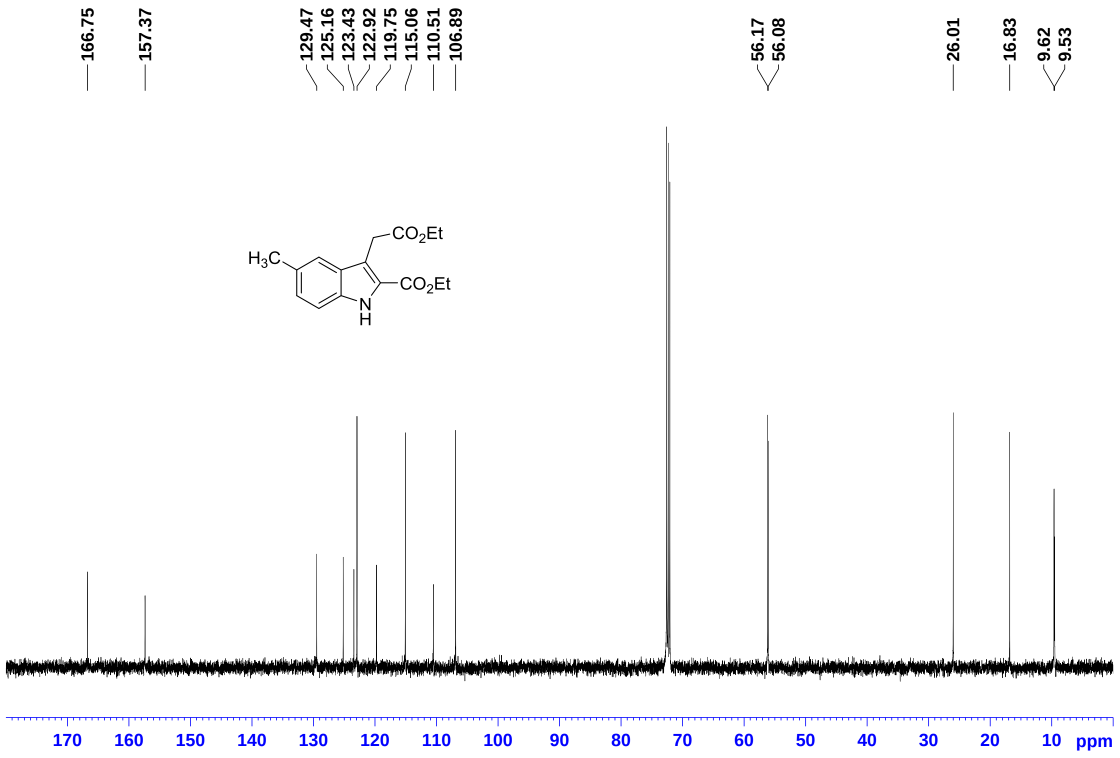

^{13}C NMR of Compound 1a

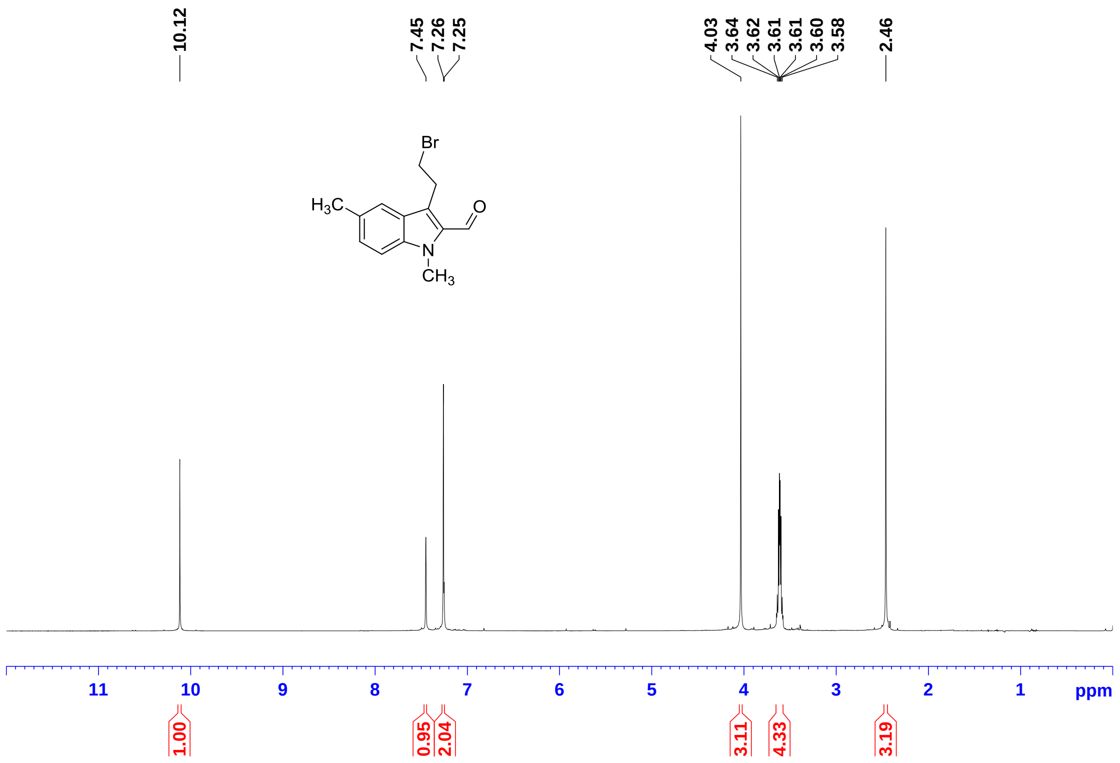

^{1}H NMR of Compound 5a

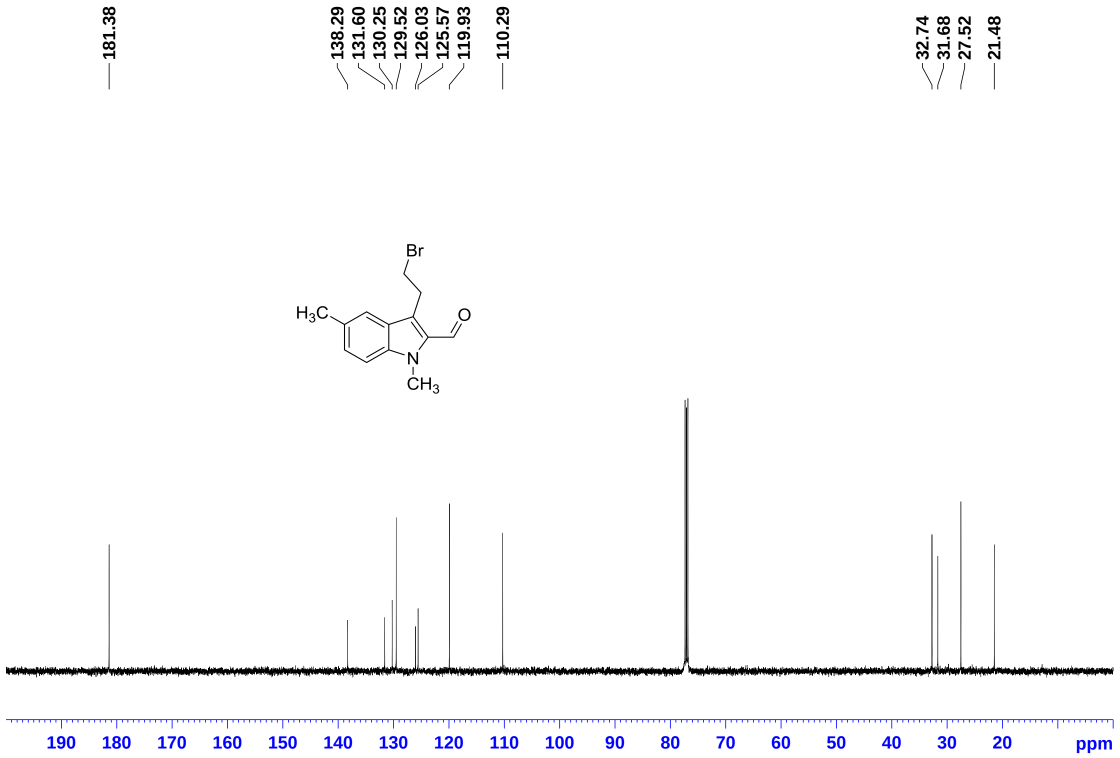

^{13}C NMR of Compound 5a

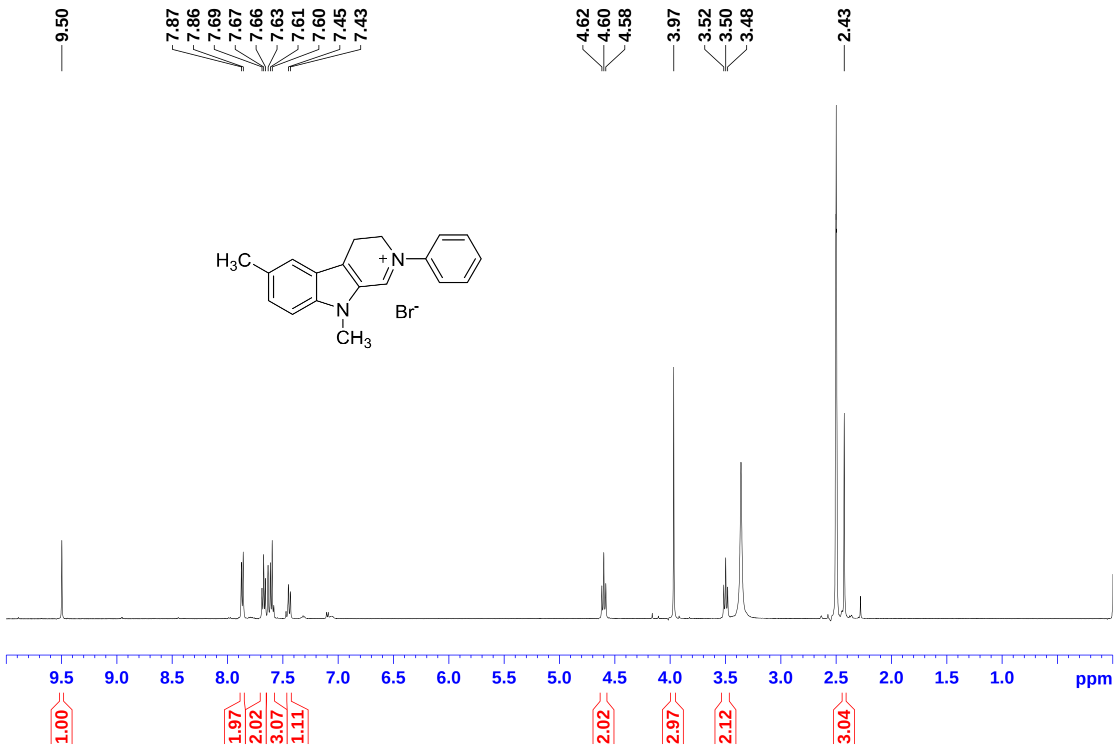

^{1}H NMR of Compound 6-1

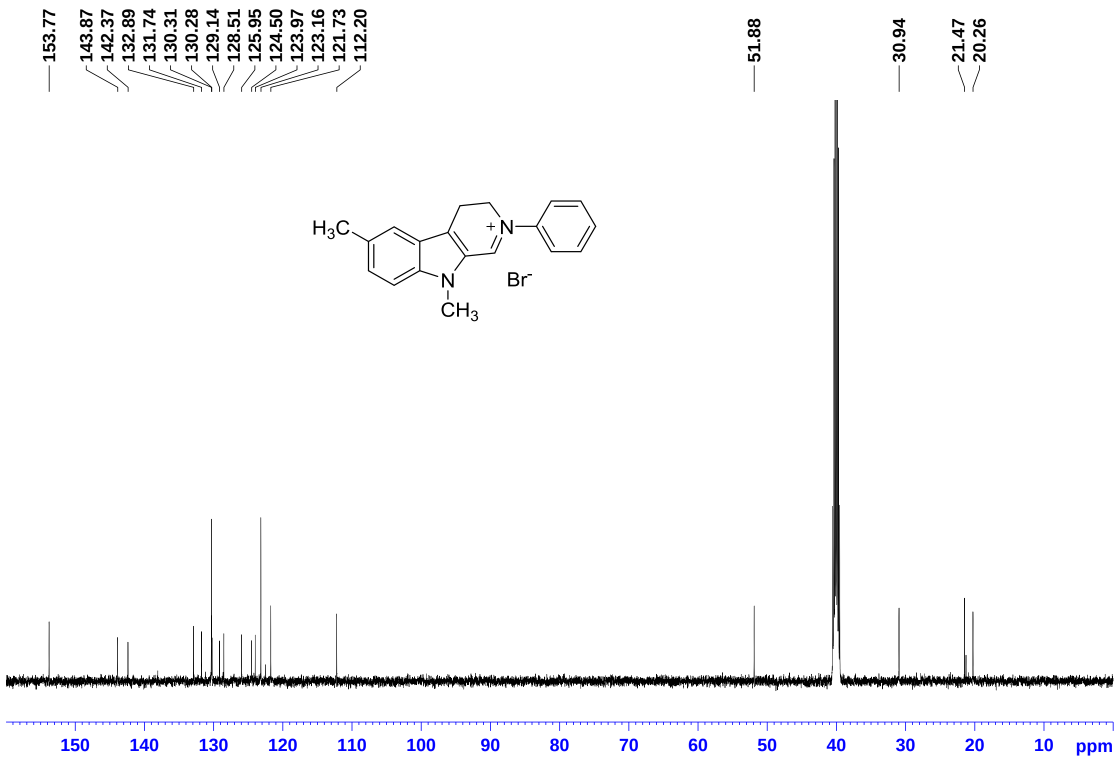

^{13}C NMR of Compound 6-1

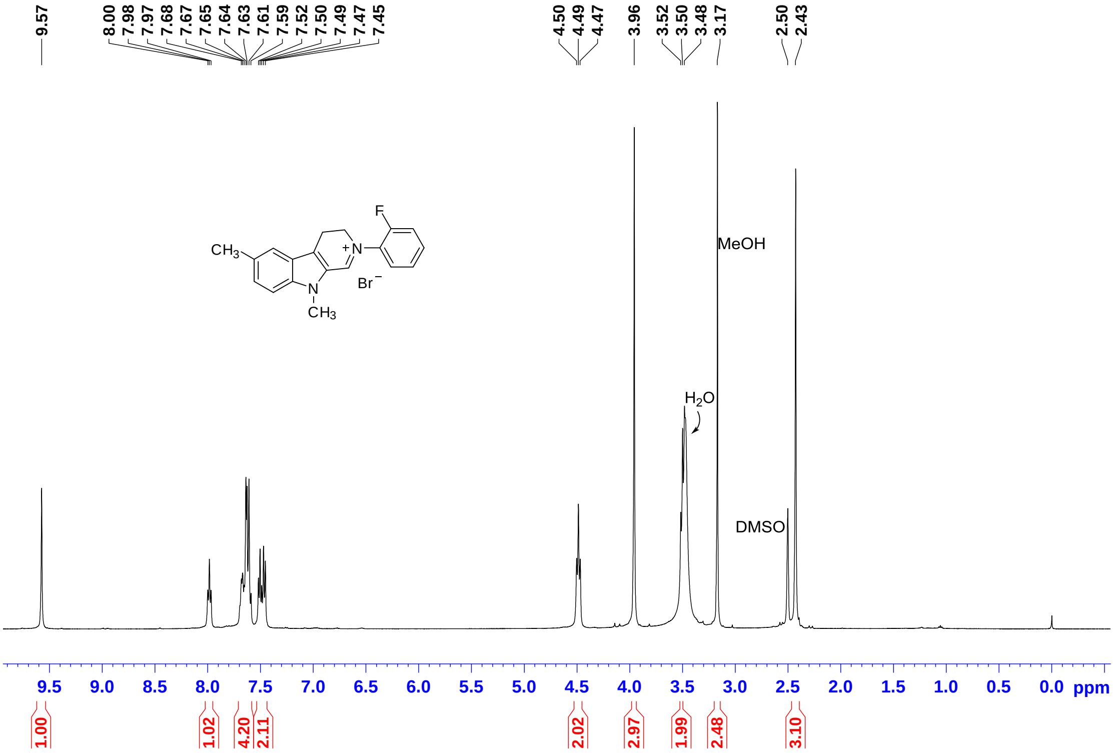

^{1}H NMR of Compound 6-2

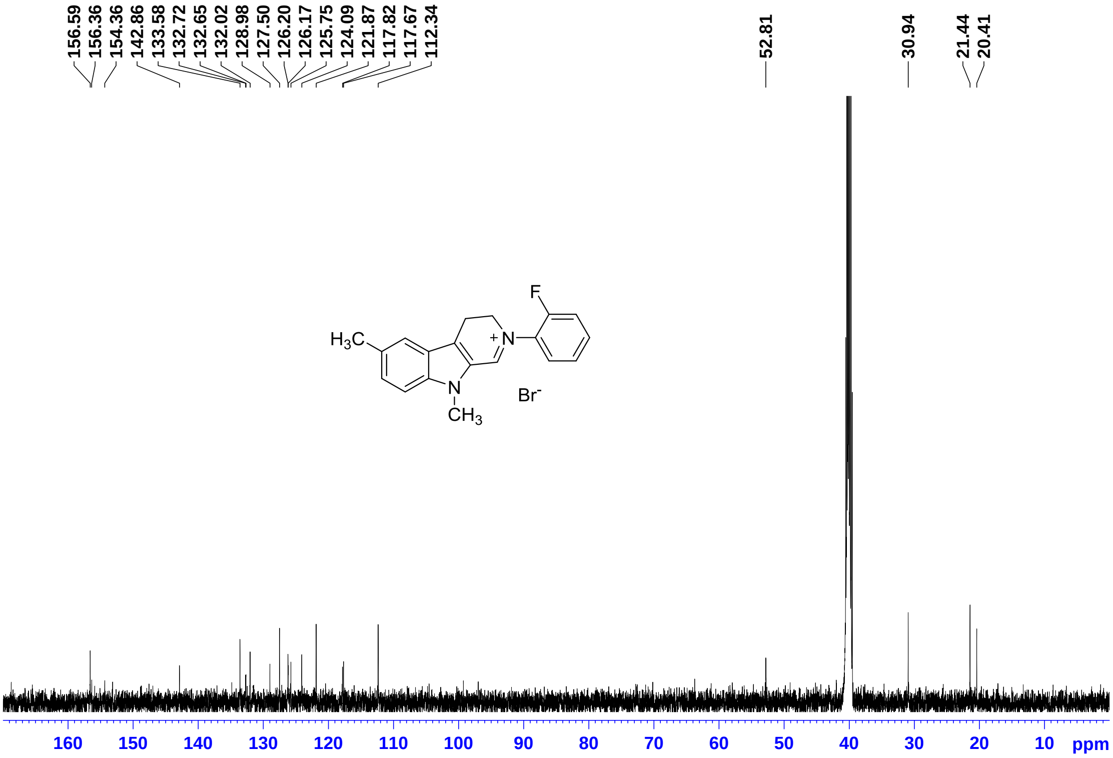

^{13}C NMR of Compound 6-2

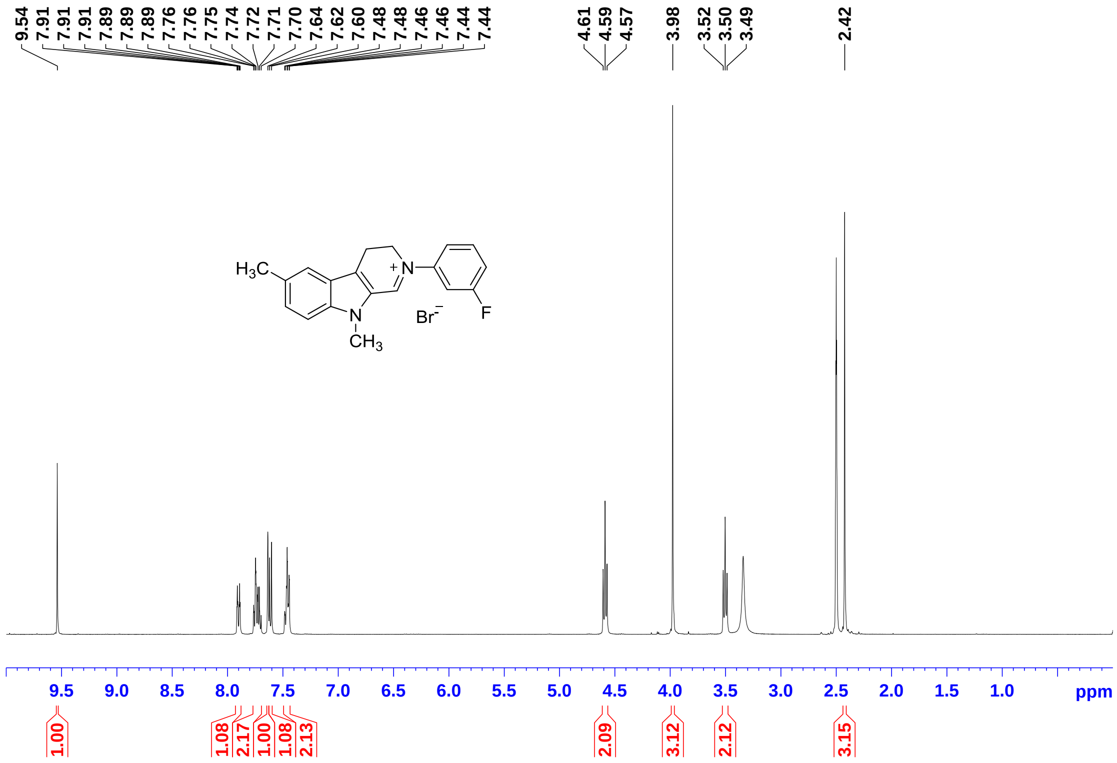

^{1}H NMR of Compound 6-3

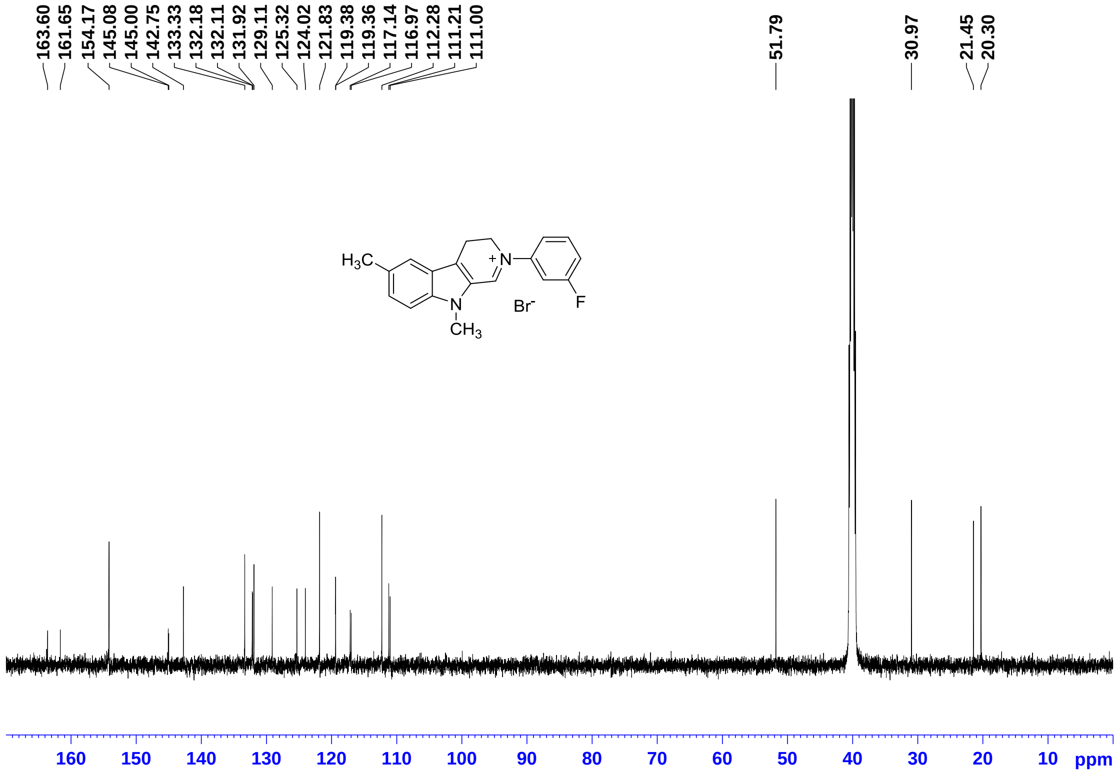

^{13}C NMR of Compound 6-3

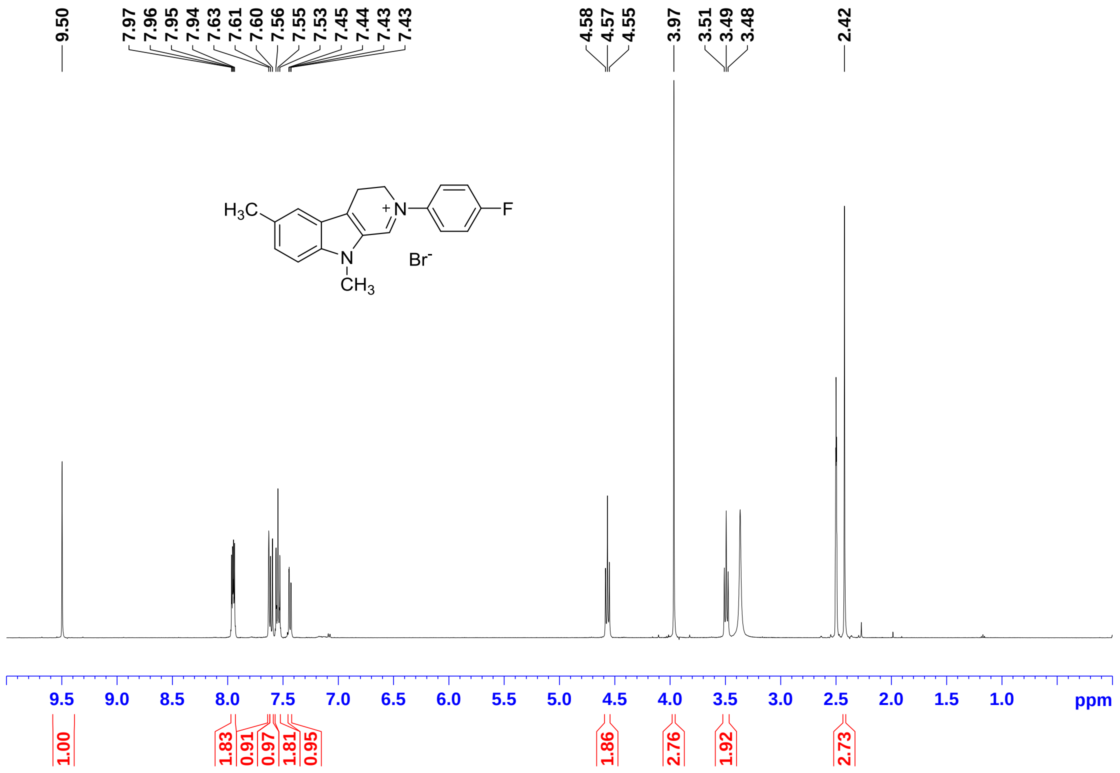

^{1}H NMR of Compound 6-4

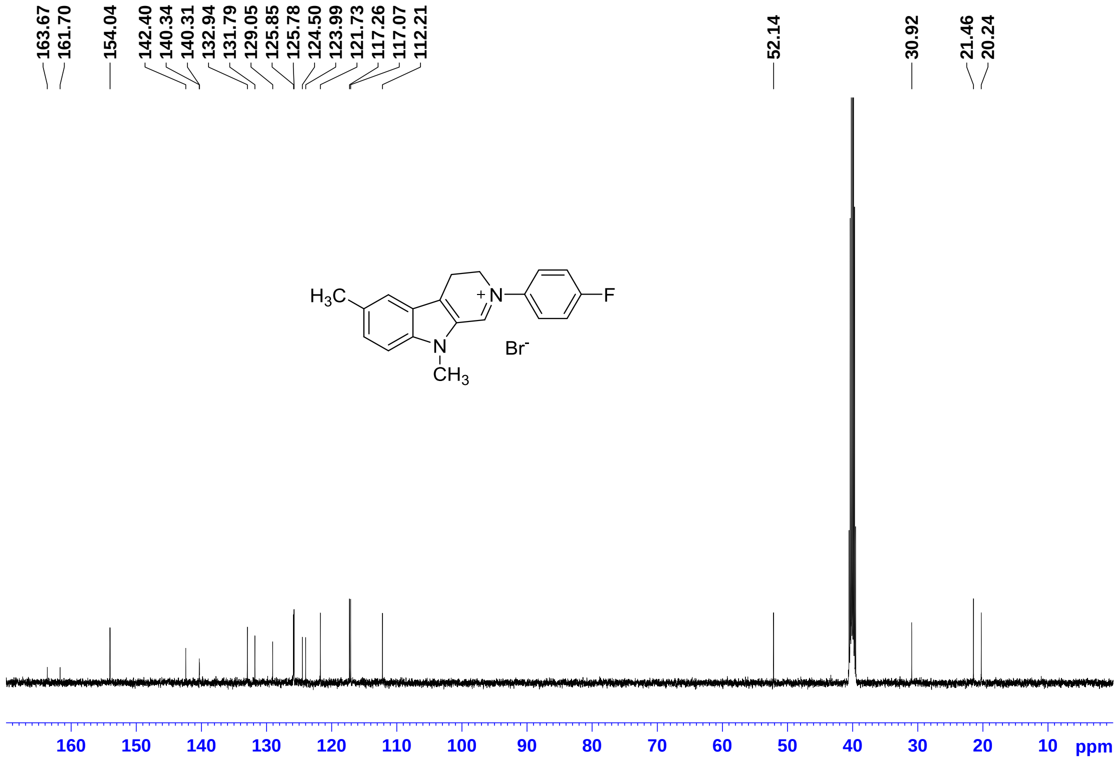

^{13}C NMR of Compound 6-4

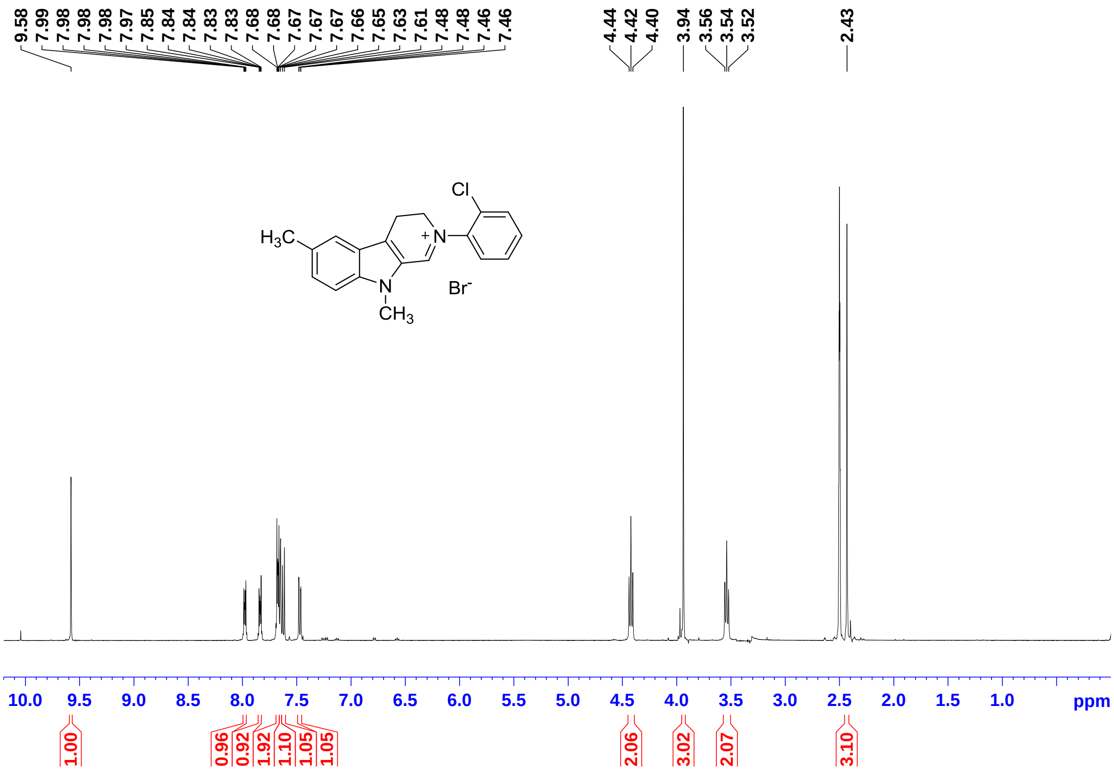

^{1}H NMR of Compound 6-5

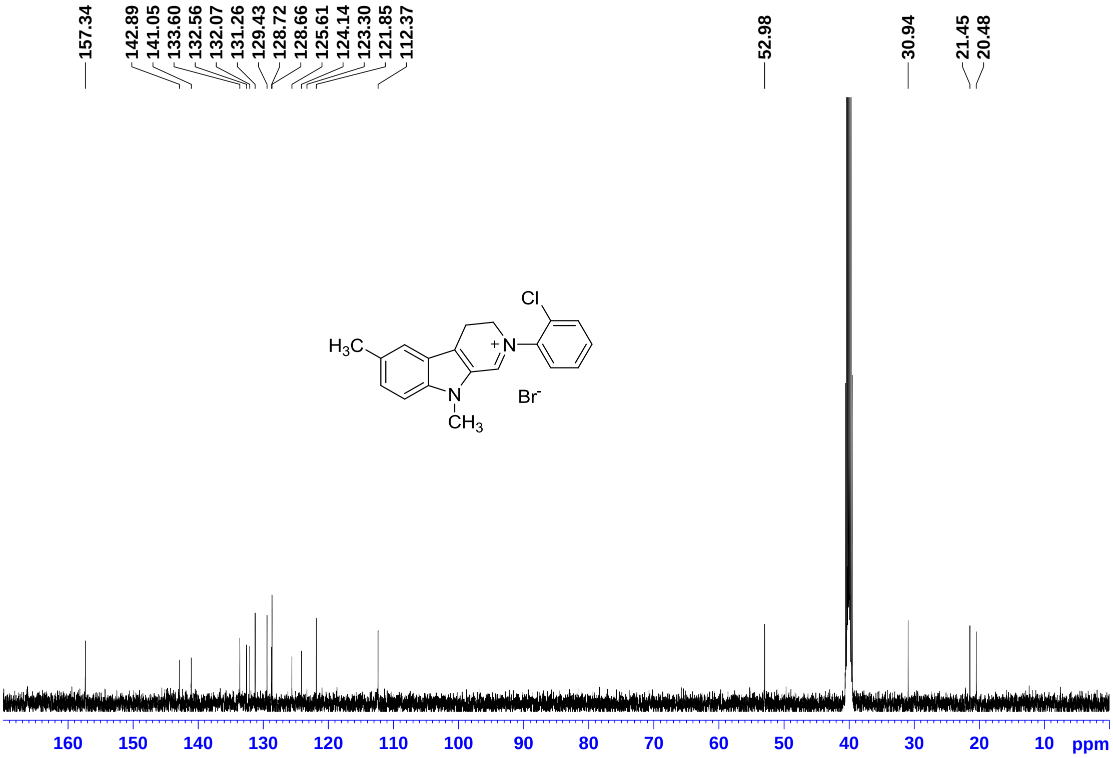

^{13}C NMR of Compound 6-5

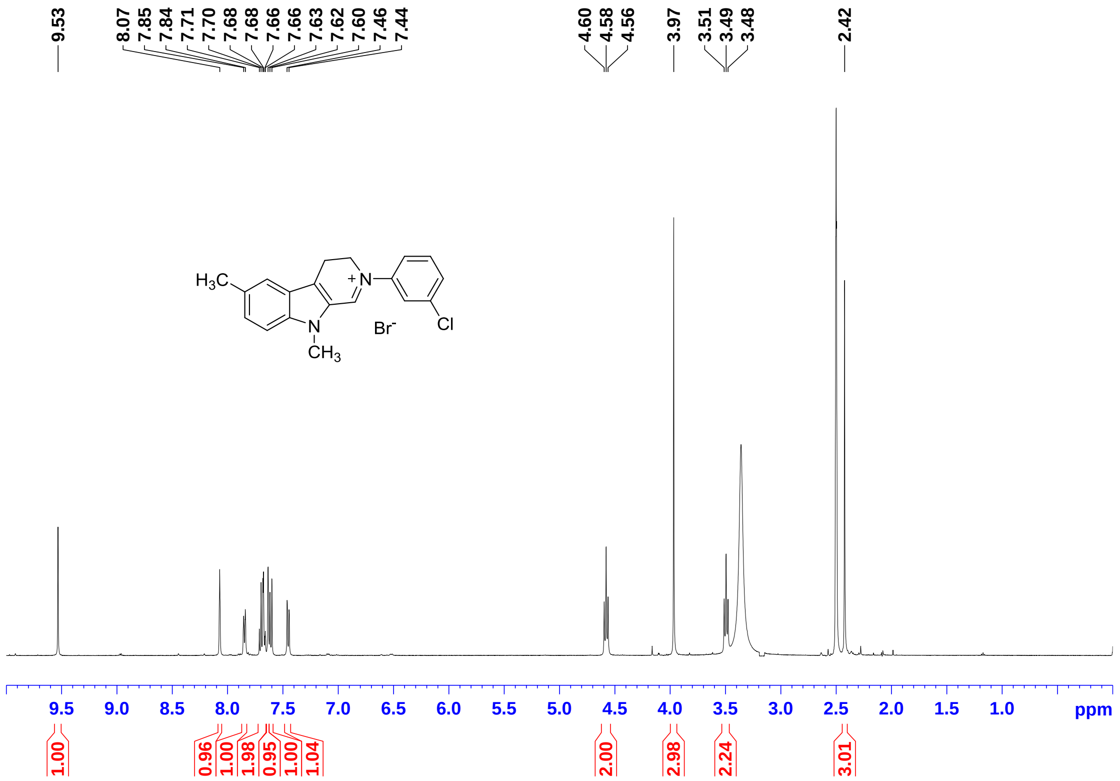

^{1}H NMR of Compound 6-6

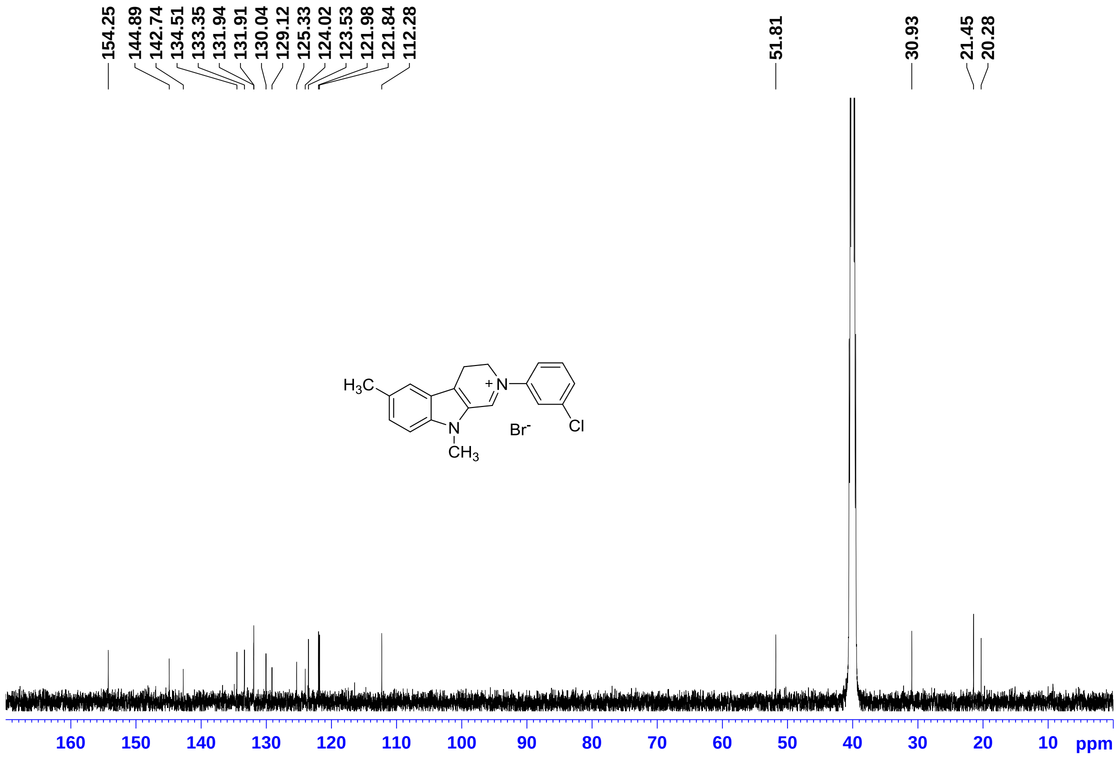

^{13}C NMR of Compound 6-6

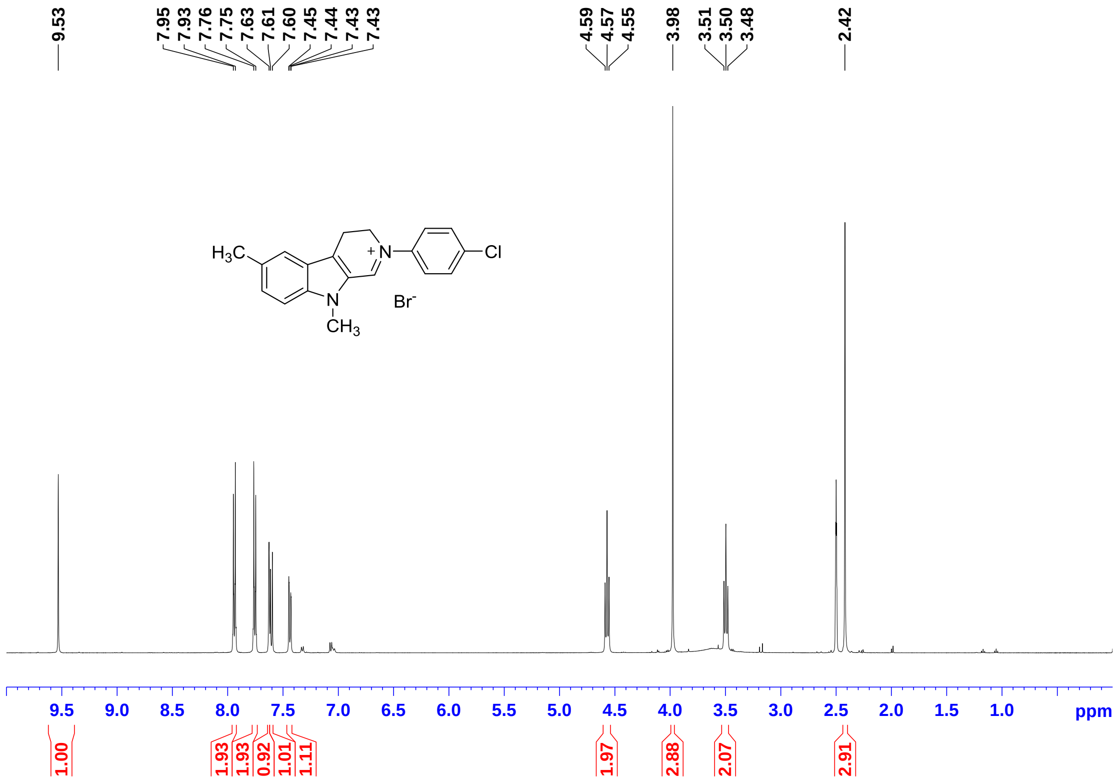

^{1}H NMR of Compound 6-7

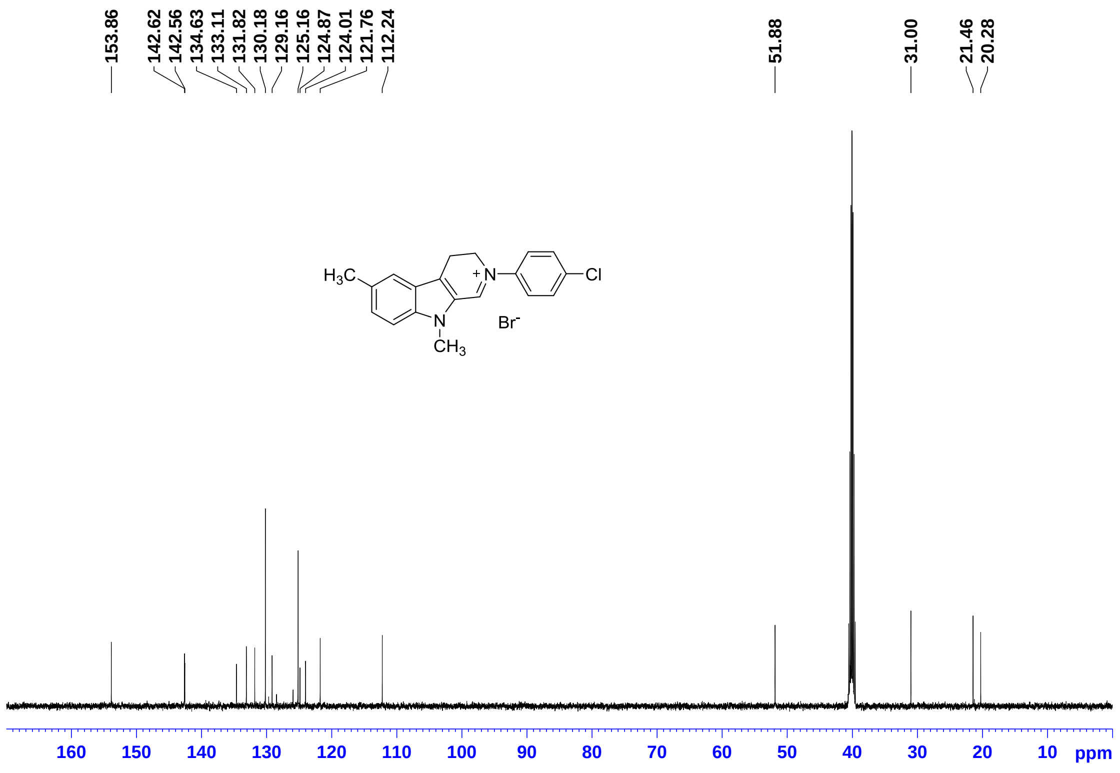

^{13}C NMR of Compound 6-7

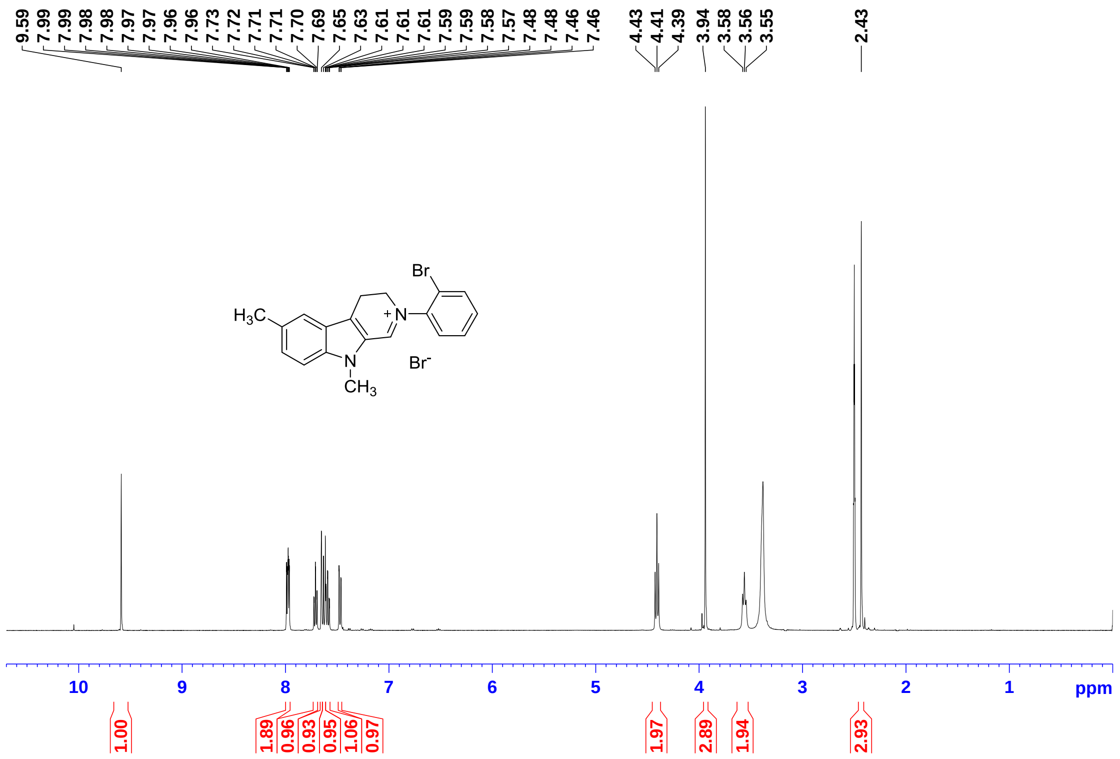

^{1}H NMR of Compound 6-8

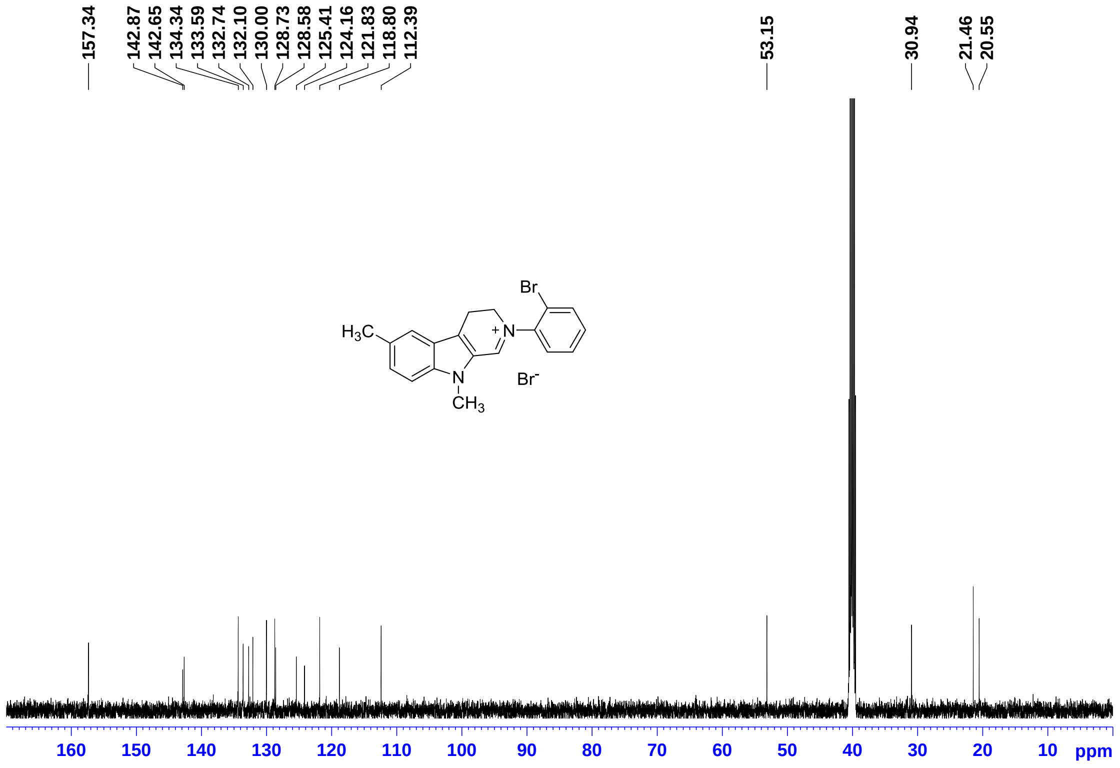

^{13}C NMR of Compound 6-8

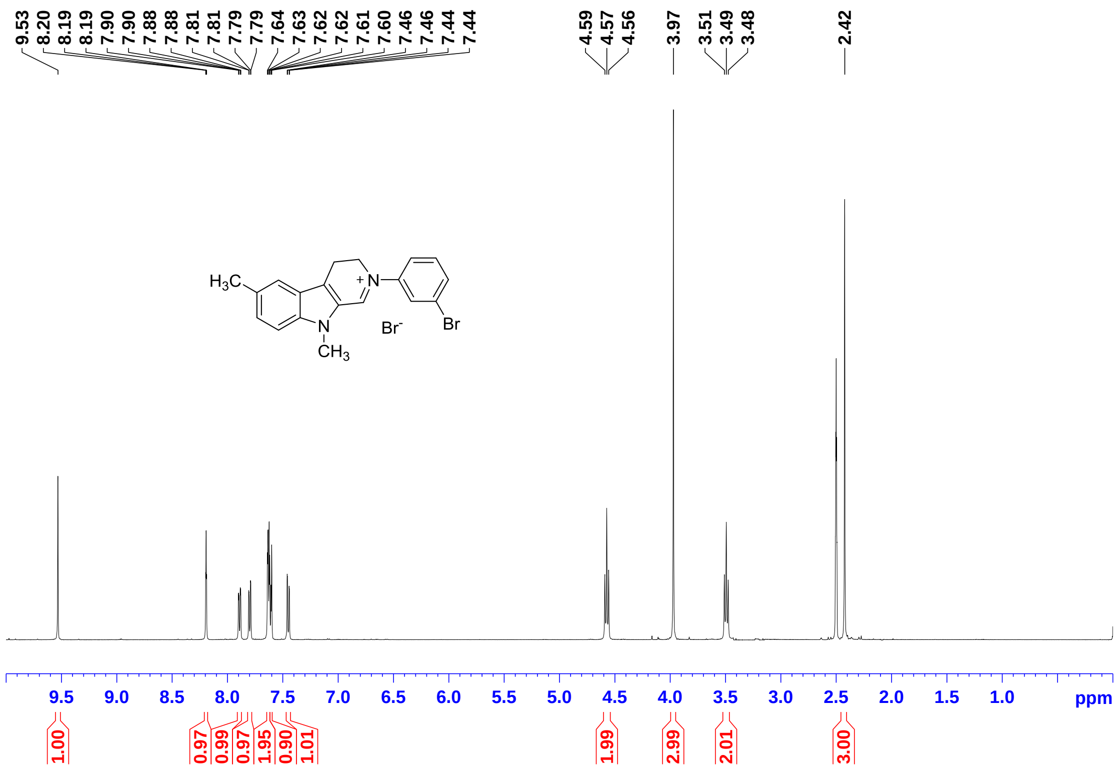

^{1}H NMR of Compound 6-9

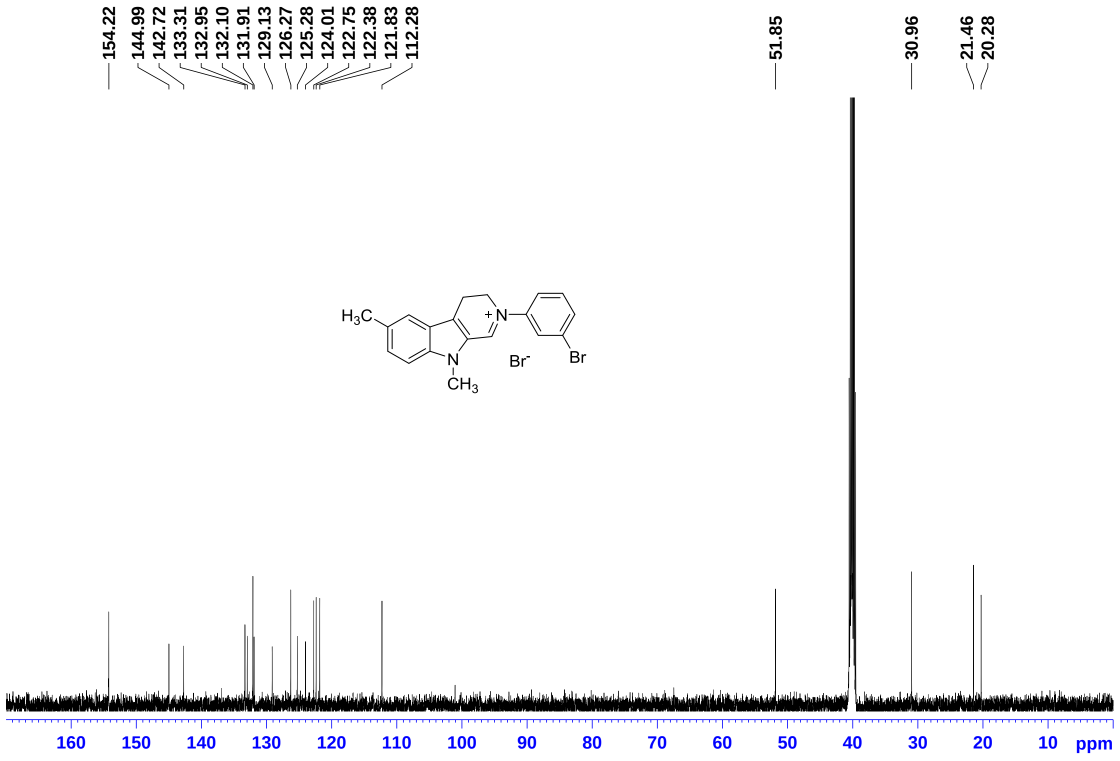

^{13}C NMR of Compound 6-9

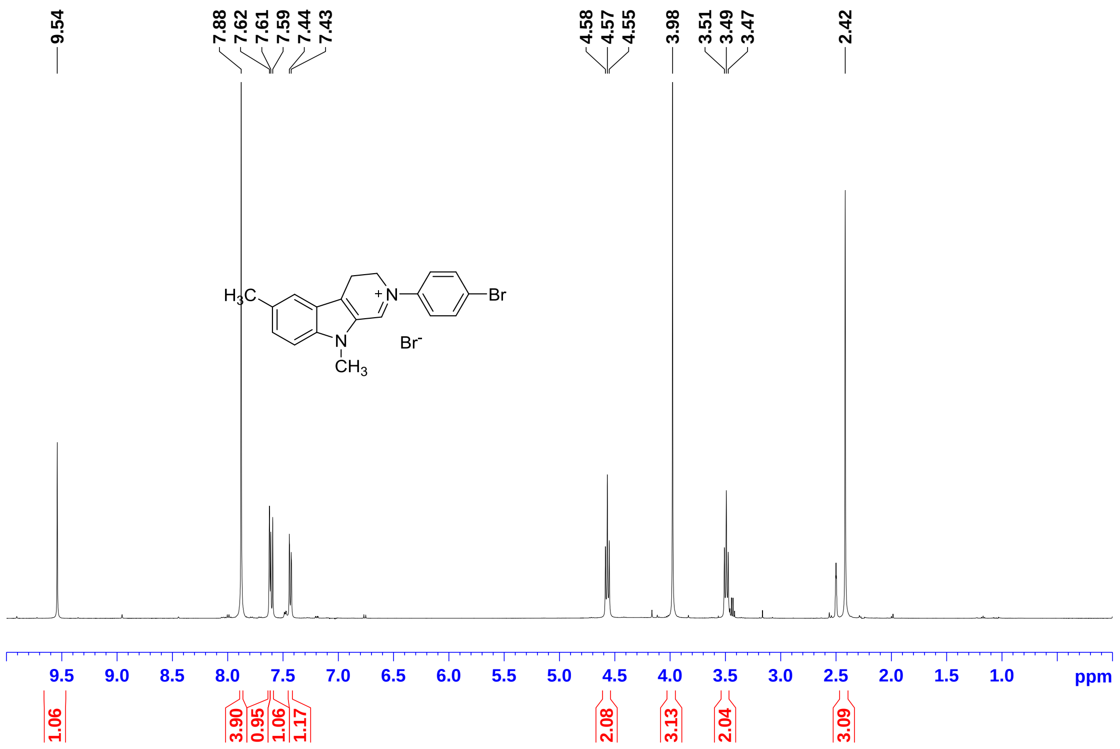

^{1}H NMR of Compound 6-10

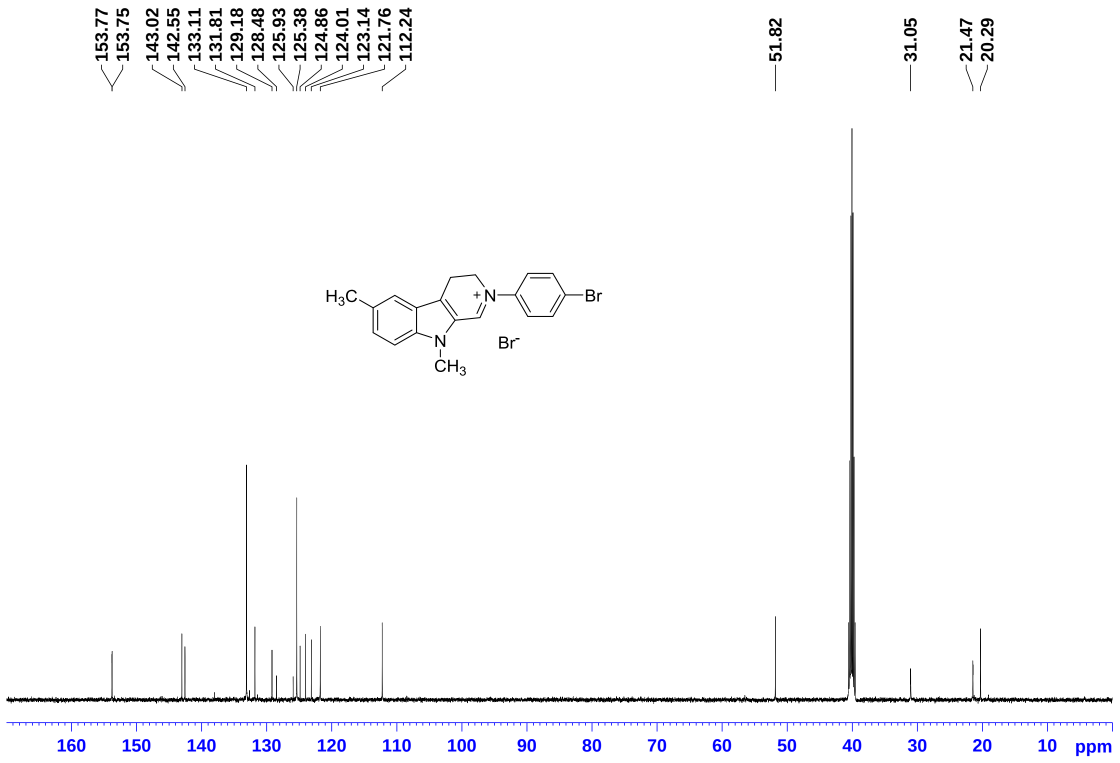

^{13}C NMR of Compound 6-10

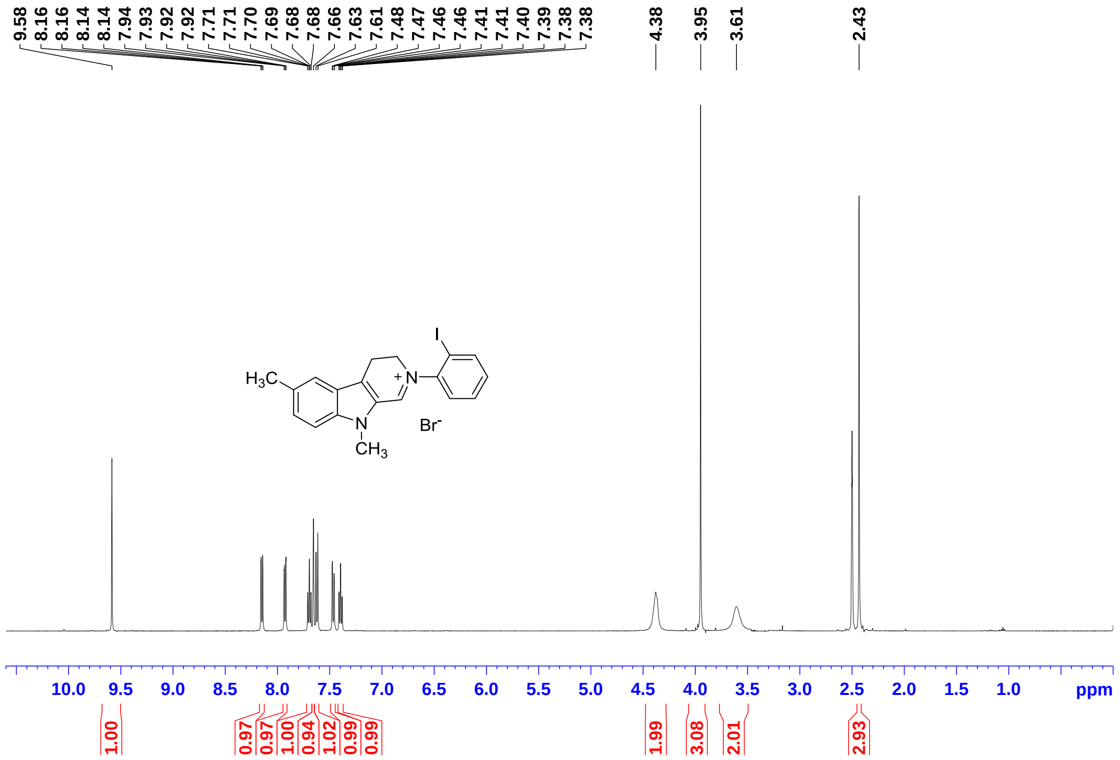

^{1}H NMR of Compound 6-11

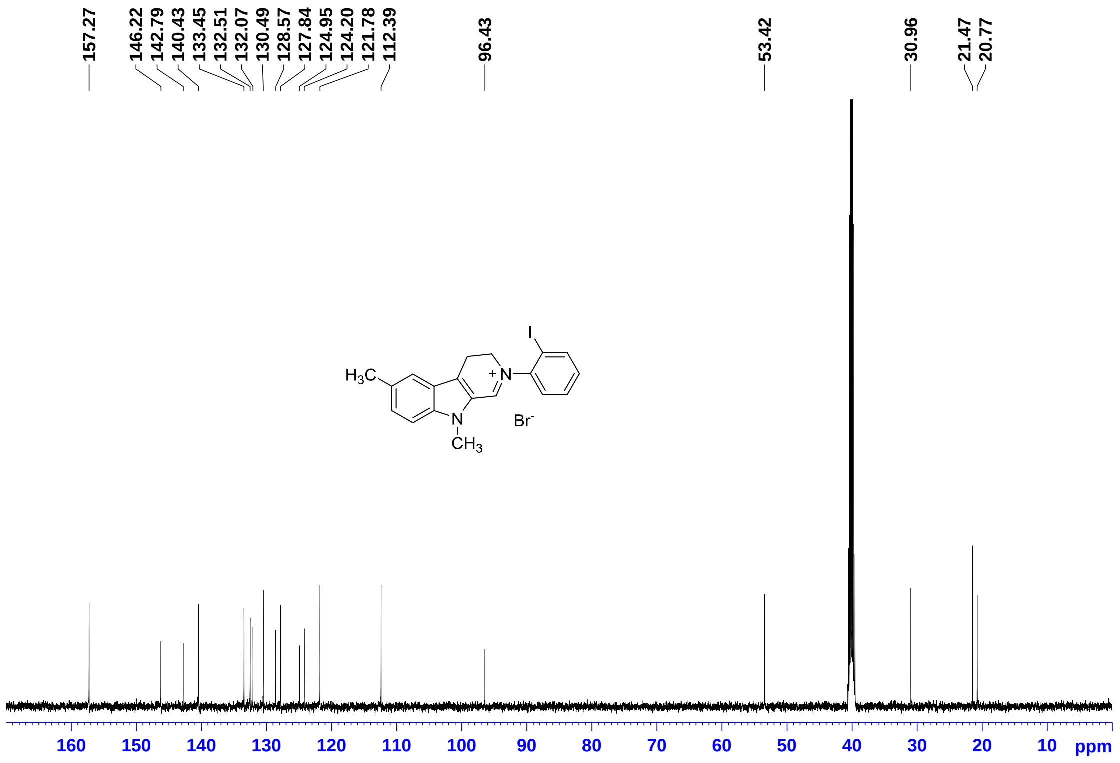

^{13}C NMR of Compound 6-11

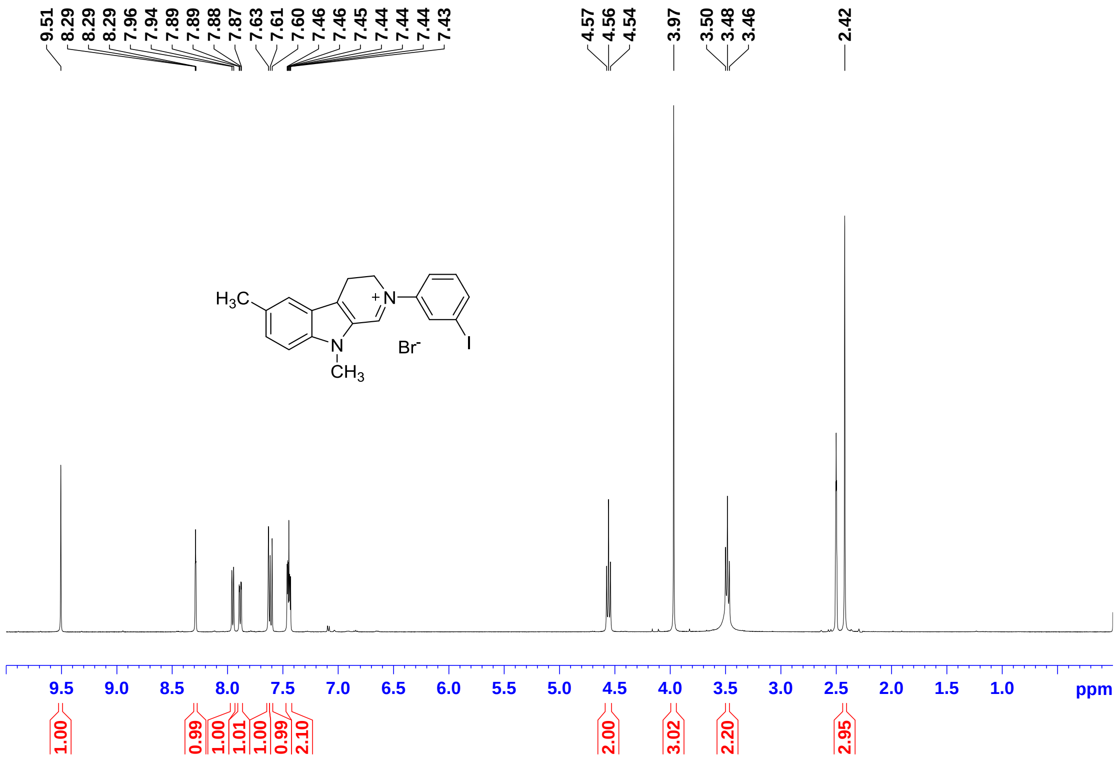

^{1}H NMR of Compound 6-12

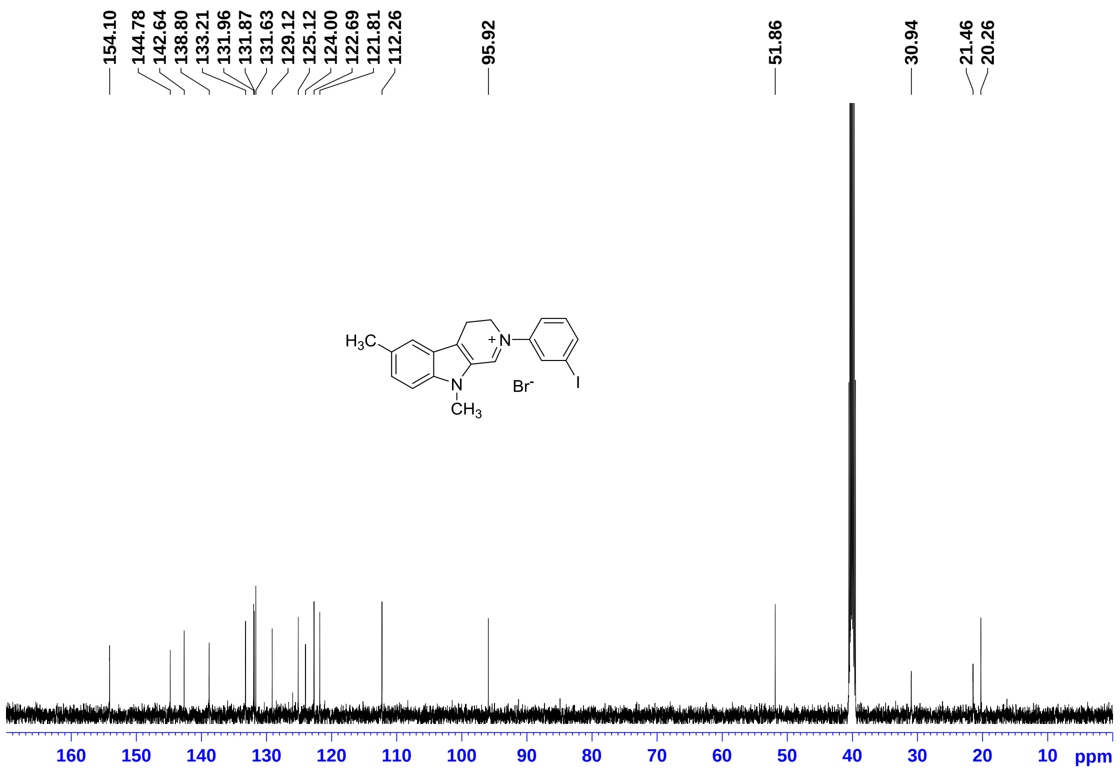

^{13}C NMR of Compound 6-12

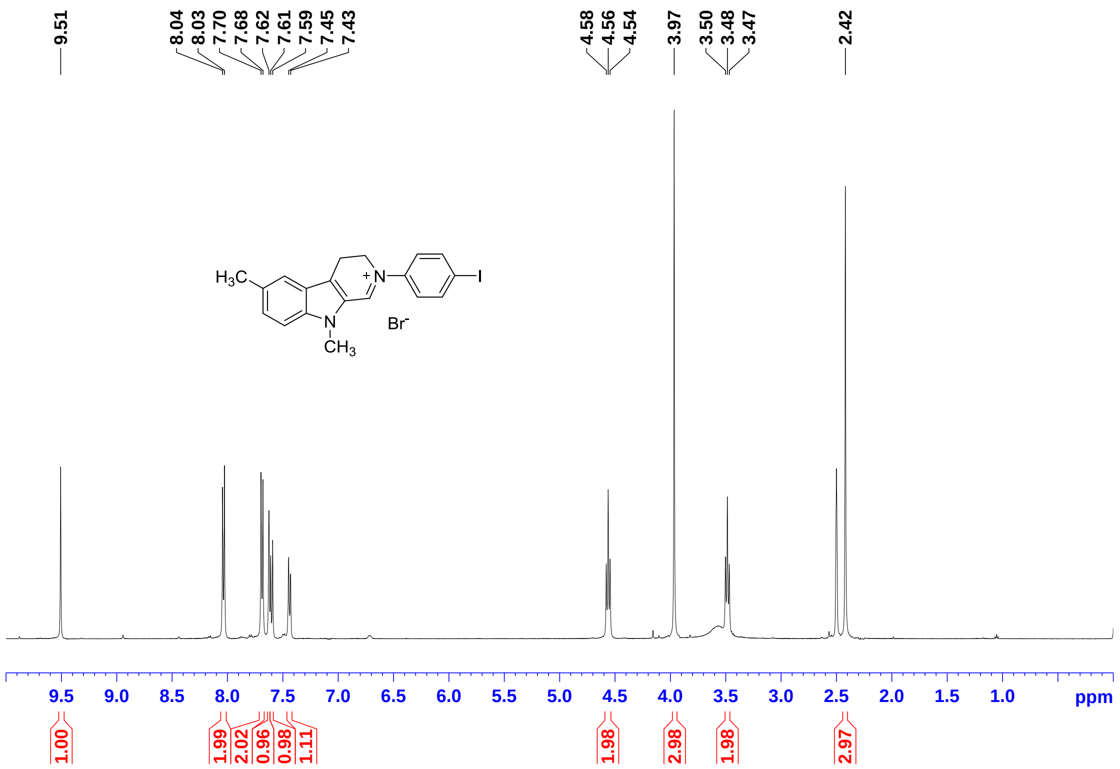

^{1}H NMR of Compound 6-13

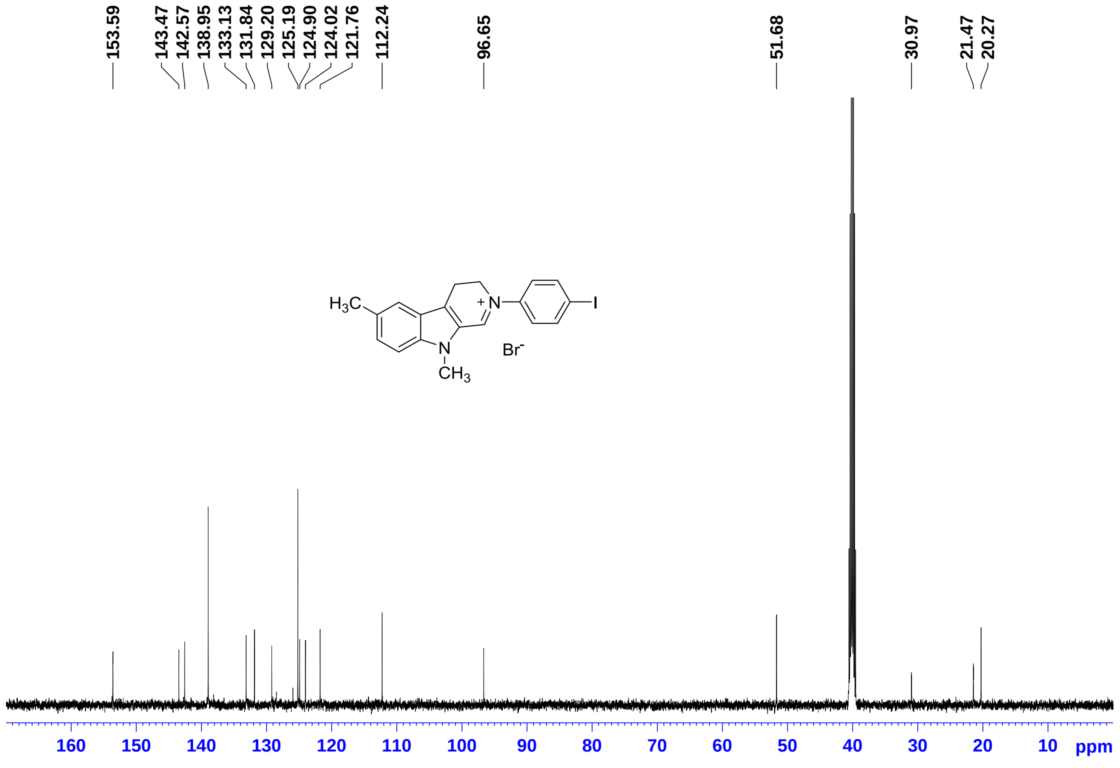

^{13}C NMR of Compound 6-13

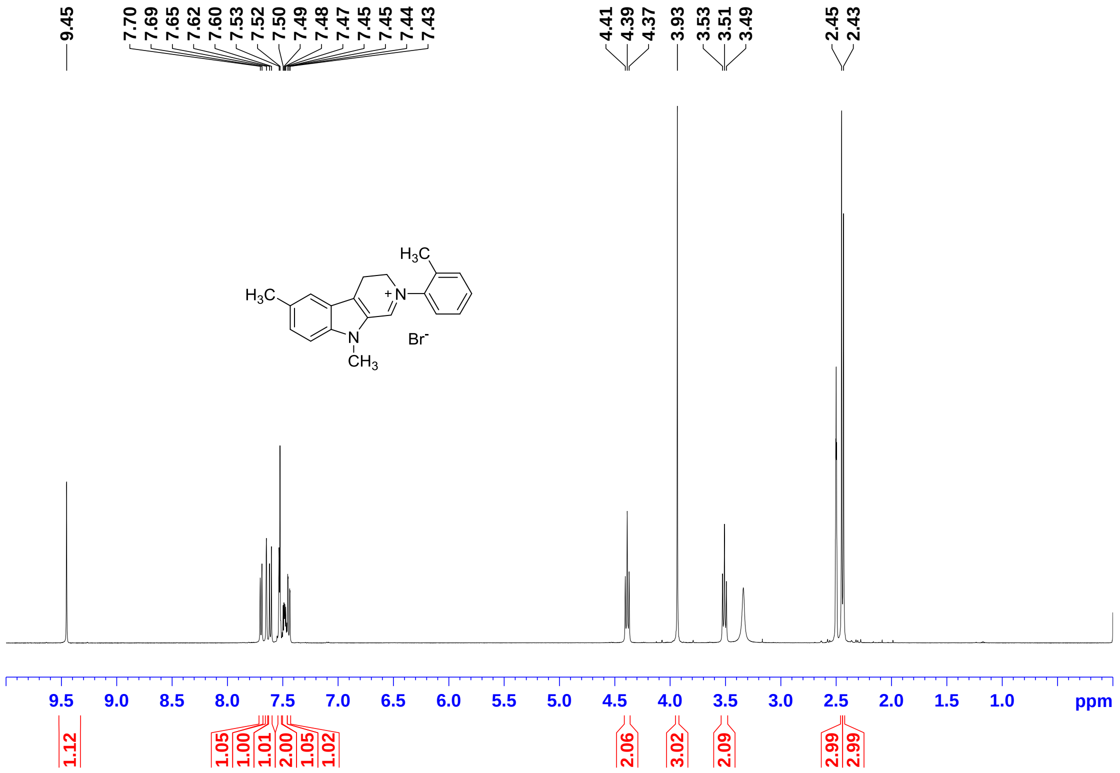

^{1}H NMR of Compound 6-14

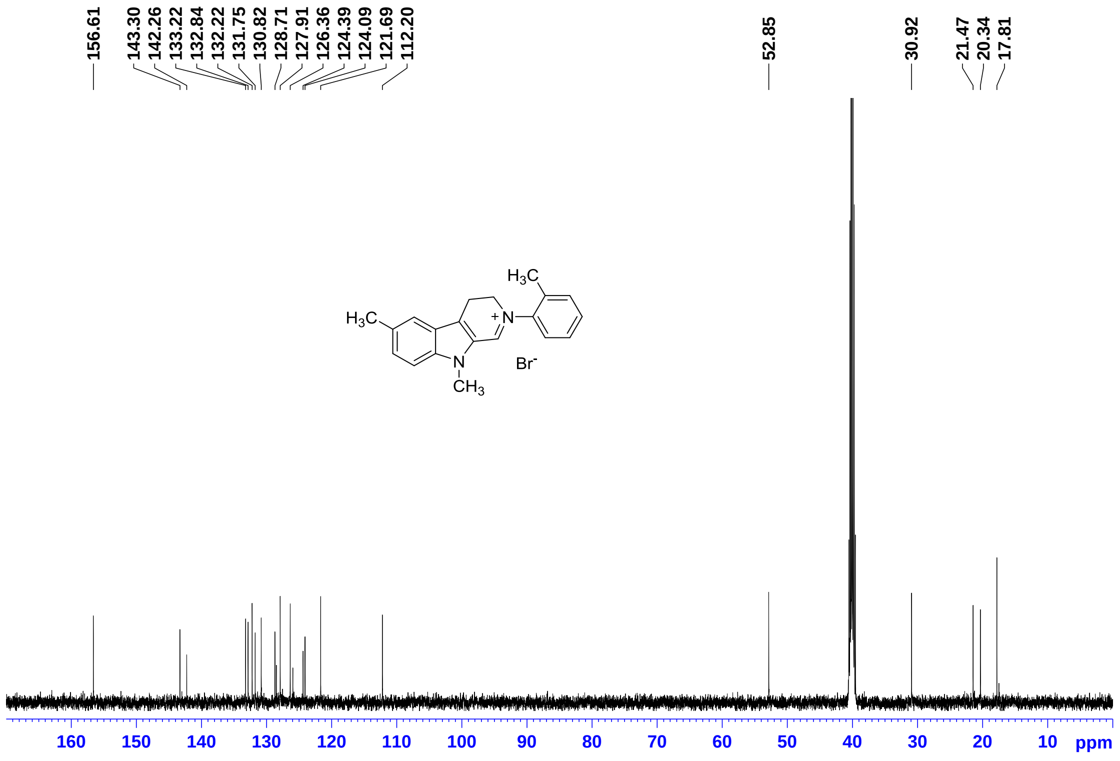

^{13}C NMR of Compound 6-14

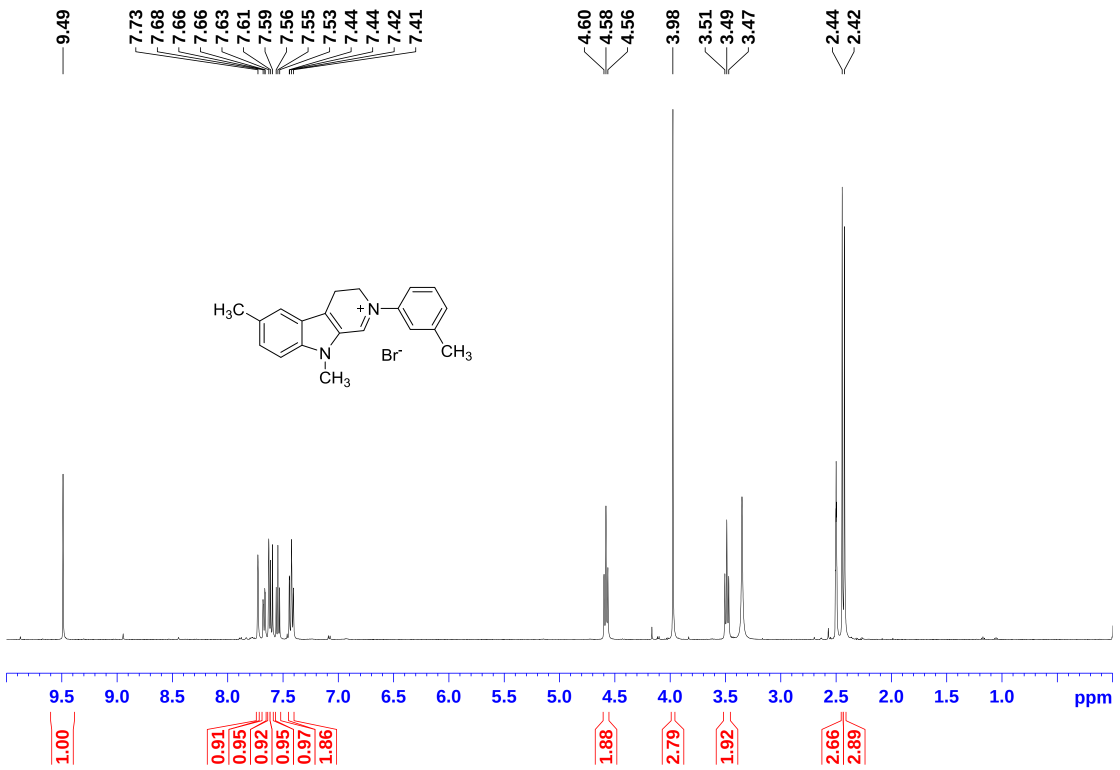

^{1}H NMR of Compound 6-15

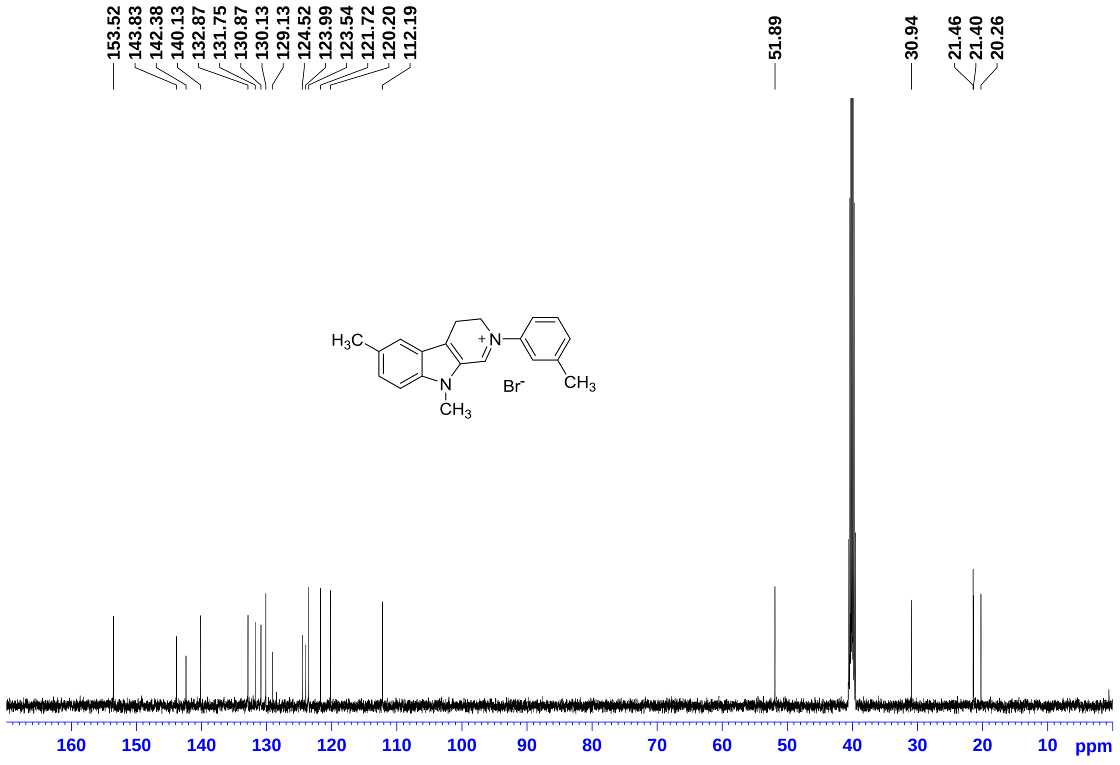

^{13}C NMR of Compound 6-15

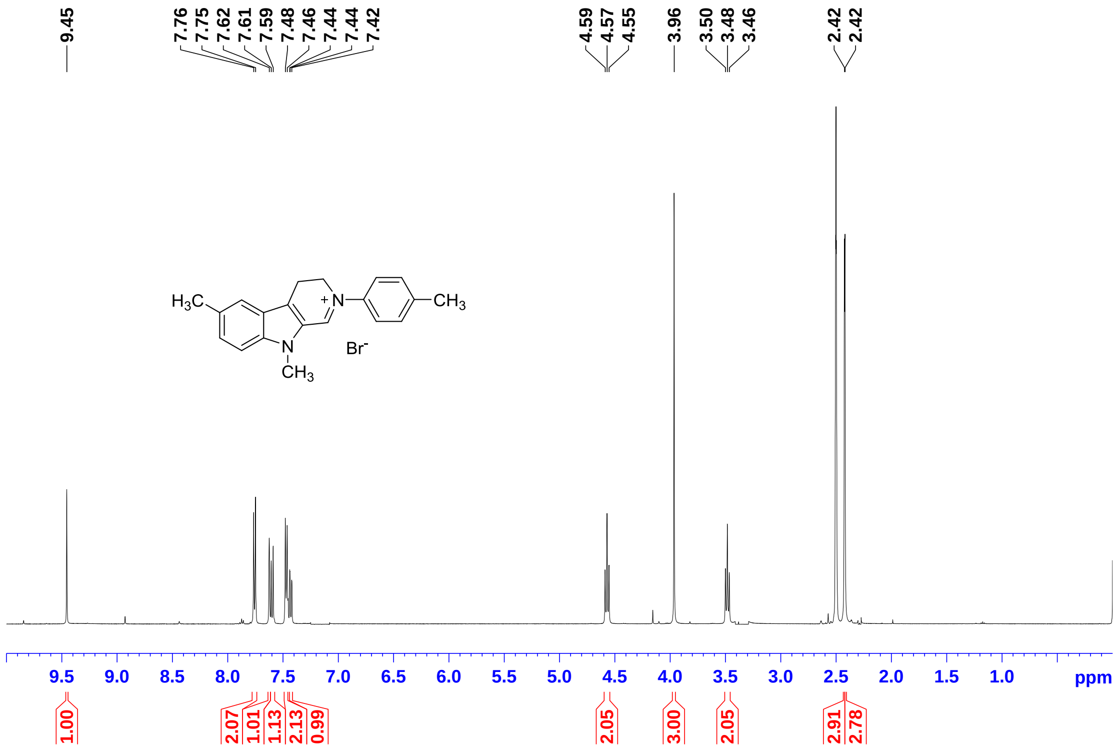

^{1}H NMR of Compound 6-16

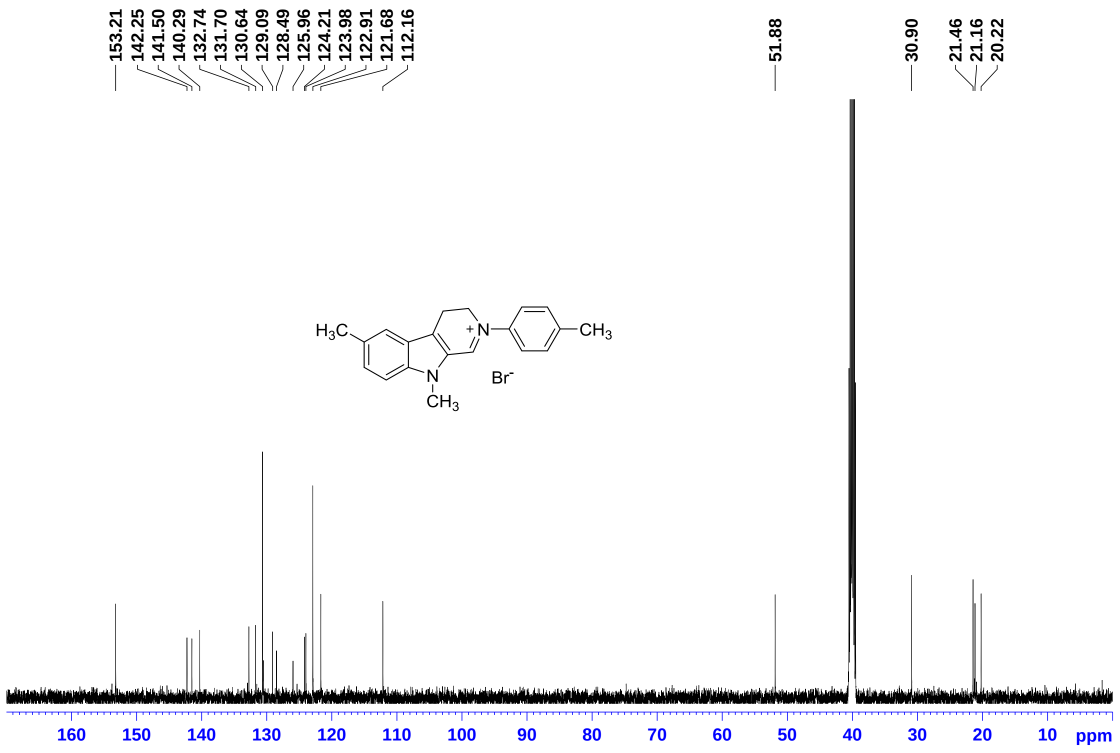

^{13}C NMR of Compound 6-16

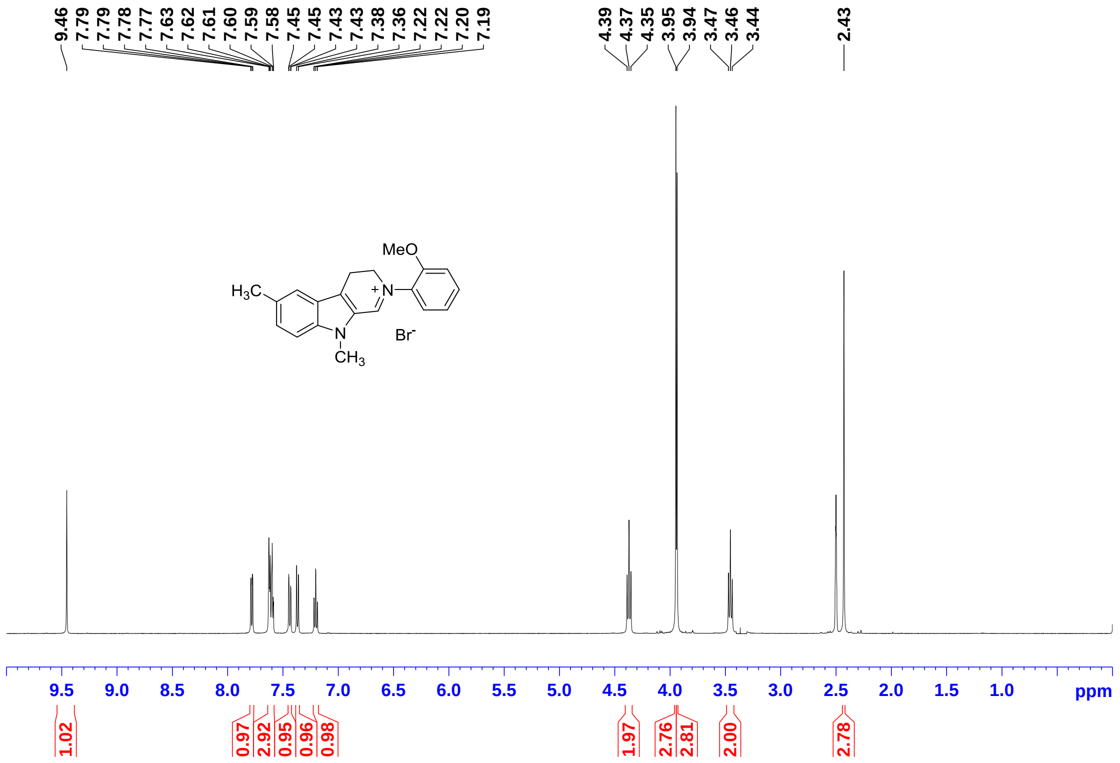

^{1}H NMR of Compound 6-17

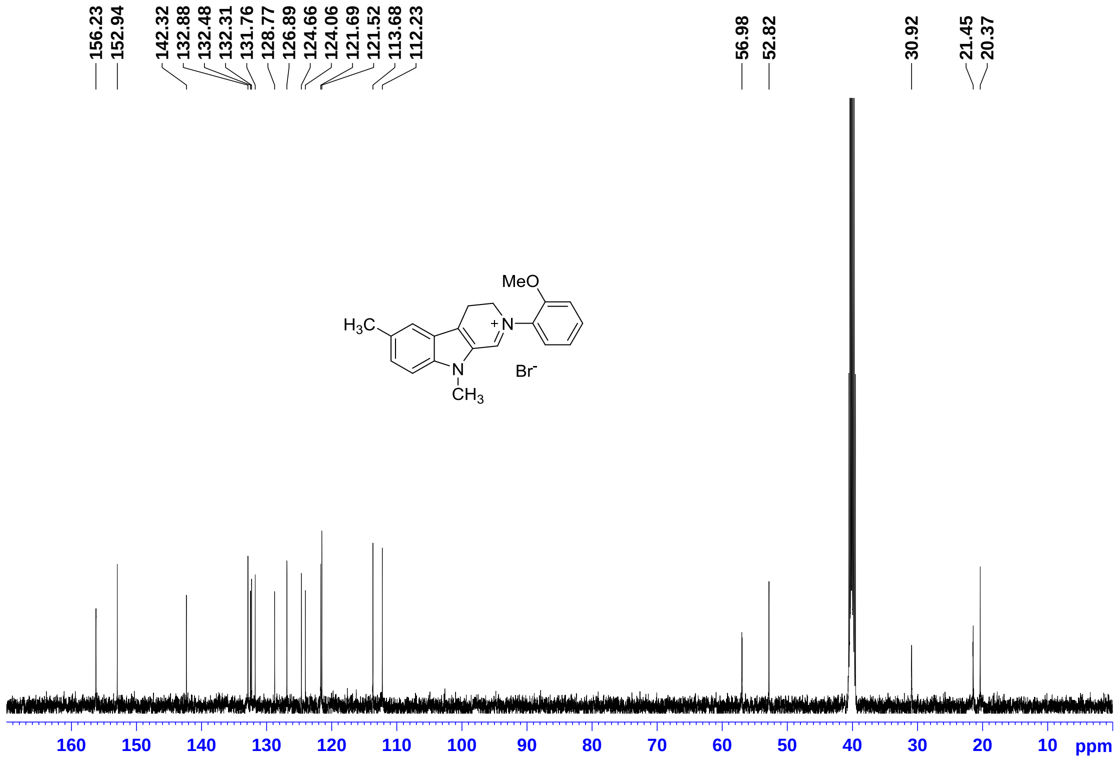

^{13}C NMR of Compound 6-17

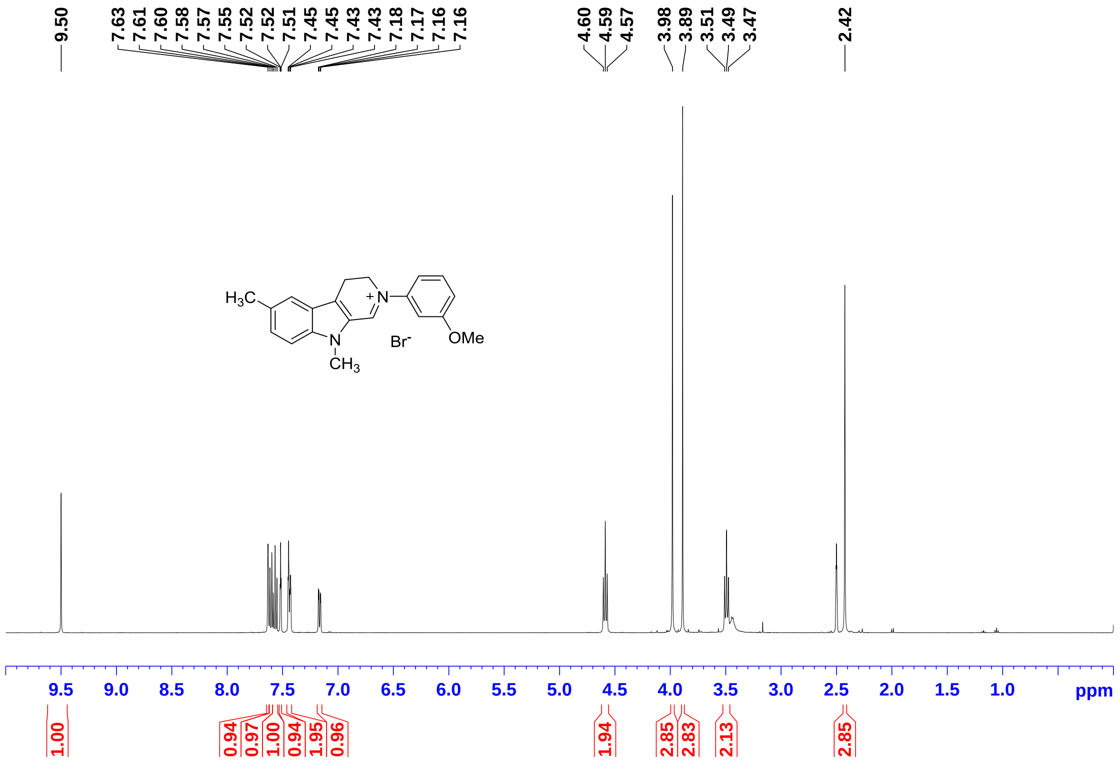

^{1}H NMR of Compound 6-18

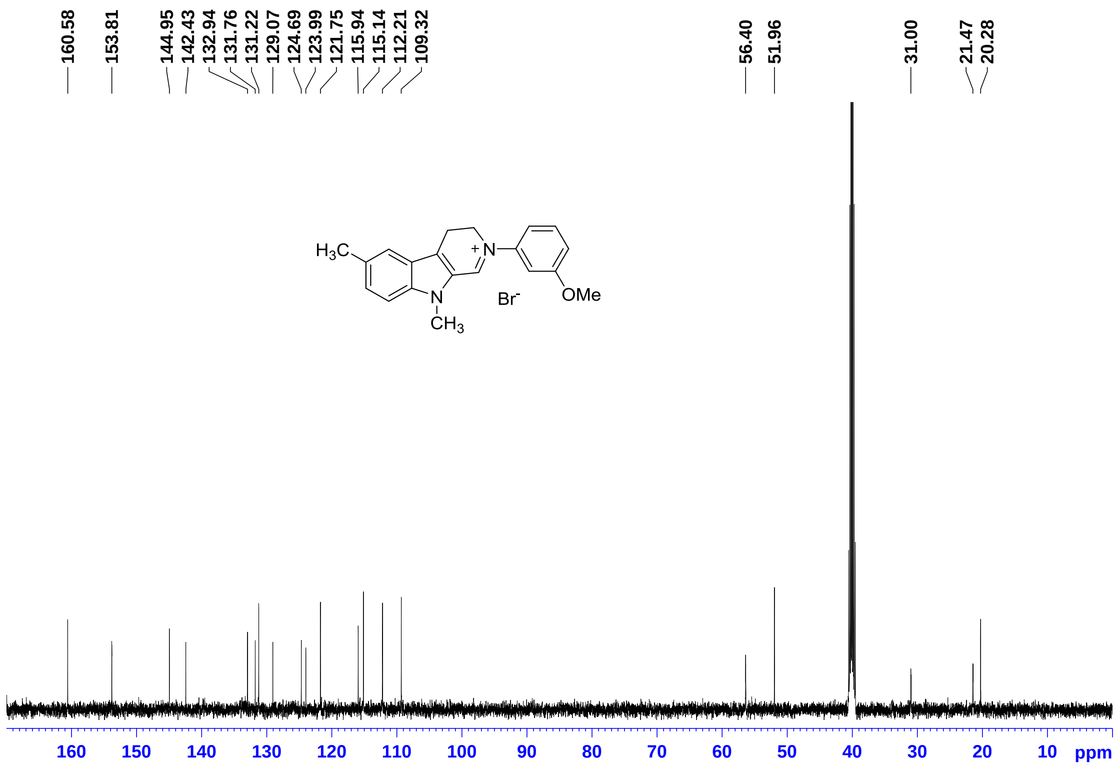

^{13}C NMR of Compound 6-18

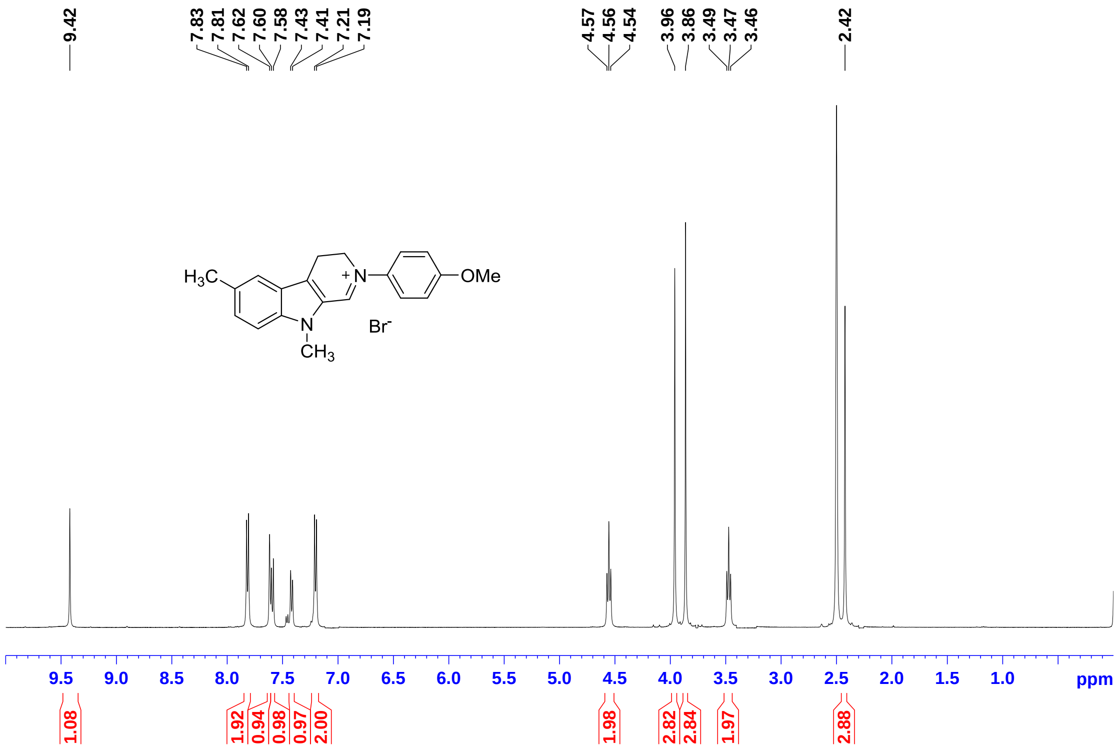

^{1}H NMR of Compound 6-19

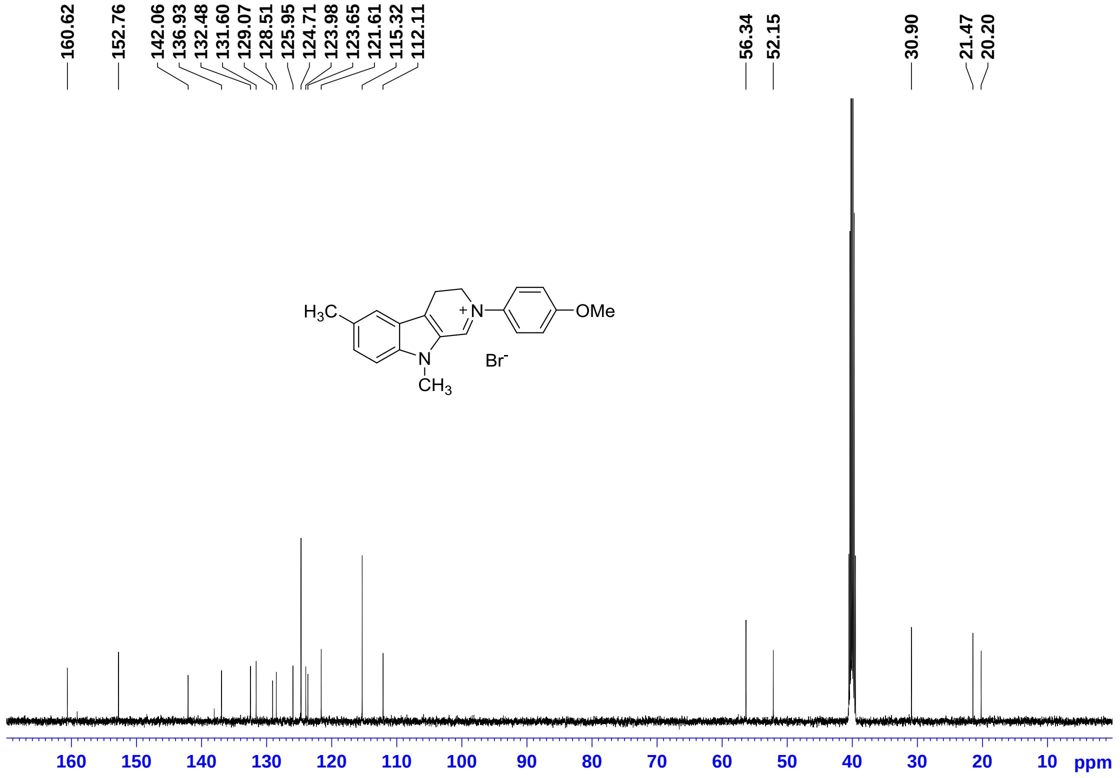

^{13}C NMR of Compound 6-19

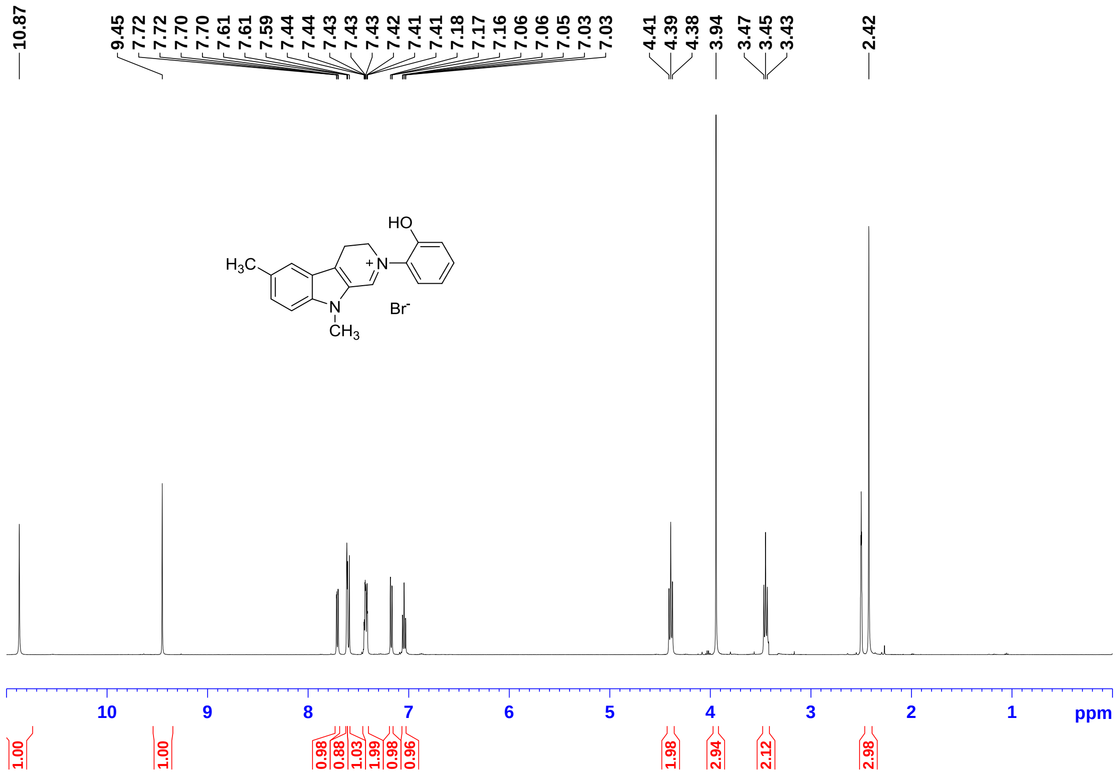

^{1}H NMR of Compound 6-20

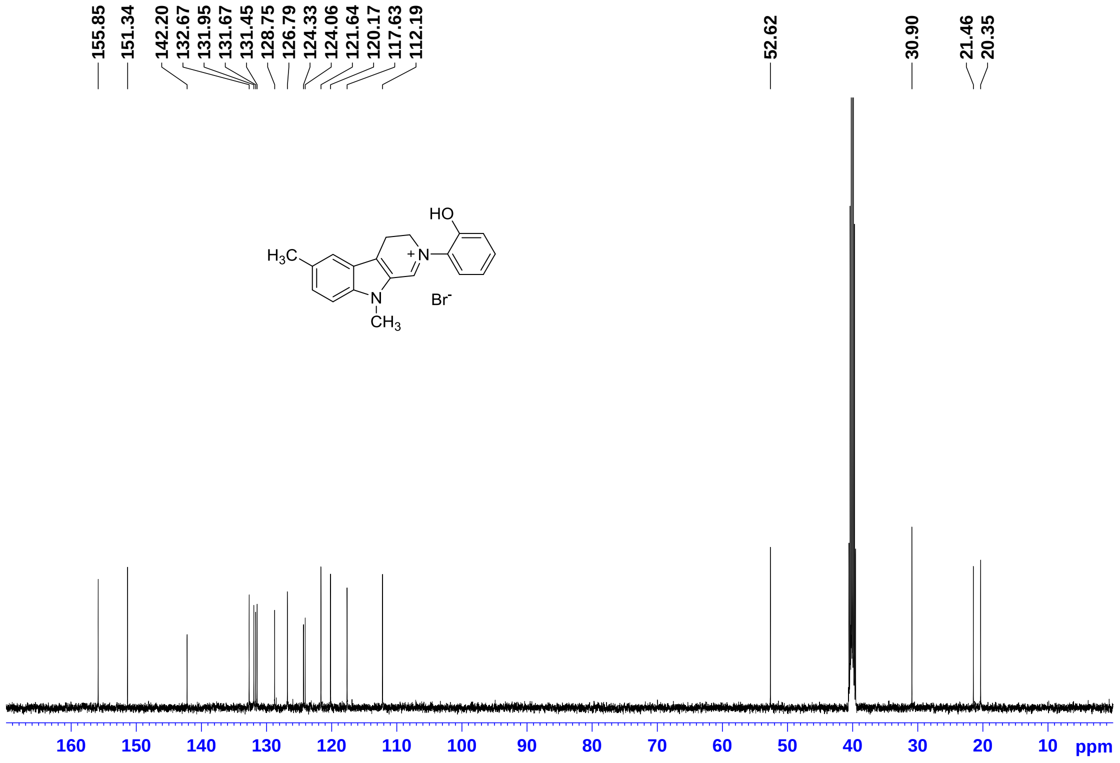

^{13}C NMR of Compound 6-20

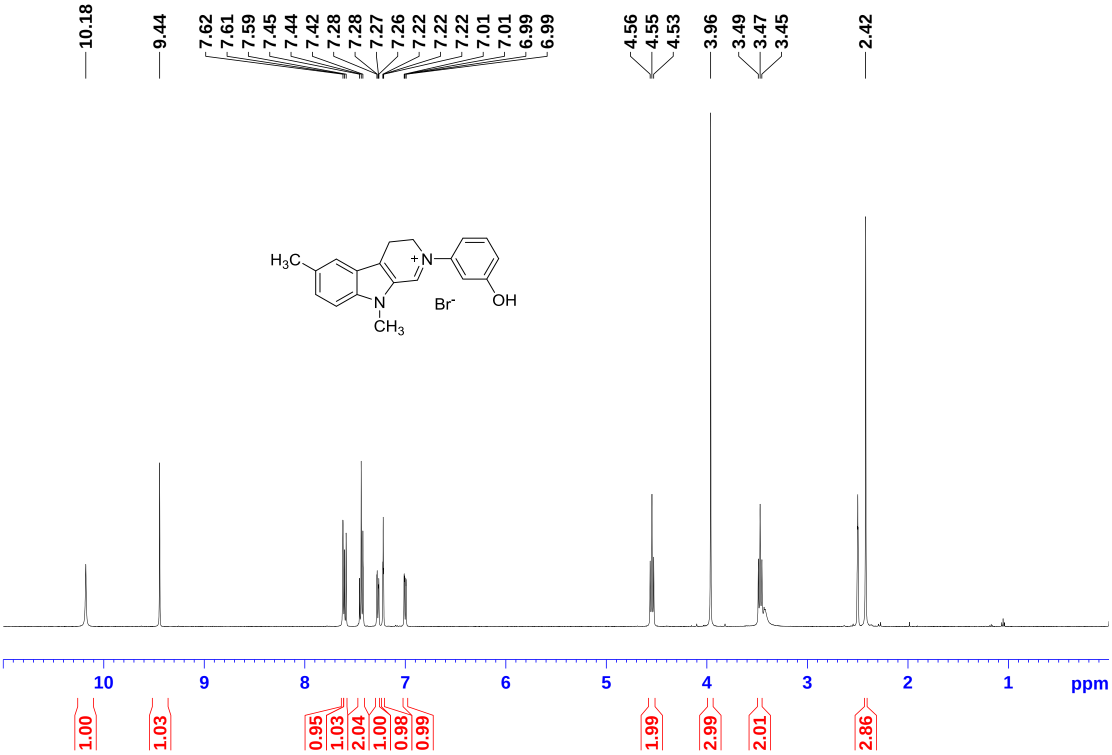

^{1}H NMR of Compound 6-21

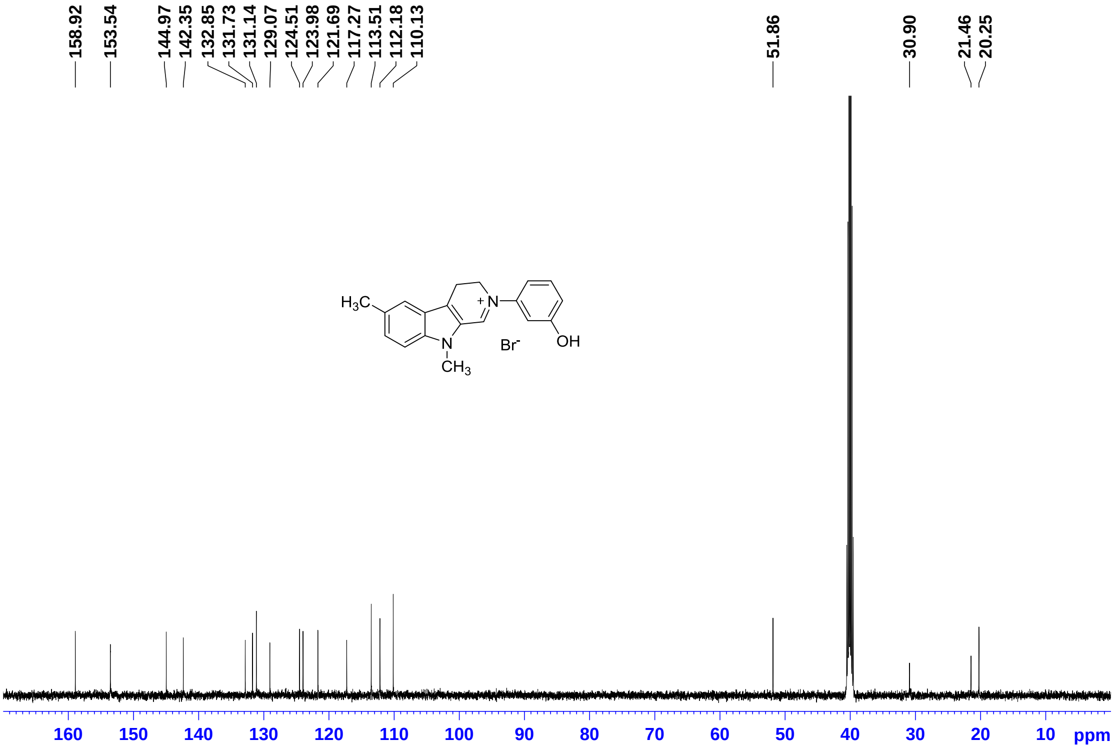

^{13}C NMR of Compound 6-21

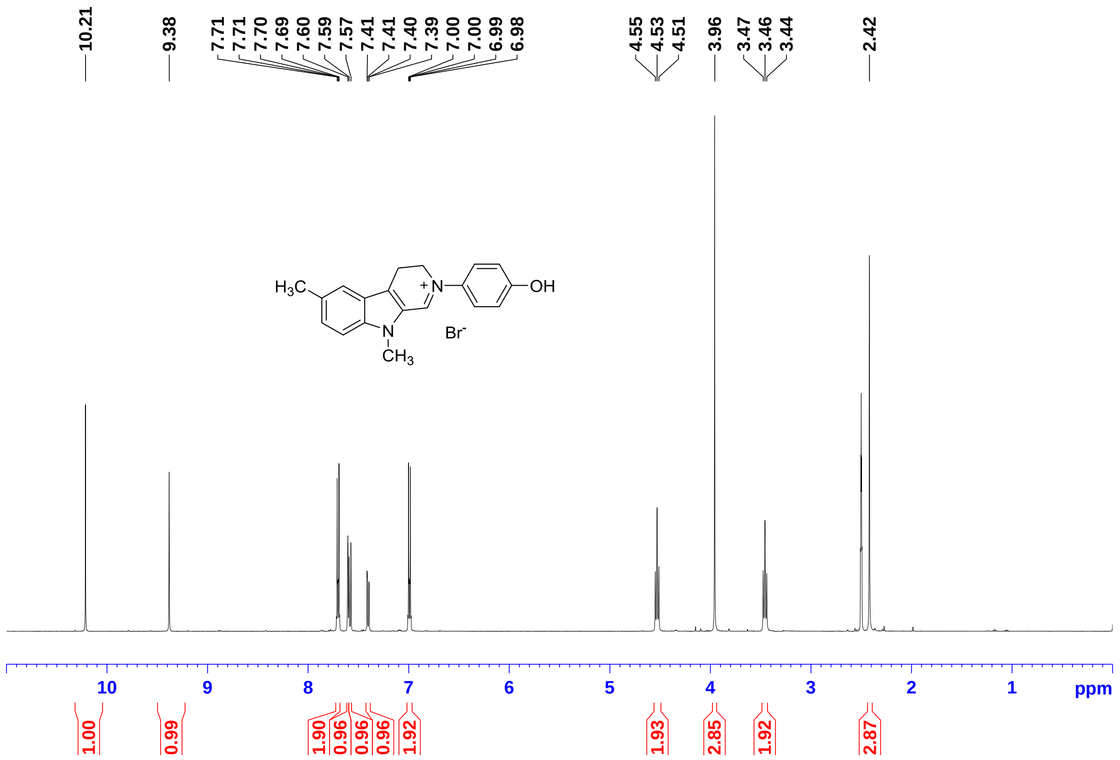

^{1}H NMR of Compound 6-22

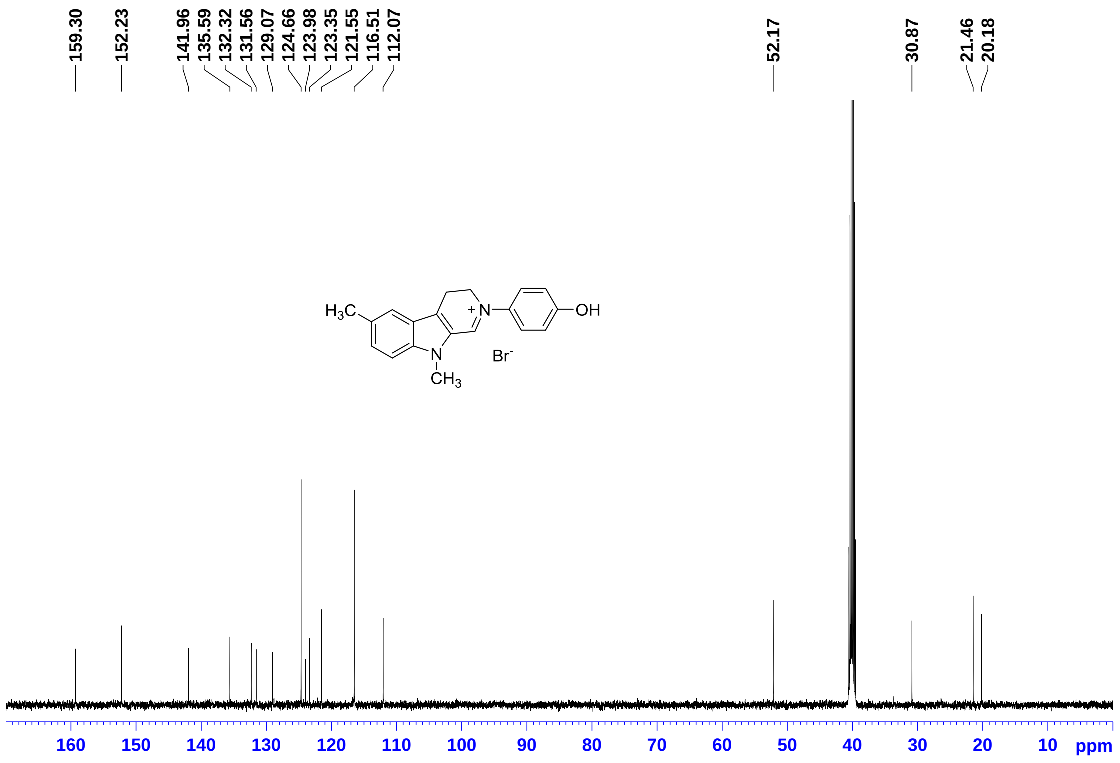

^{13}C NMR of Compound 6-22

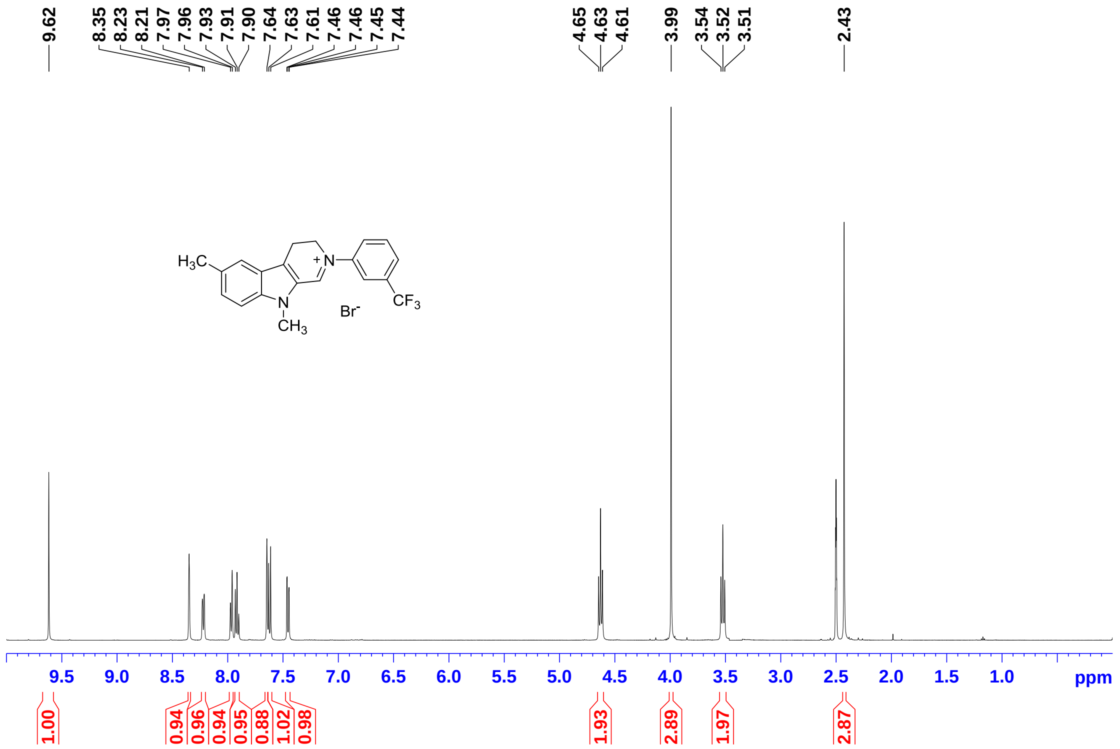

^{1}H NMR of Compound 6-23

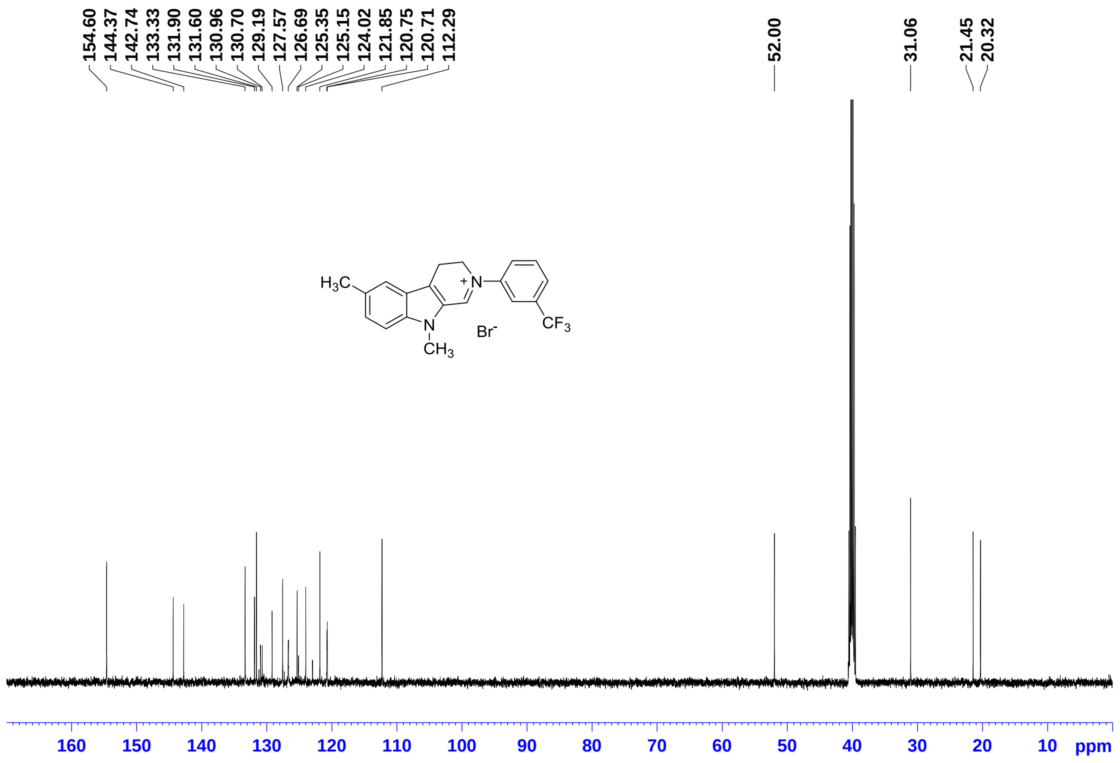

^{13}C NMR of Compound 6-23

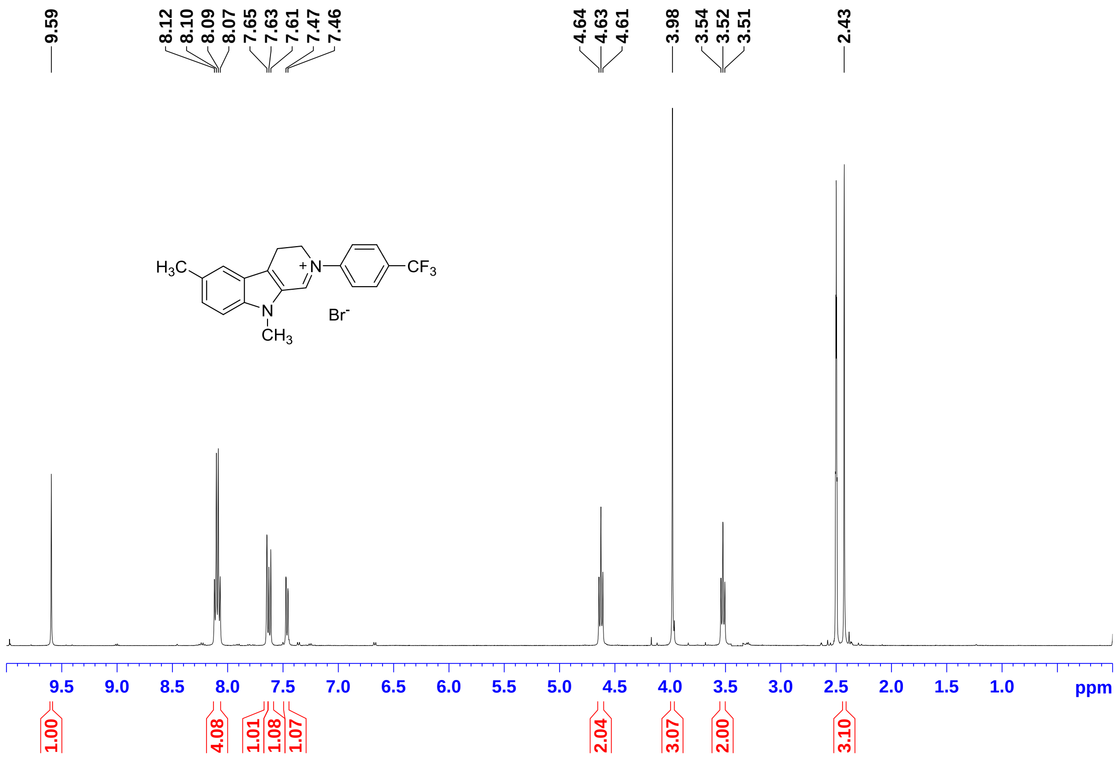

^{1}H NMR of Compound 6-24

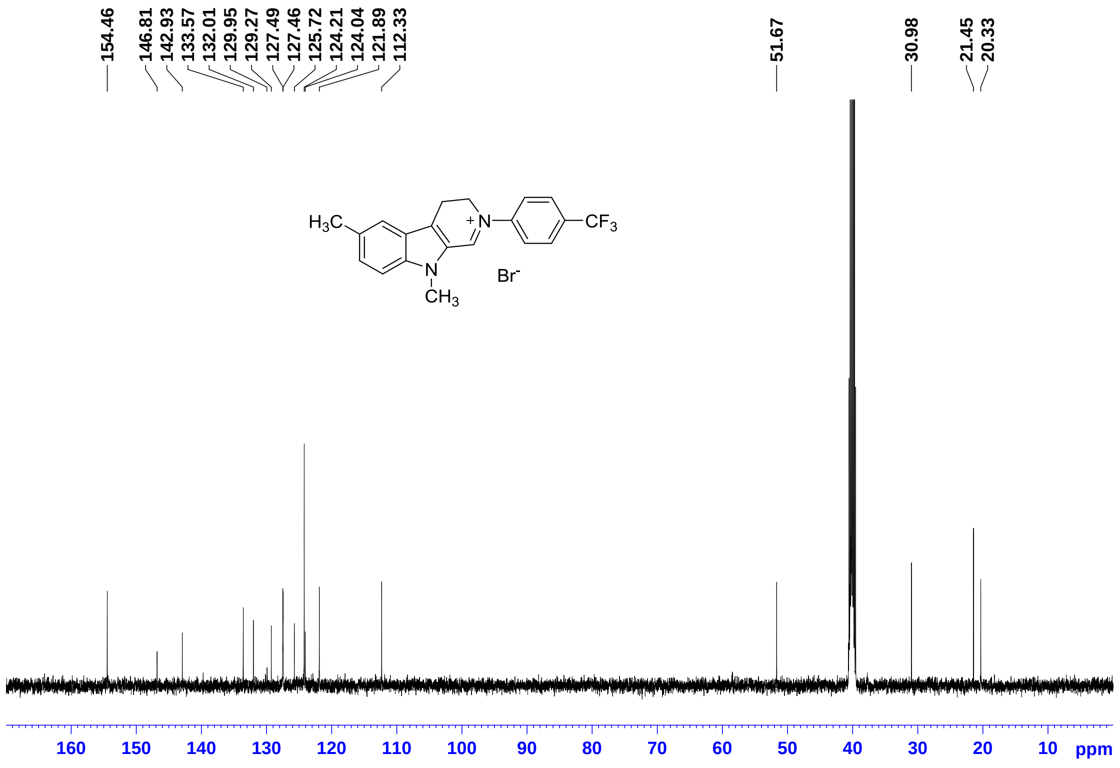

^{13}C NMR of Compound 6-24

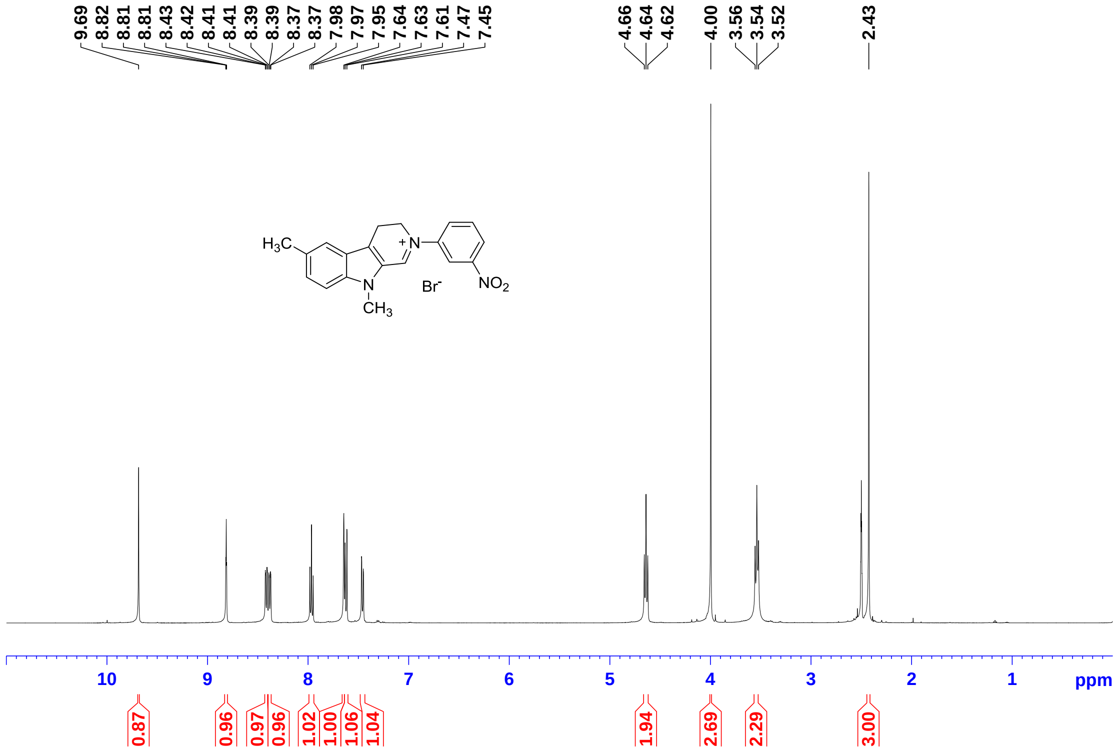

^{1}H NMR of Compound 6-25

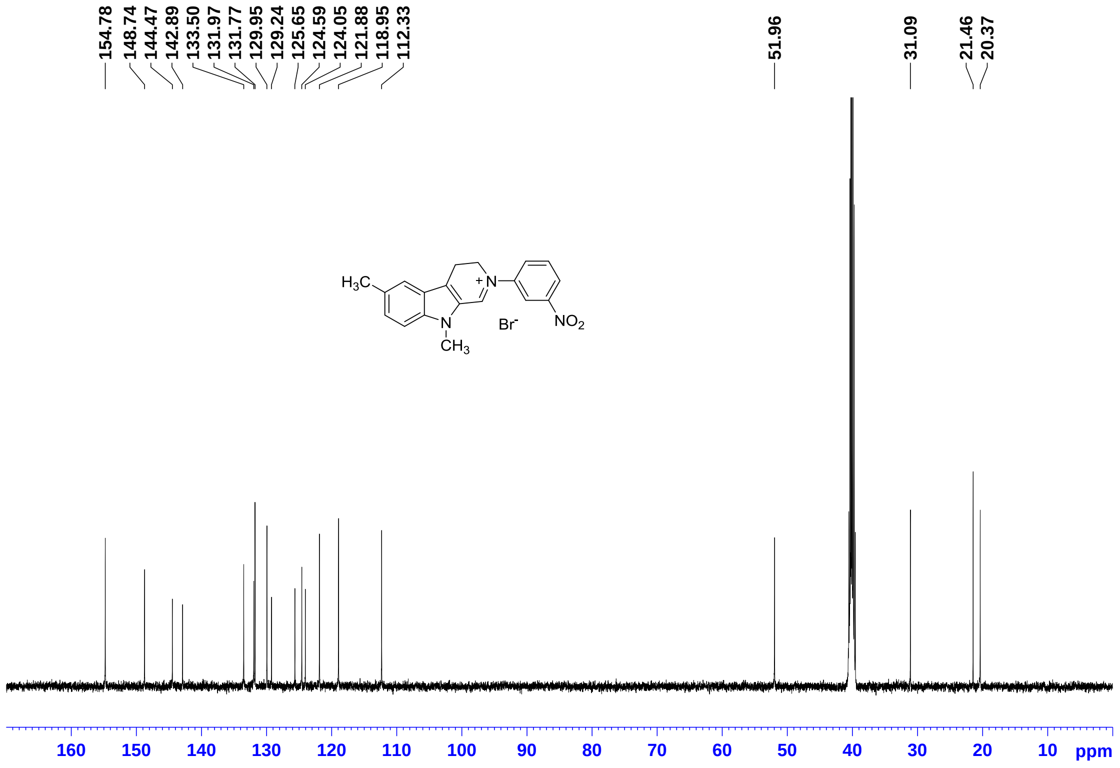

^{13}C NMR of Compound 6-25

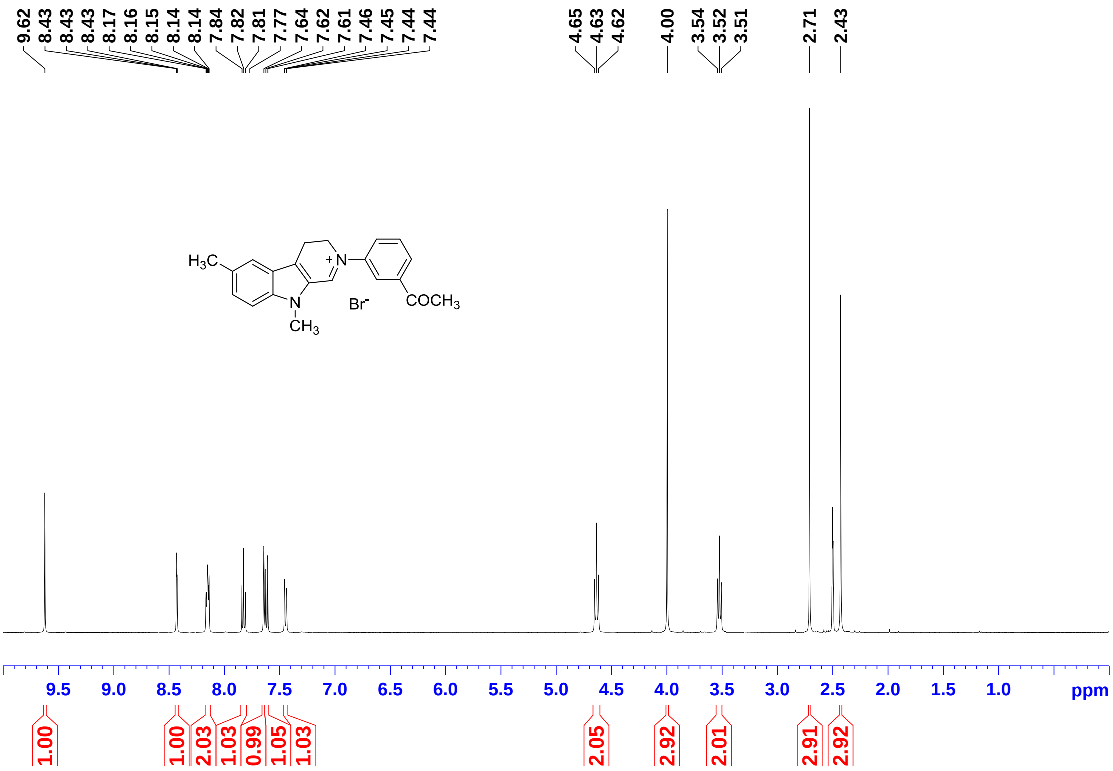

^{1}H NMR of Compound 6-26

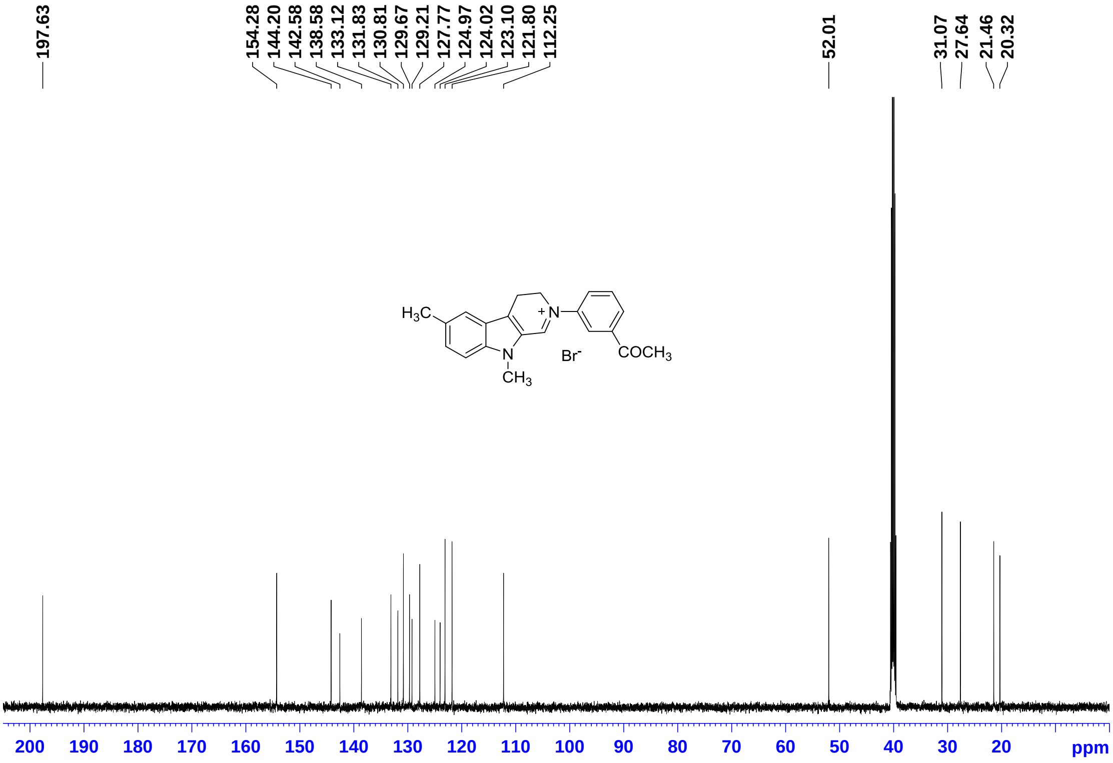

^{13}C NMR of Compound 6-26

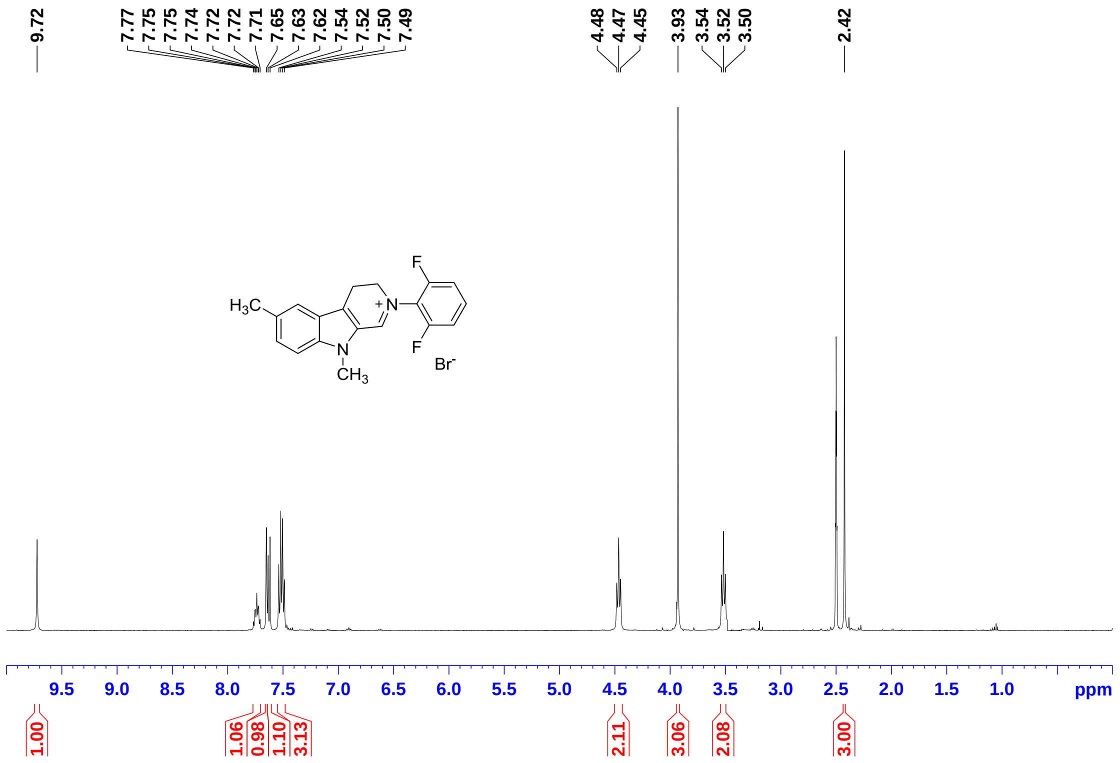

^{1}H NMR of Compound 6-27

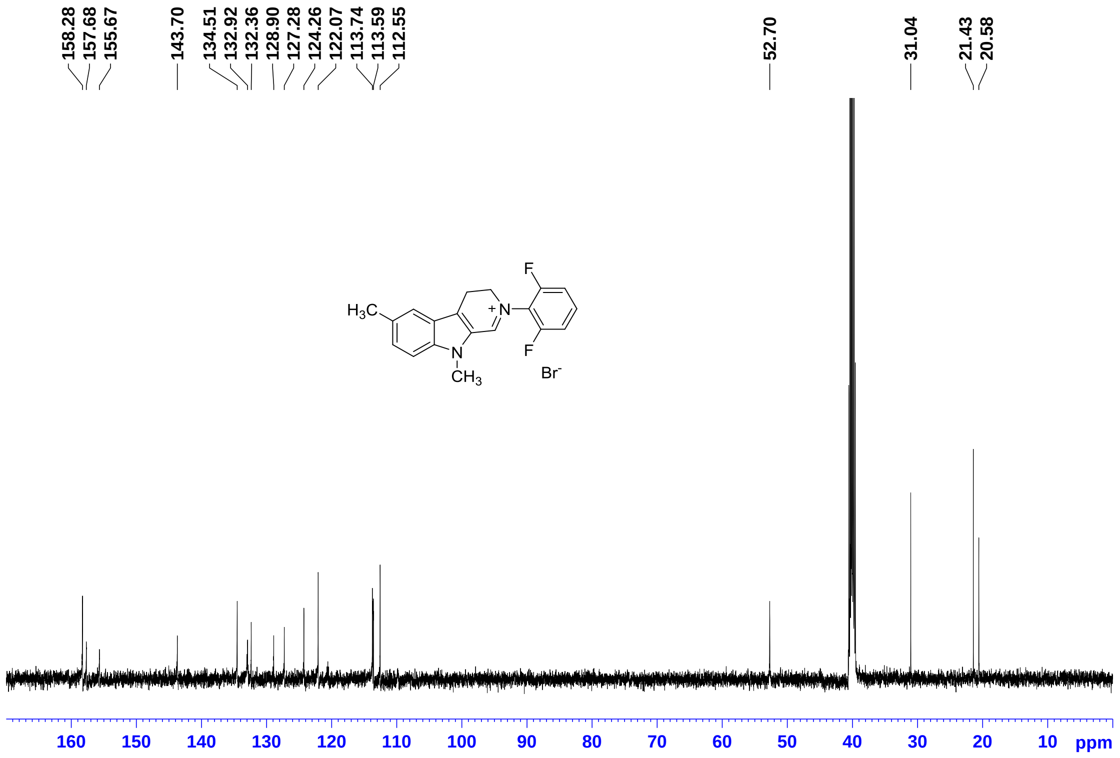

^{13}C NMR of Compound 6-27

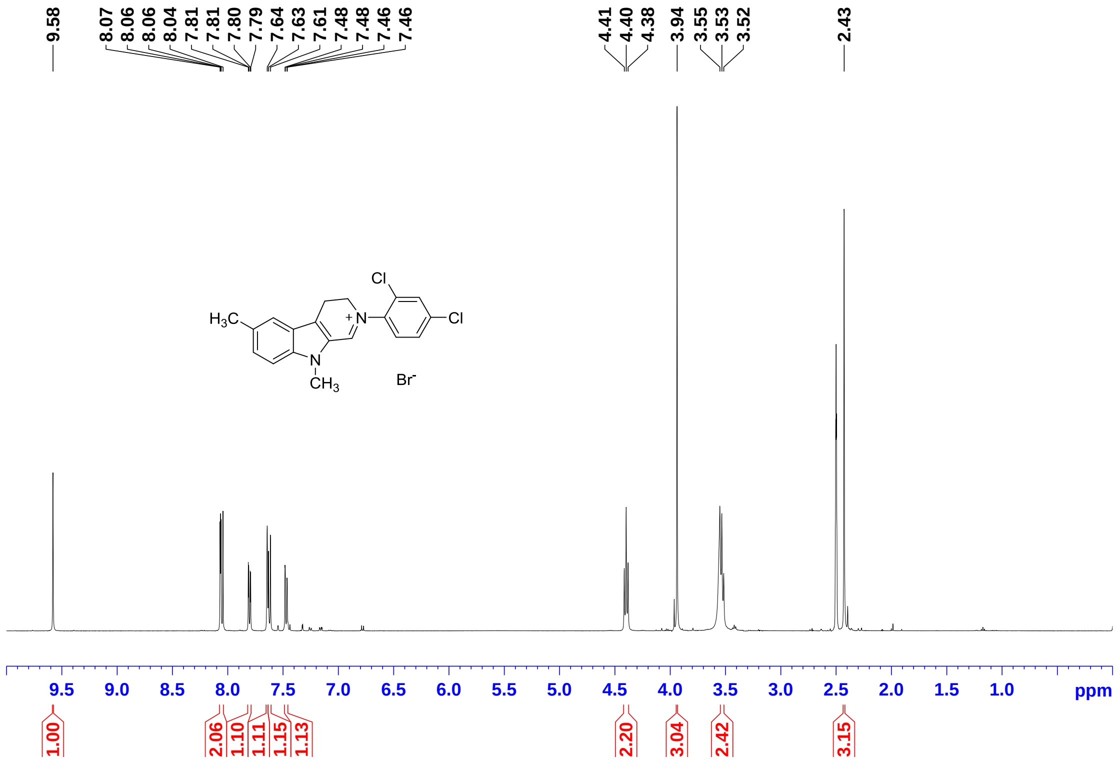

^{1}H NMR of Compound 6-28

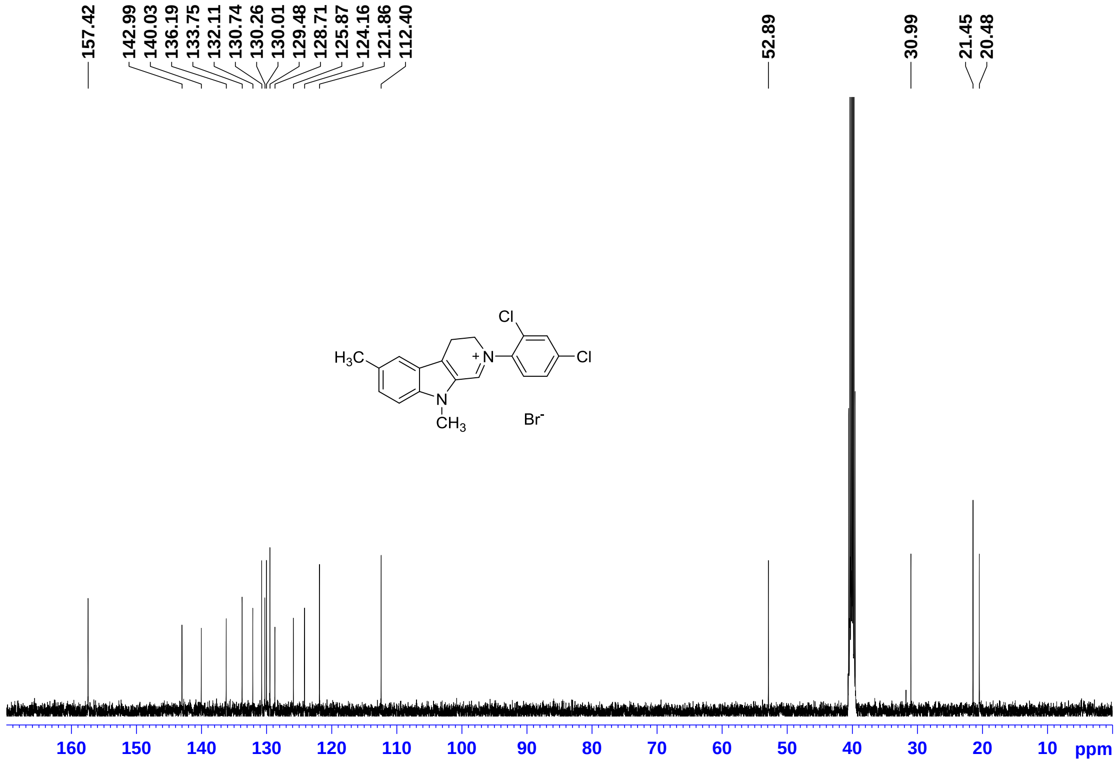

^{13}C NMR of Compound 6-28

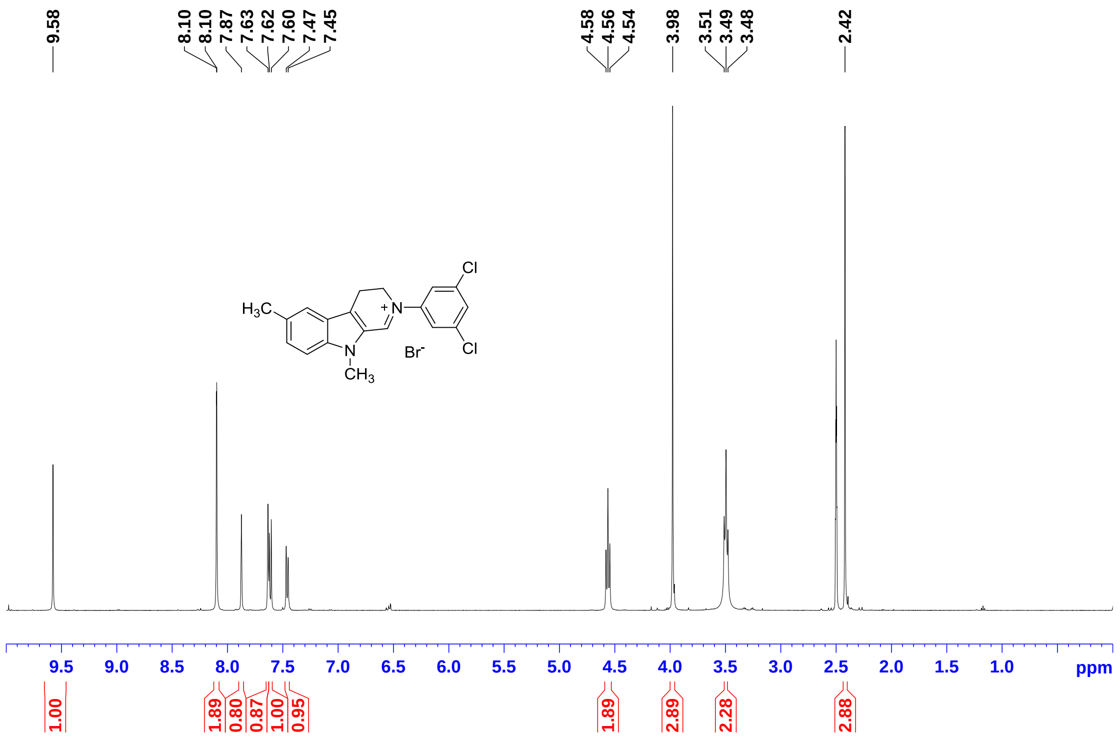

^{1}H NMR of Compound 6-29

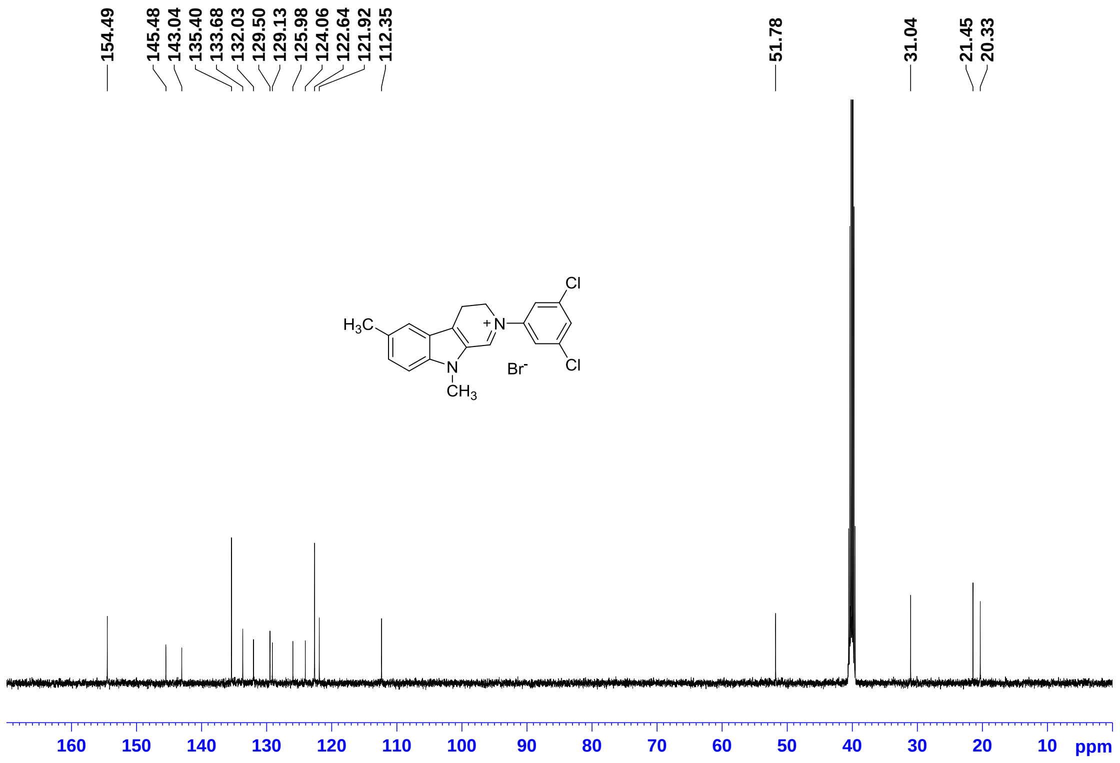

^{13}C NMR of Compound 6-29

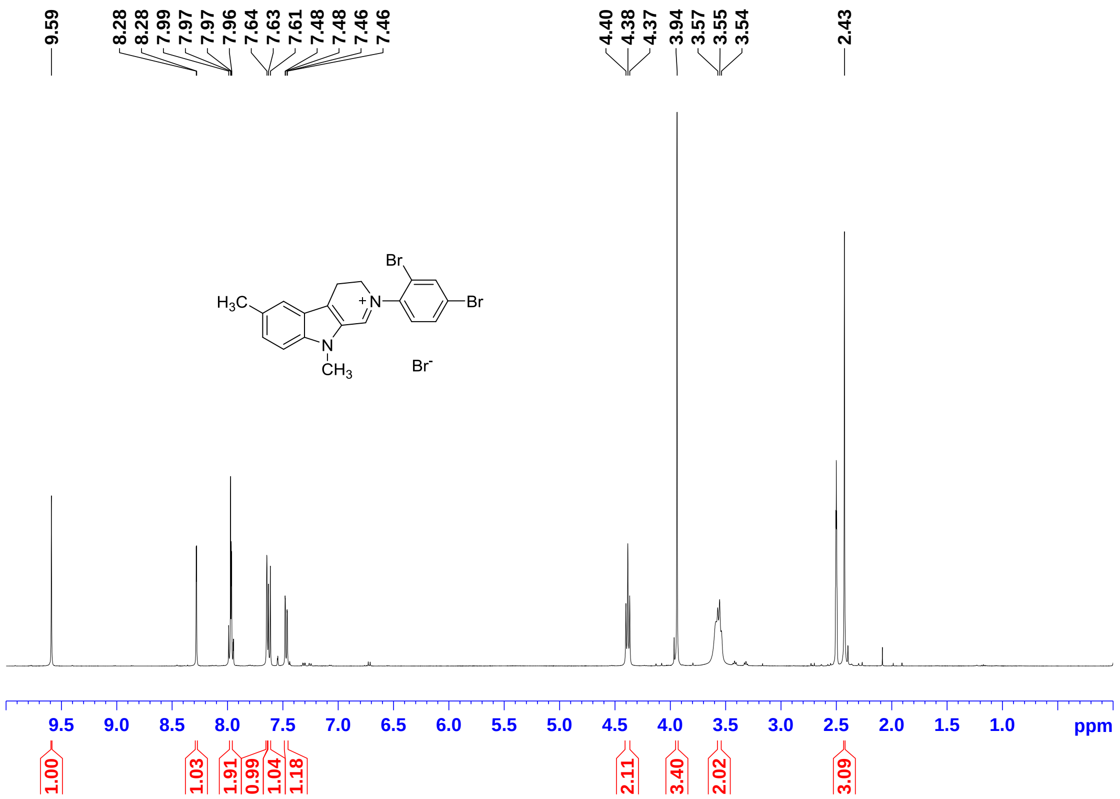

^{1}H NMR of Compound 6-30

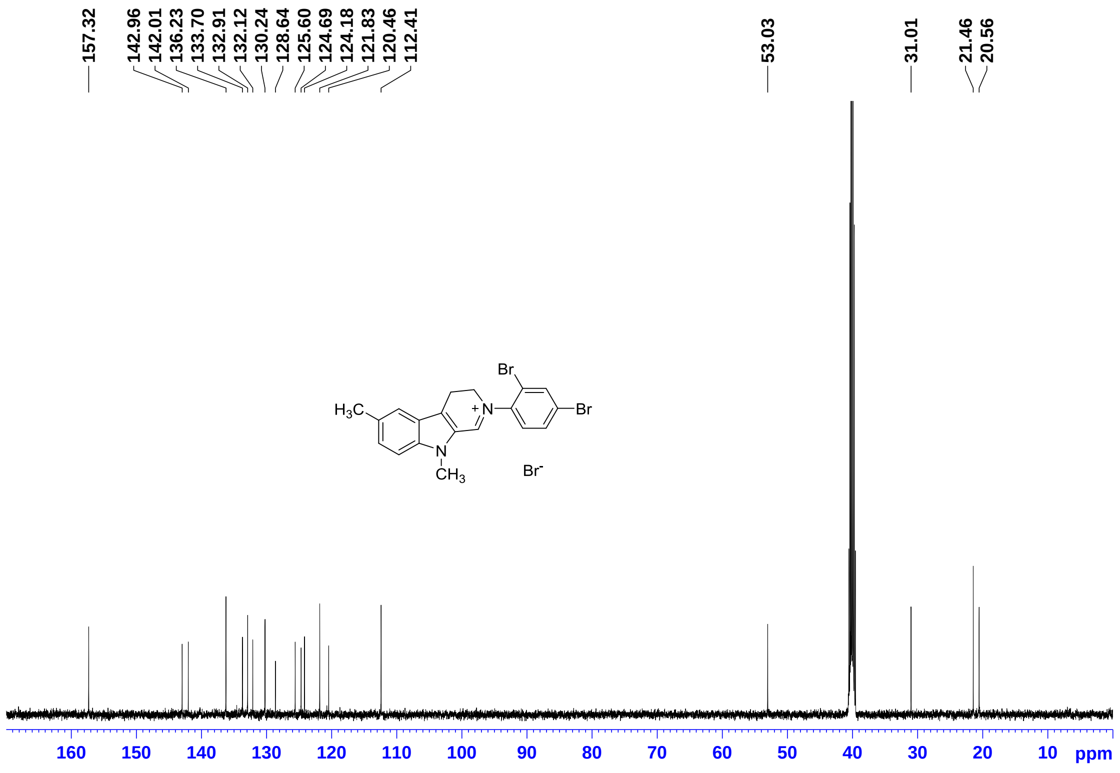

^{13}C NMR of Compound 6-30

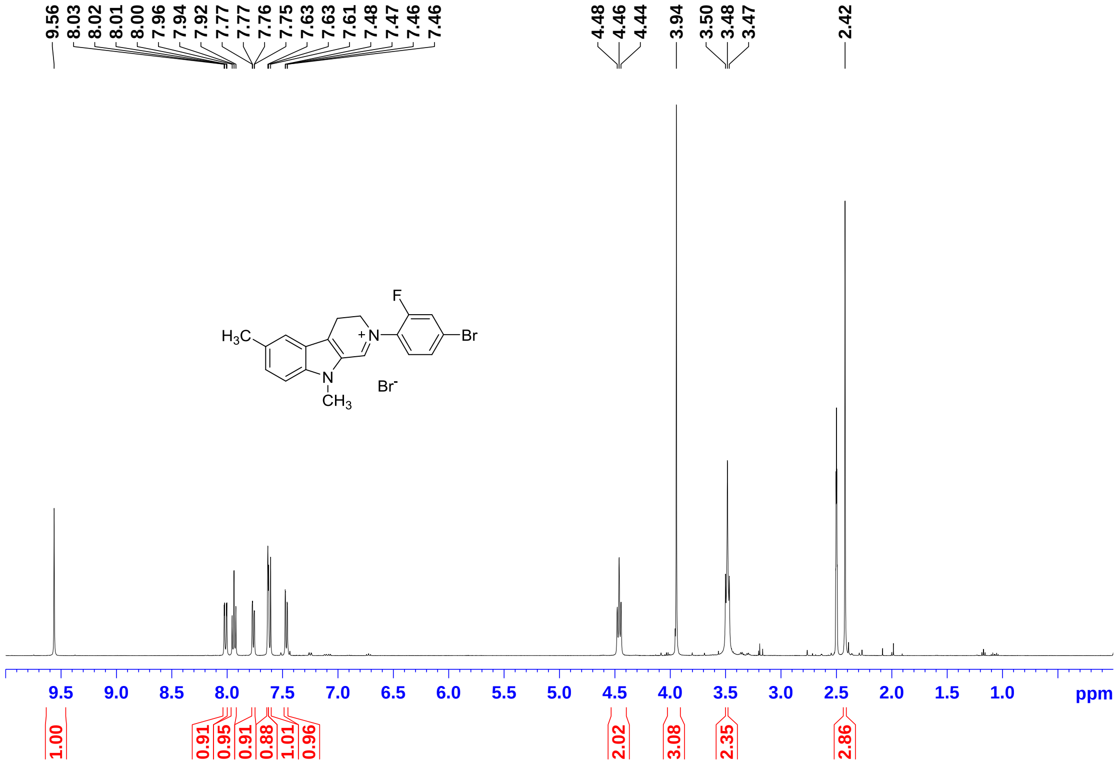

^{1}H NMR of Compound 6-31

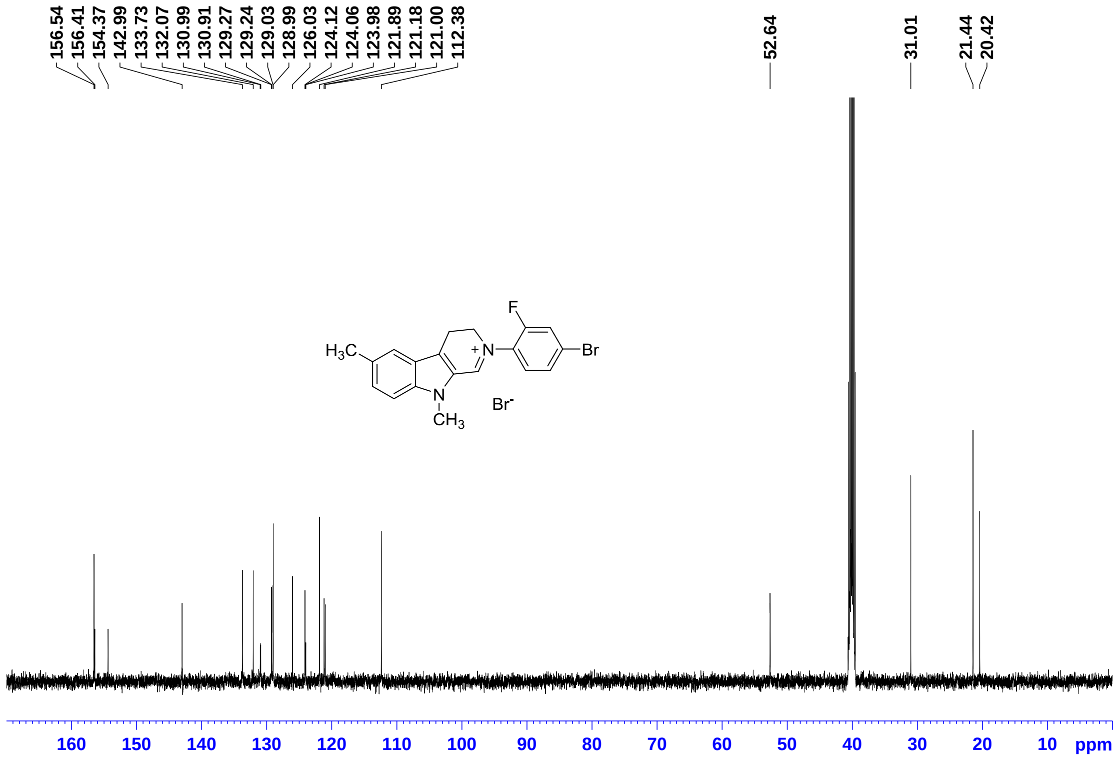

^{13}C NMR of Compound 6-31

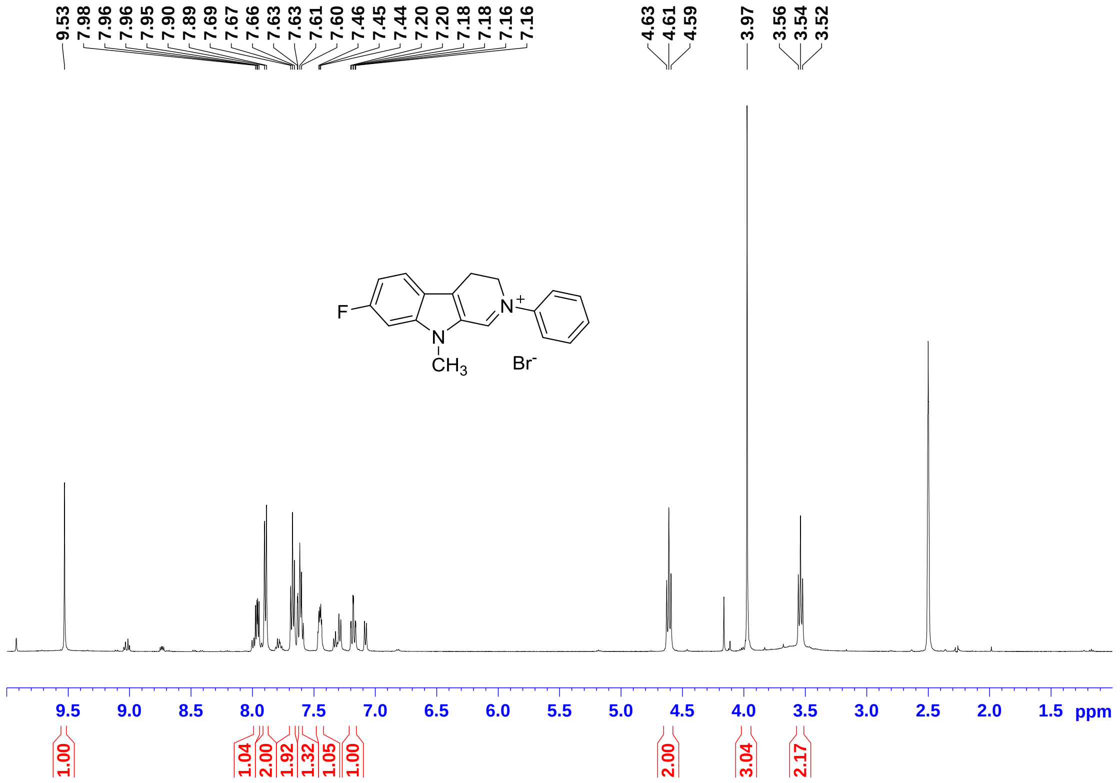

^{1}H NMR of Compound 6-32

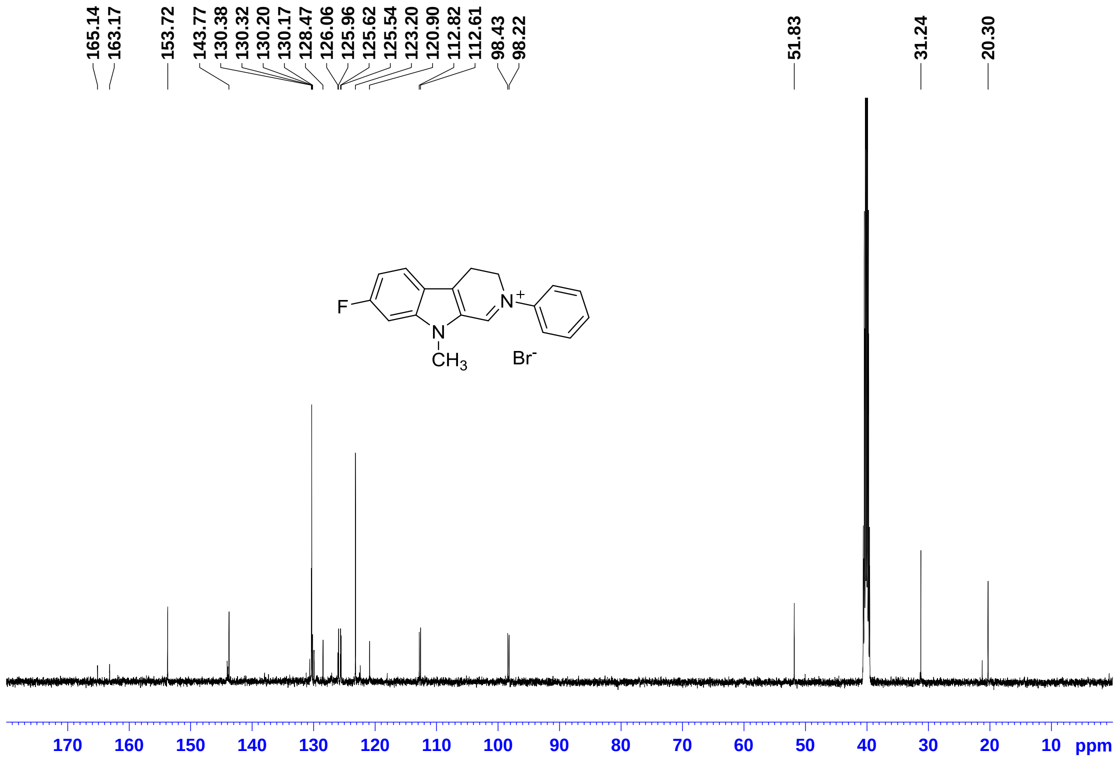

^{13}C NMR of Compound 6-32

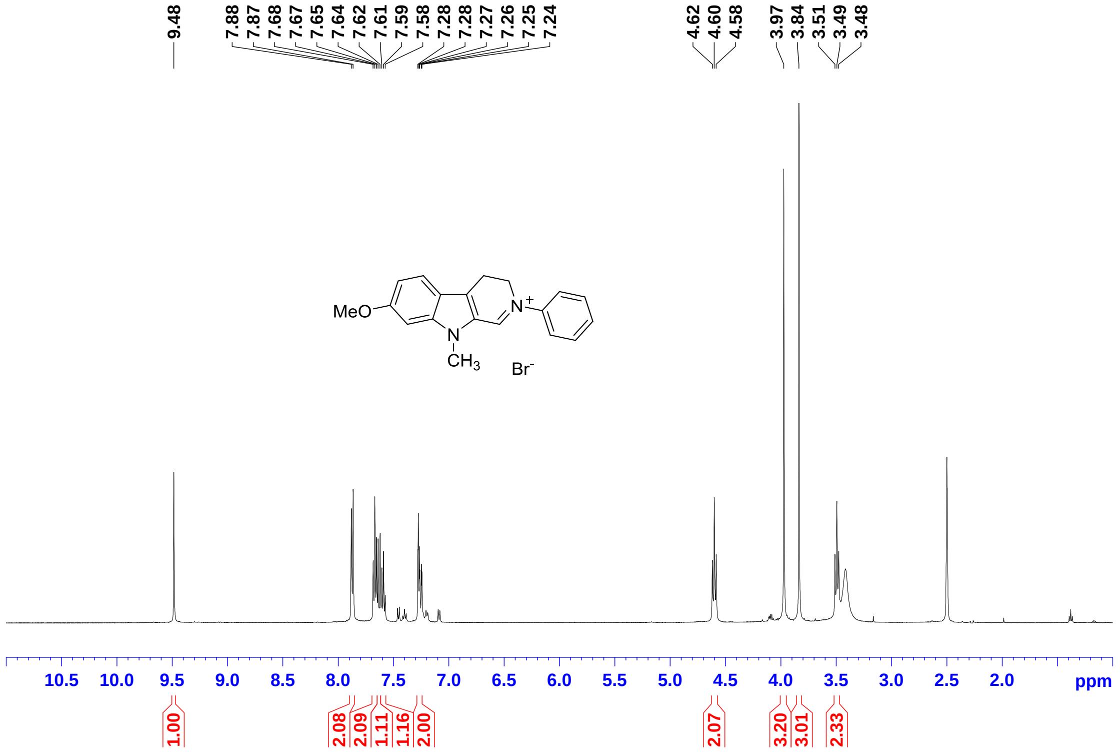

^{1}H NMR of Compound 6-33

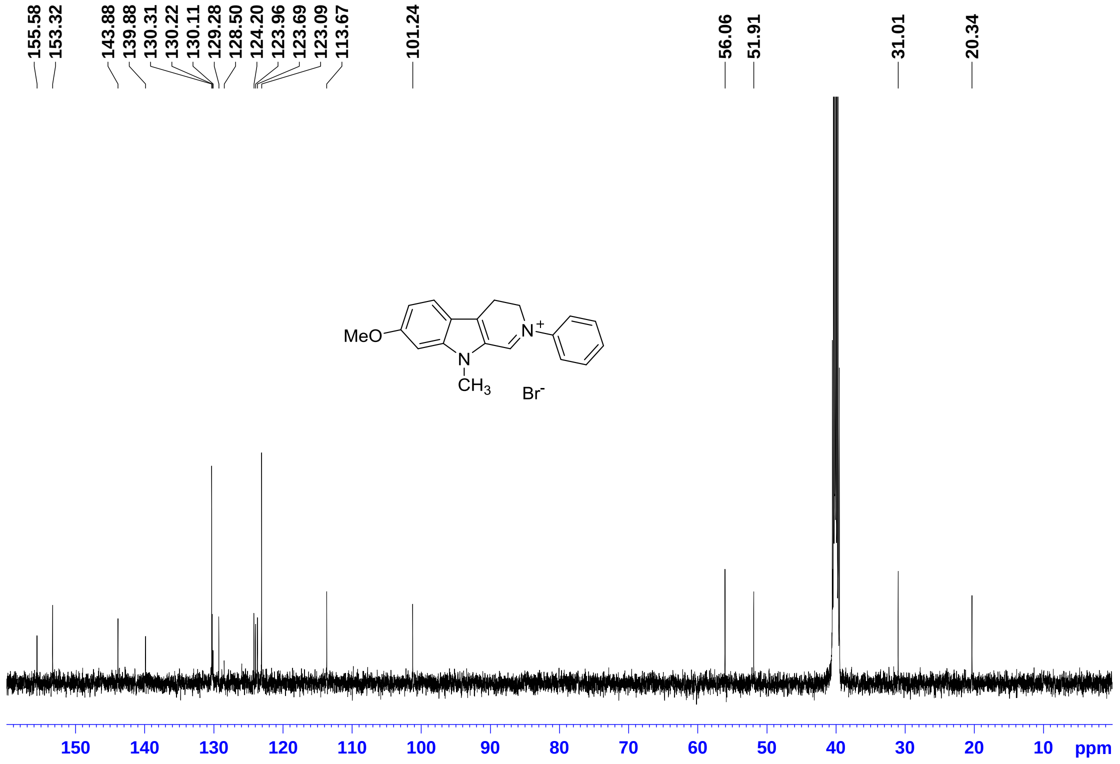

^{13}C NMR of Compound 6-33

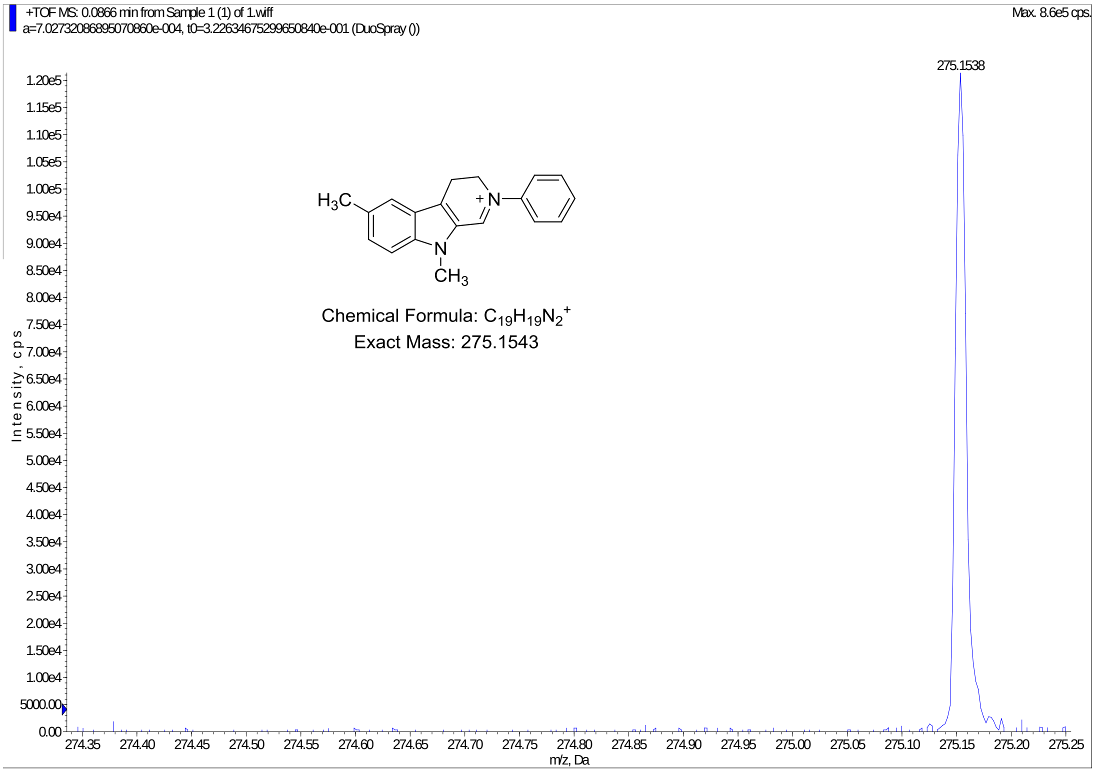

Positive ESI-HR-MS of Compound 6-1

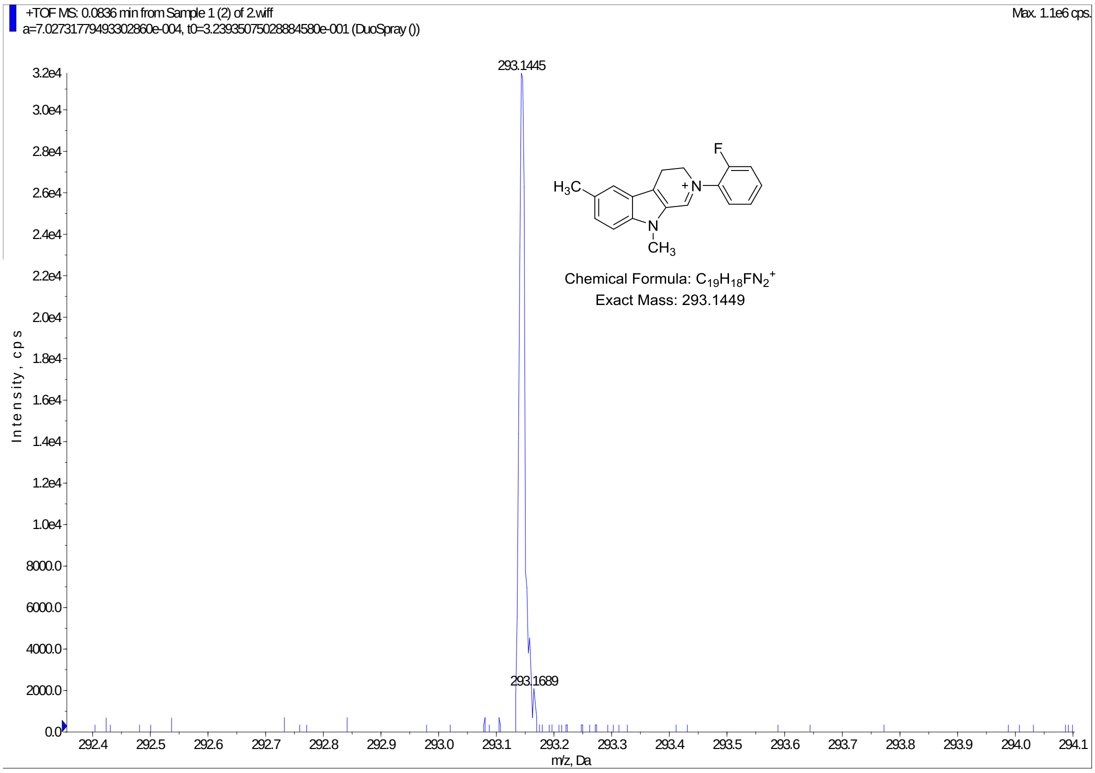

Positive ESI-HR-MS of Compound 6-2

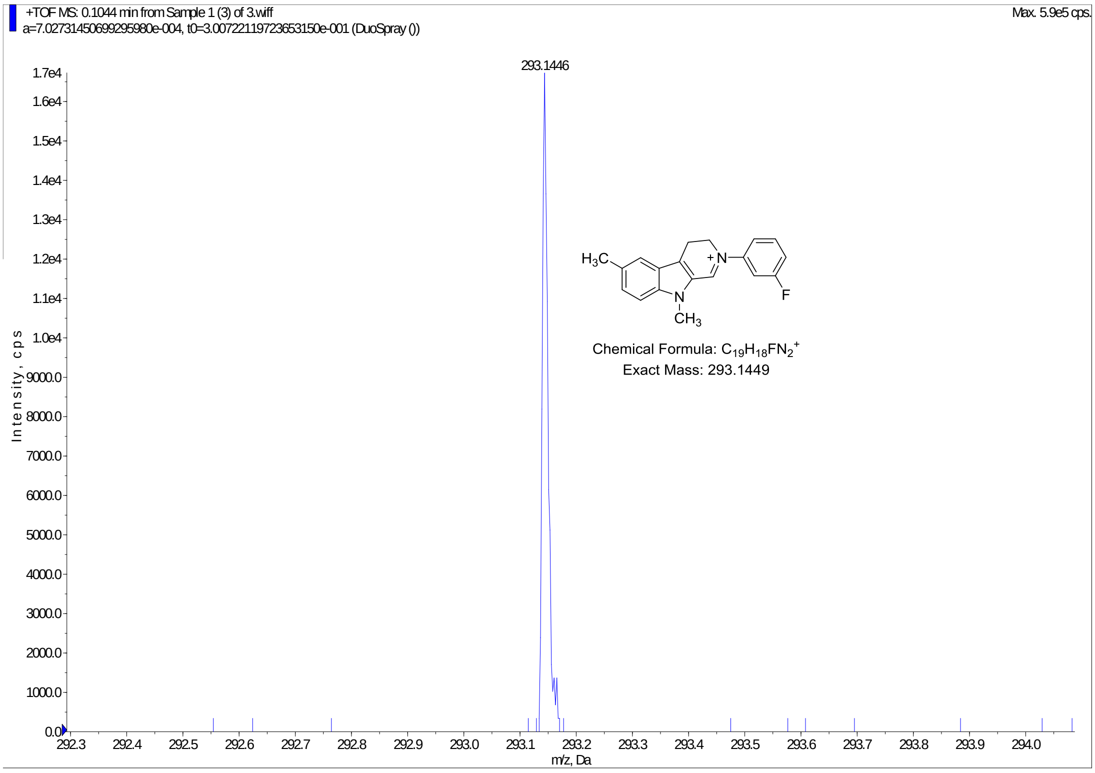

Positive ESI-HR-MS of Compound 6-3

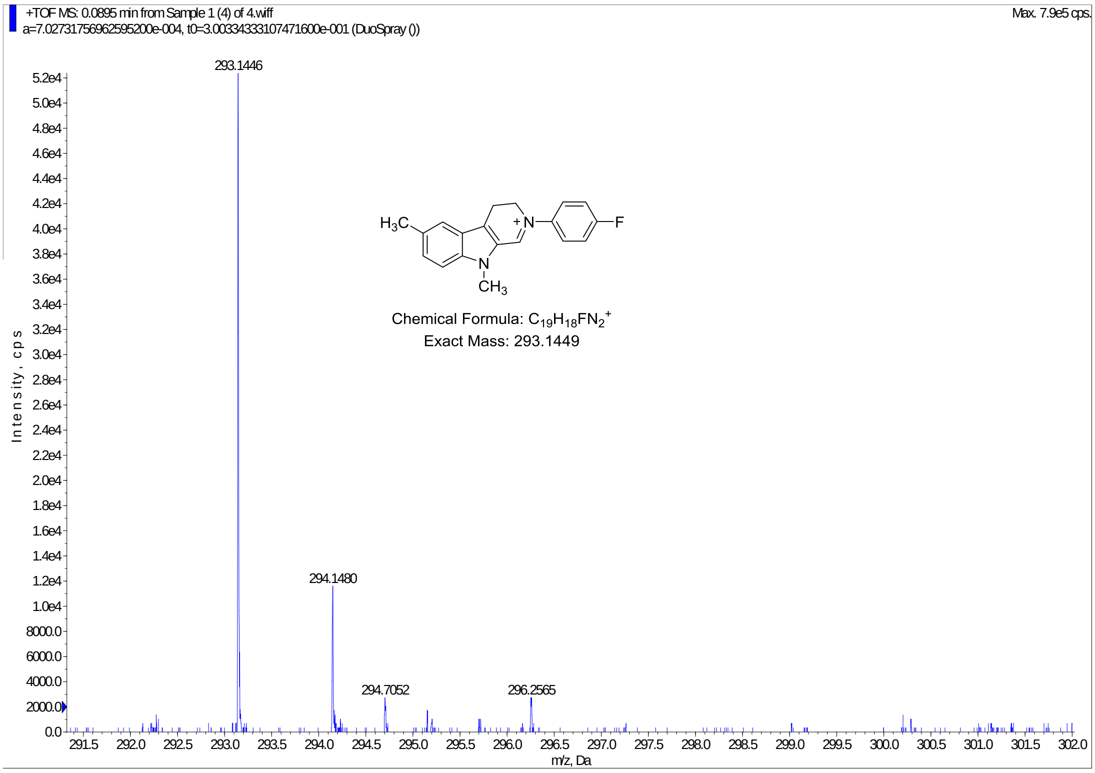

Positive ESI-HR-MS of Compound 6-4

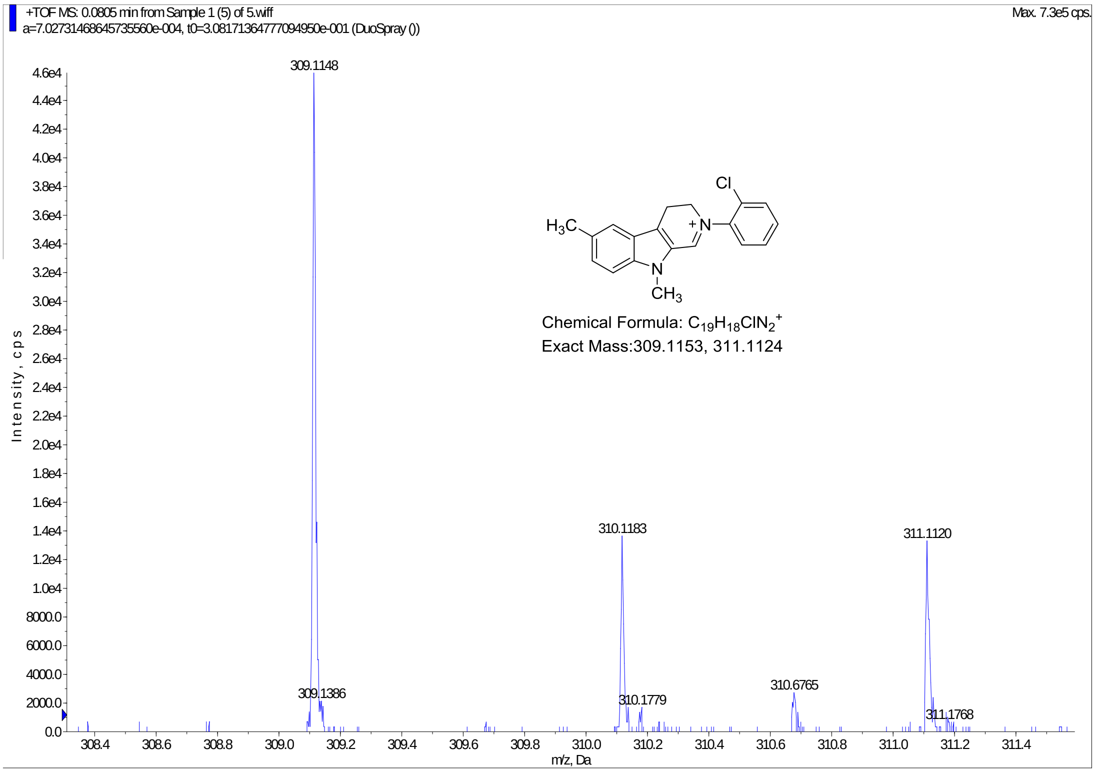

Positive ESI-HR-MS of Compound 6-5

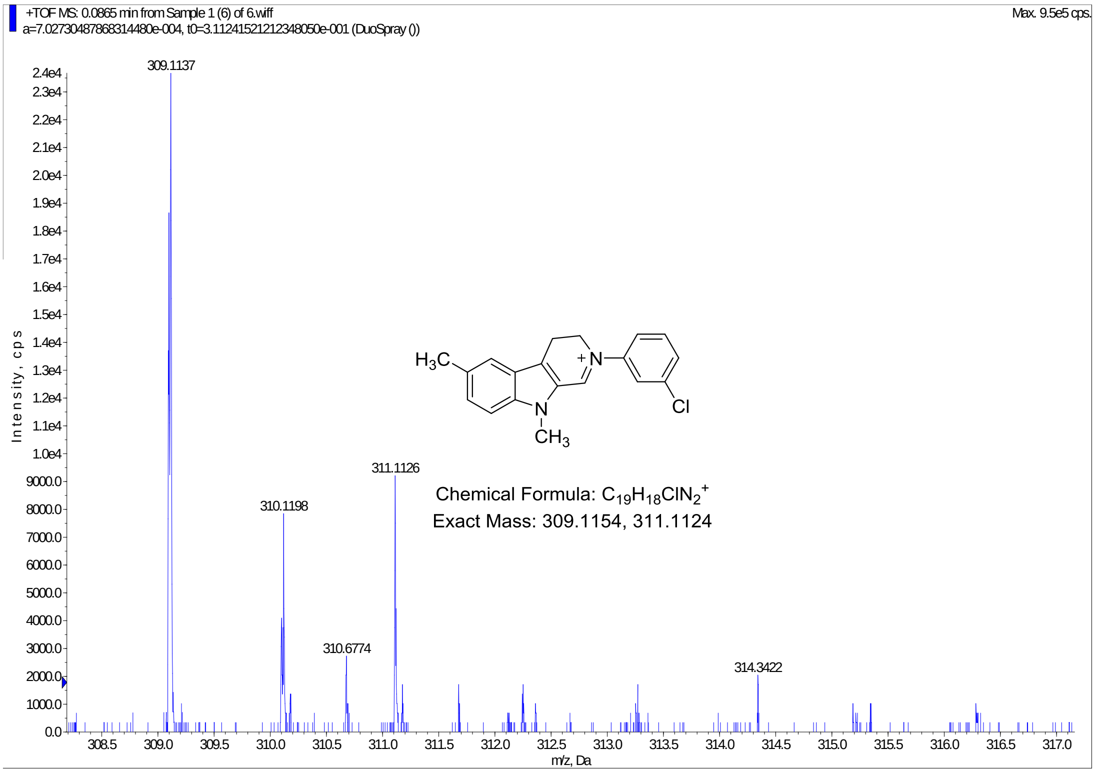

Positive ESI-HR-MS of Compound 6-6

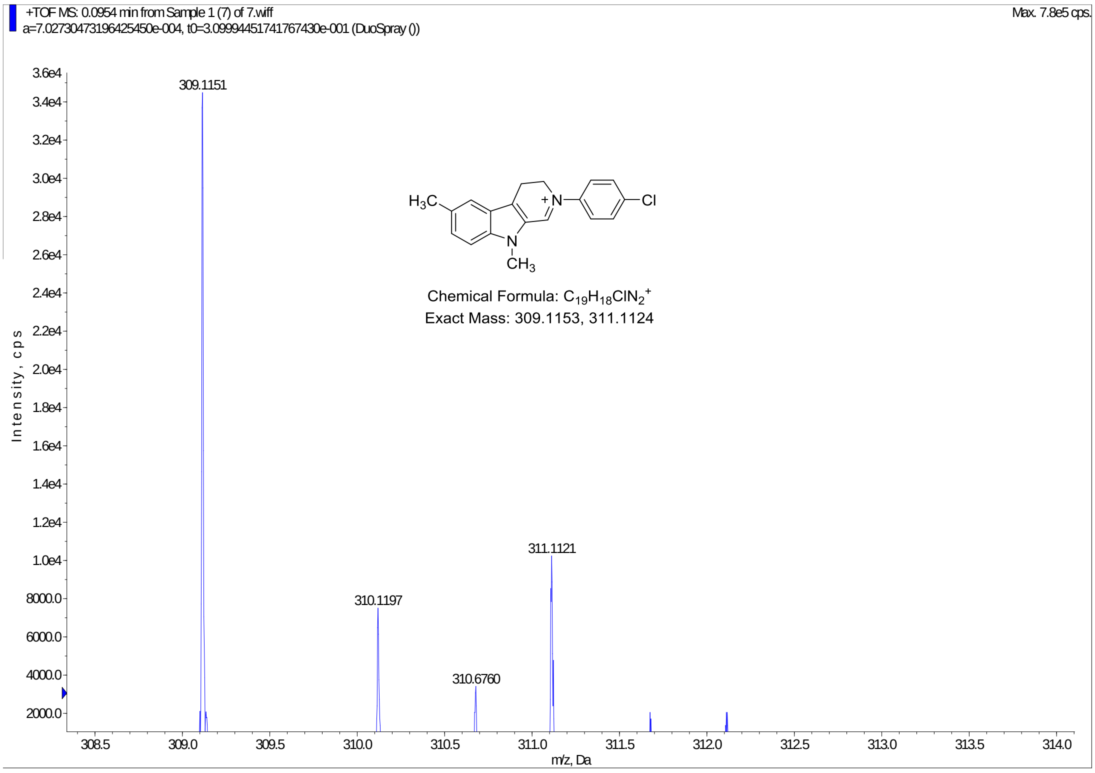

Positive ESI-HR-MS of Compound 6-7

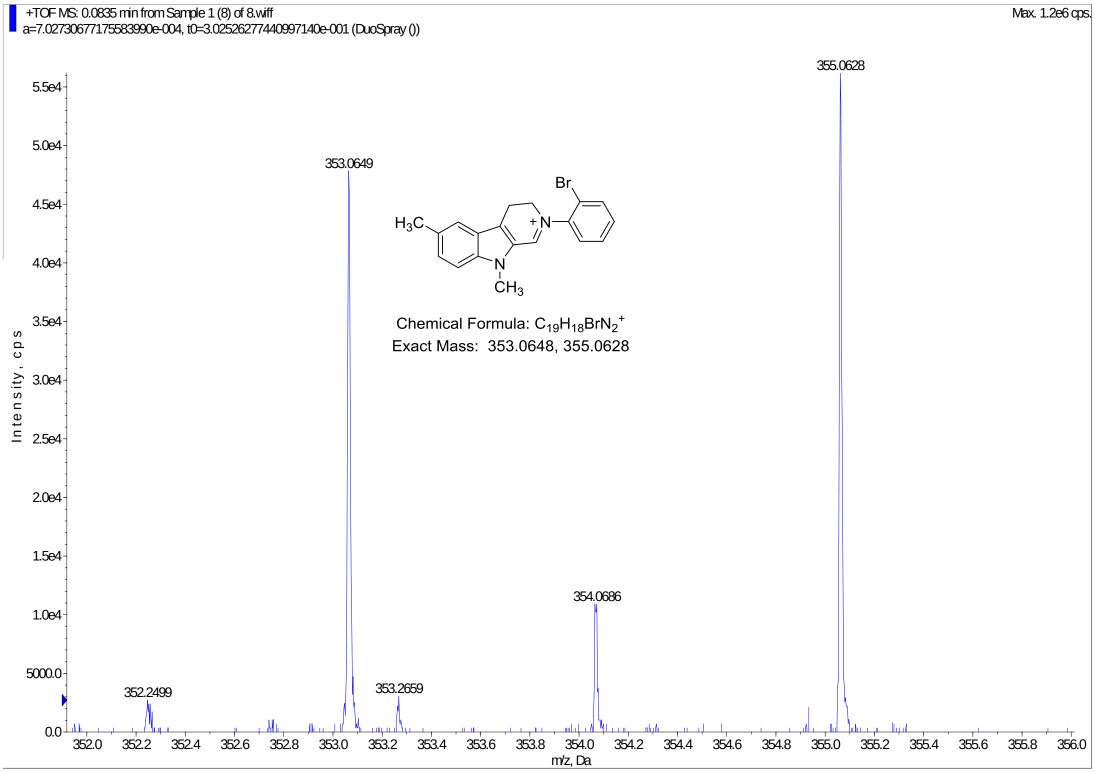

Positive ESI-HR-MS of Compound 6-8

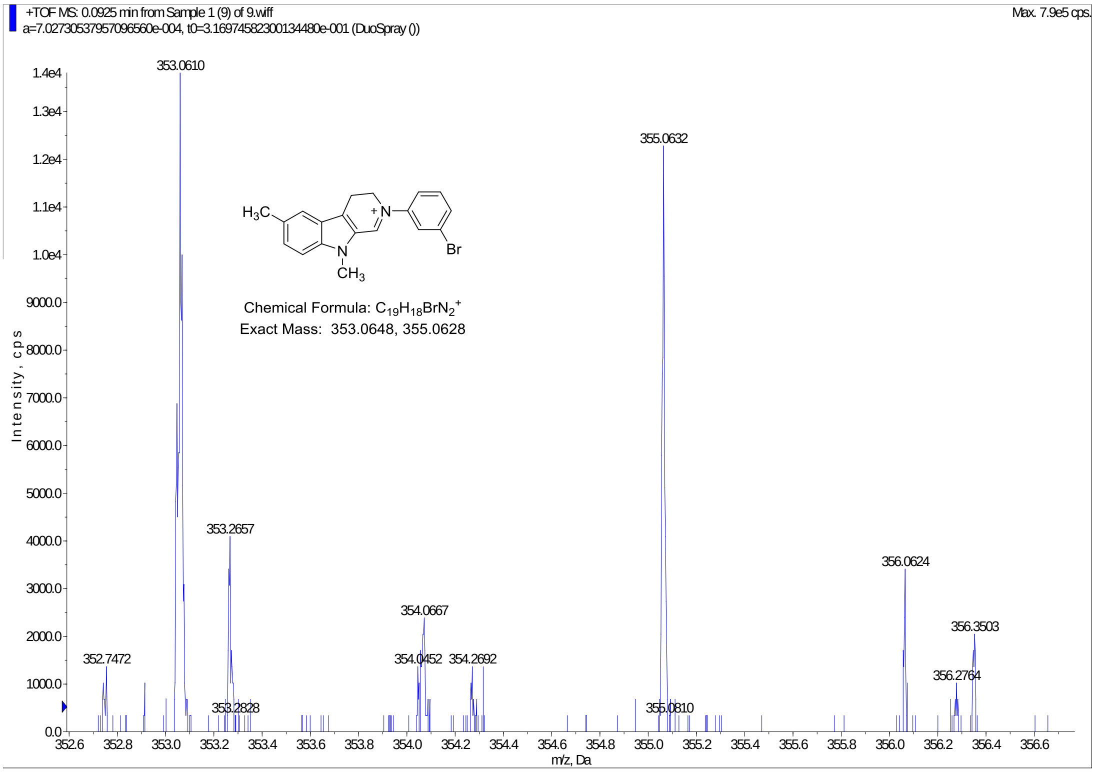

Positive ESI-HR-MS of Compound 6-9

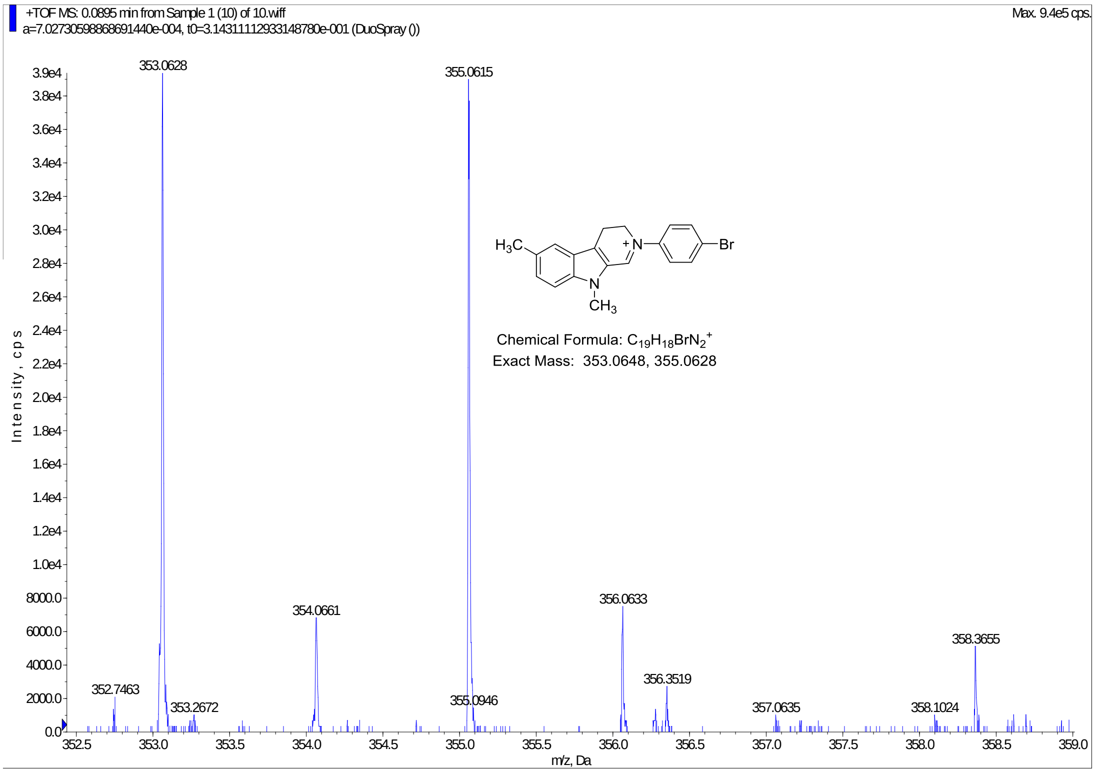

Positive ESI-HR-MS of Compound 6-10

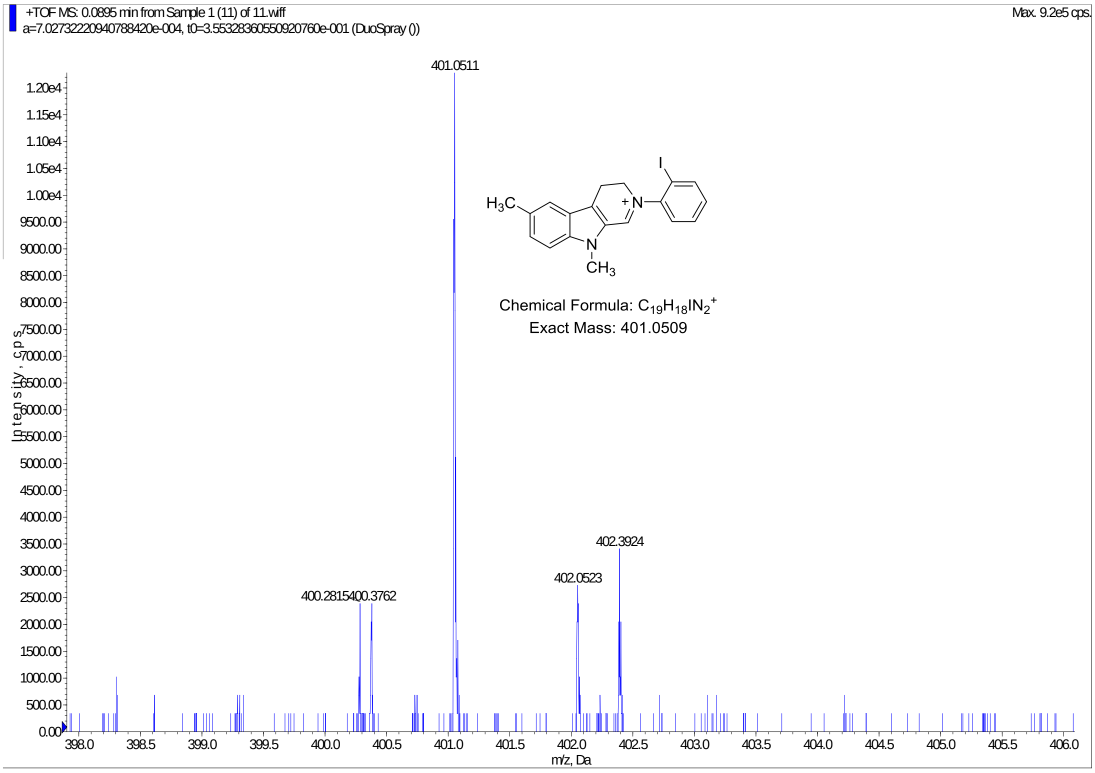

Positive ESI-HR-MS of Compound 6-11

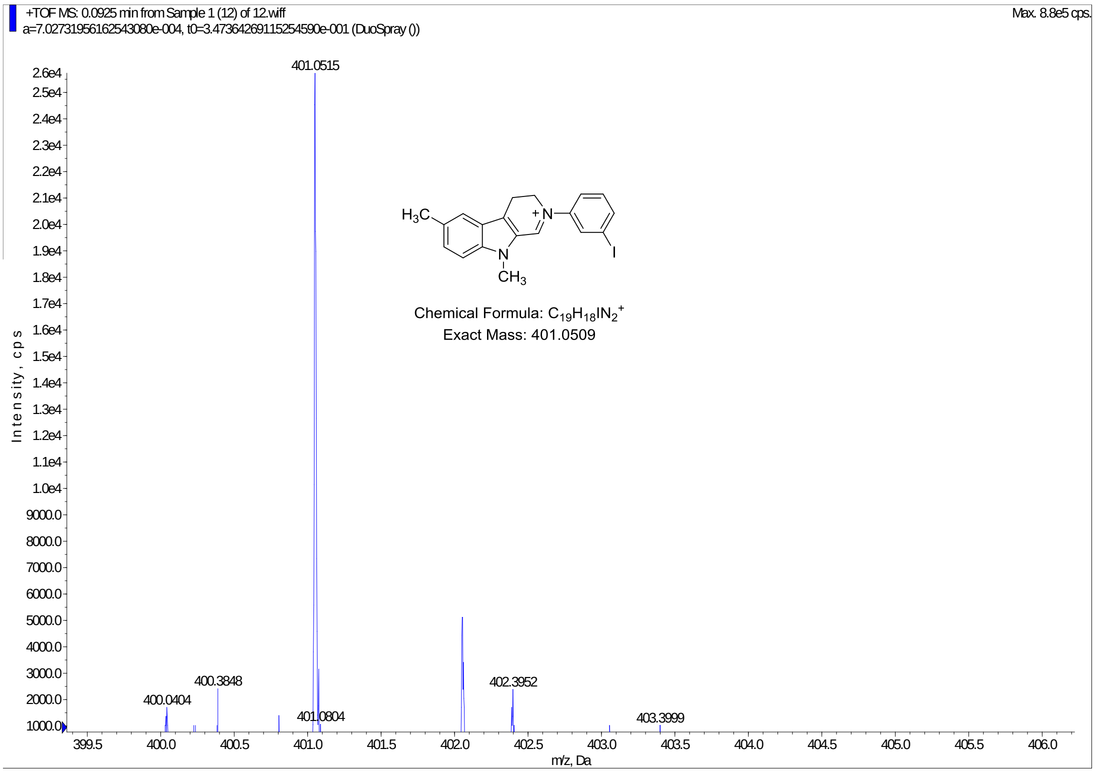

Positive ESI-HR-MS of Compound 6-12

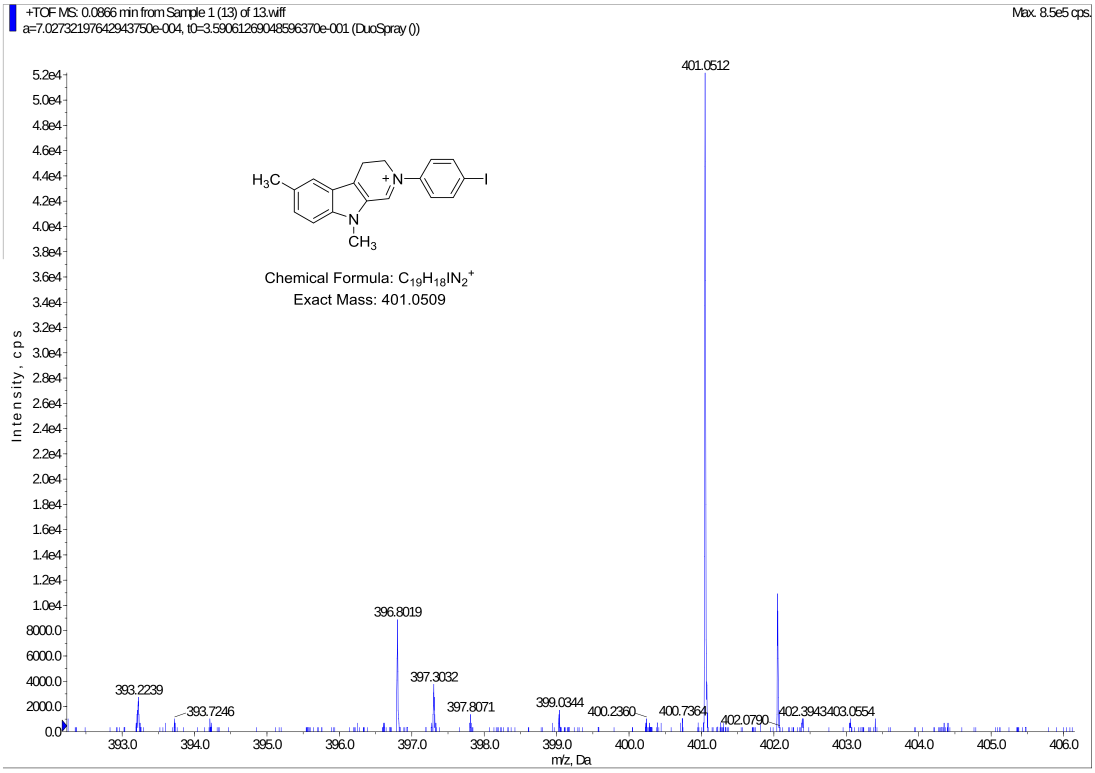

Positive ESI-HR-MS of Compound 6-13

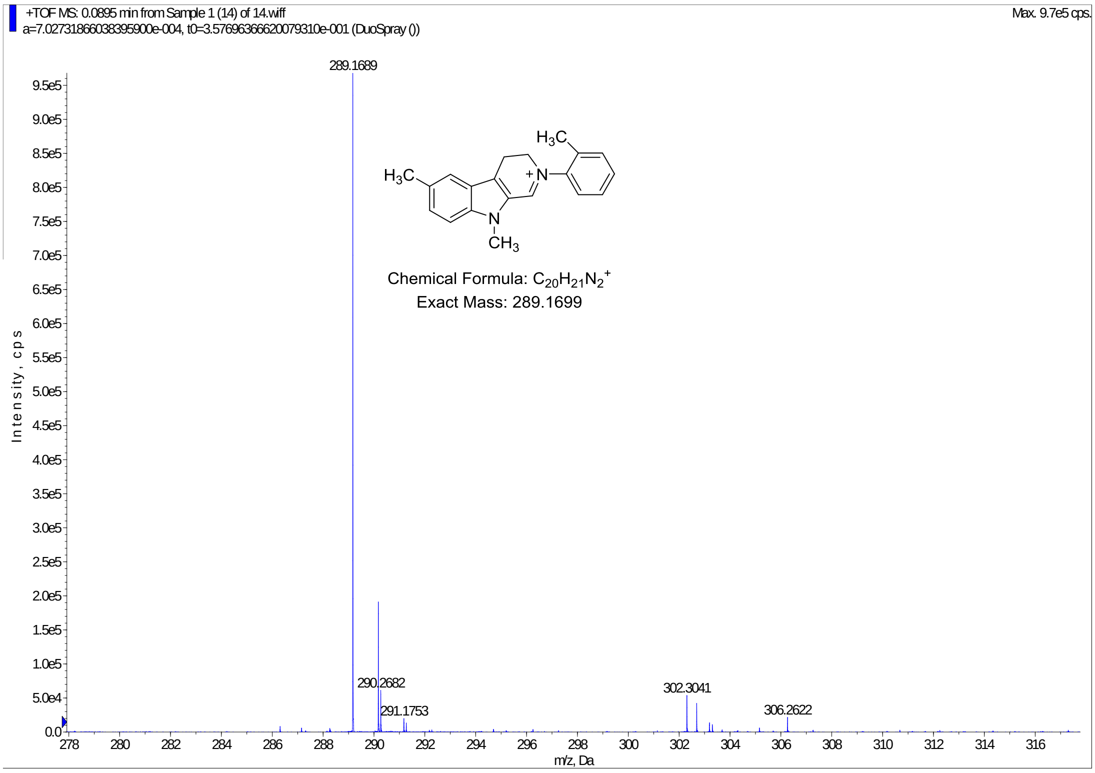

Positive ESI-HR-MS of Compound 6-14

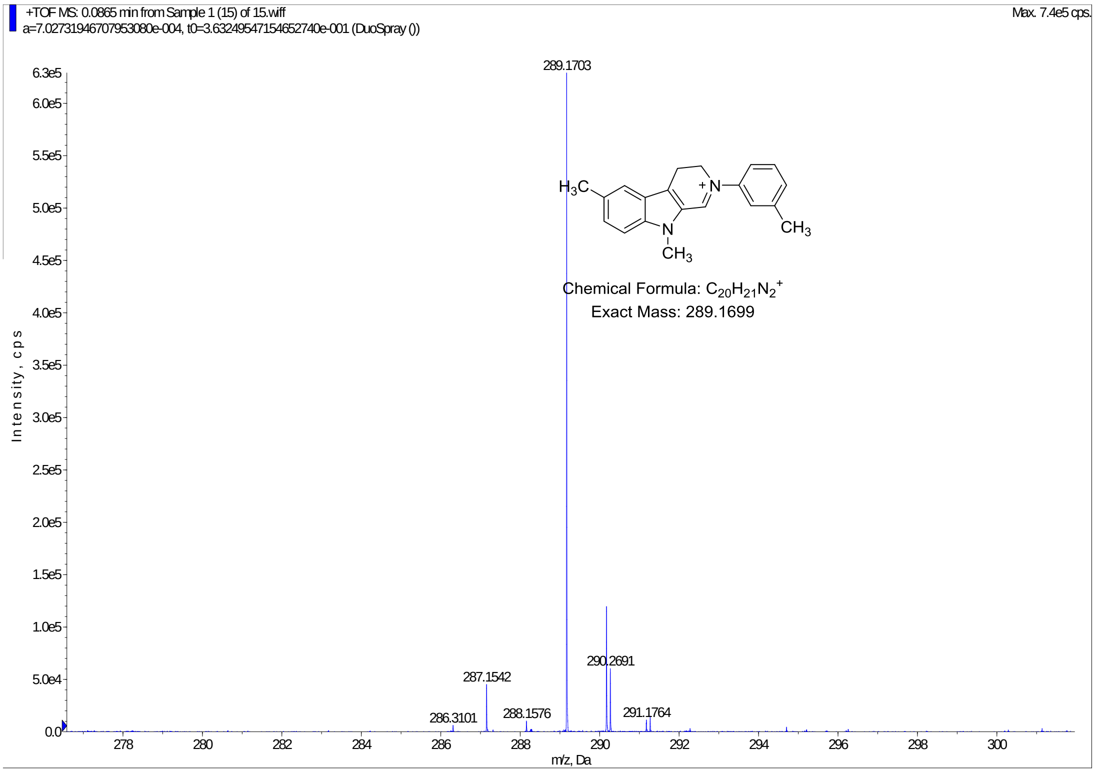

Positive ESI-HR-MS of Compound 6-15

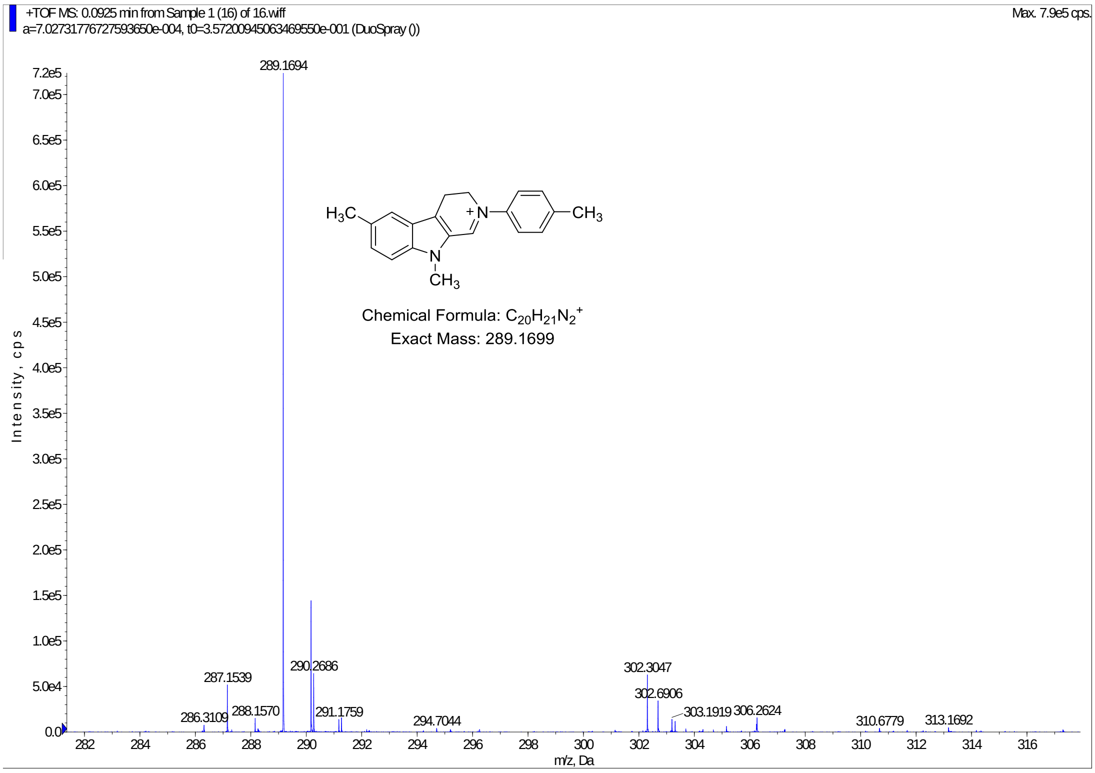

Positive ESI-HR-MS of Compound 6-16

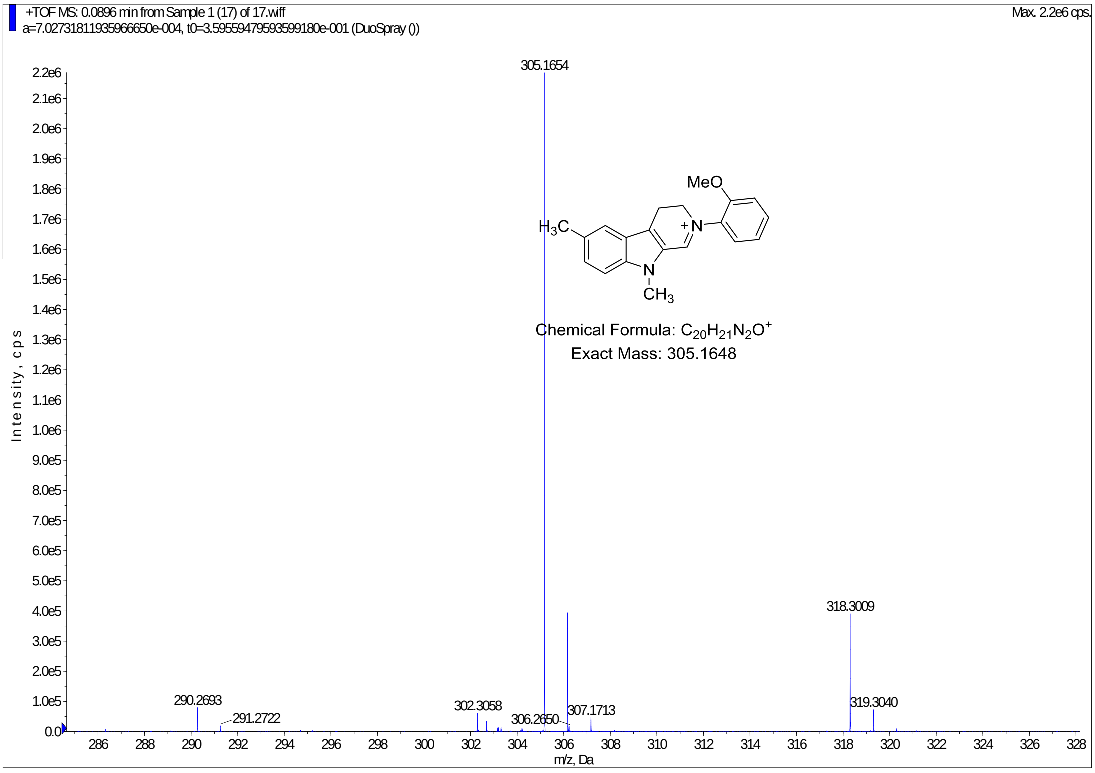

Positive ESI-HR-MS of Compound 6-17

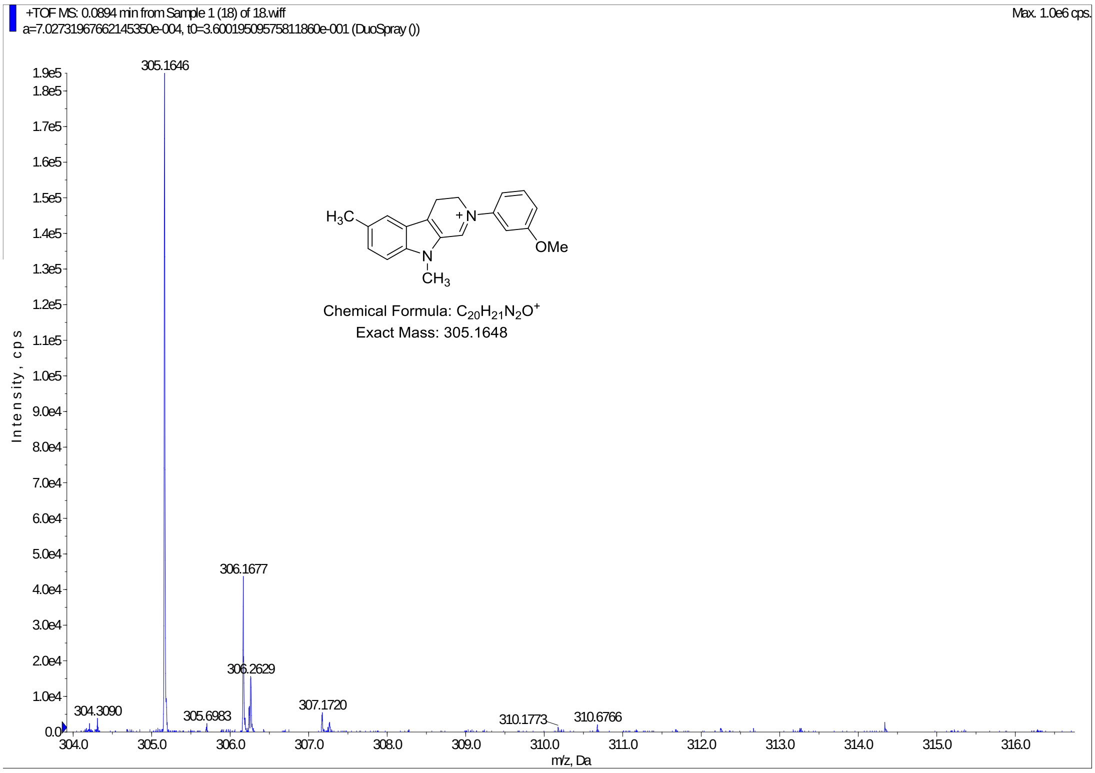

Positive ESI-HR-MS of Compound 6-18

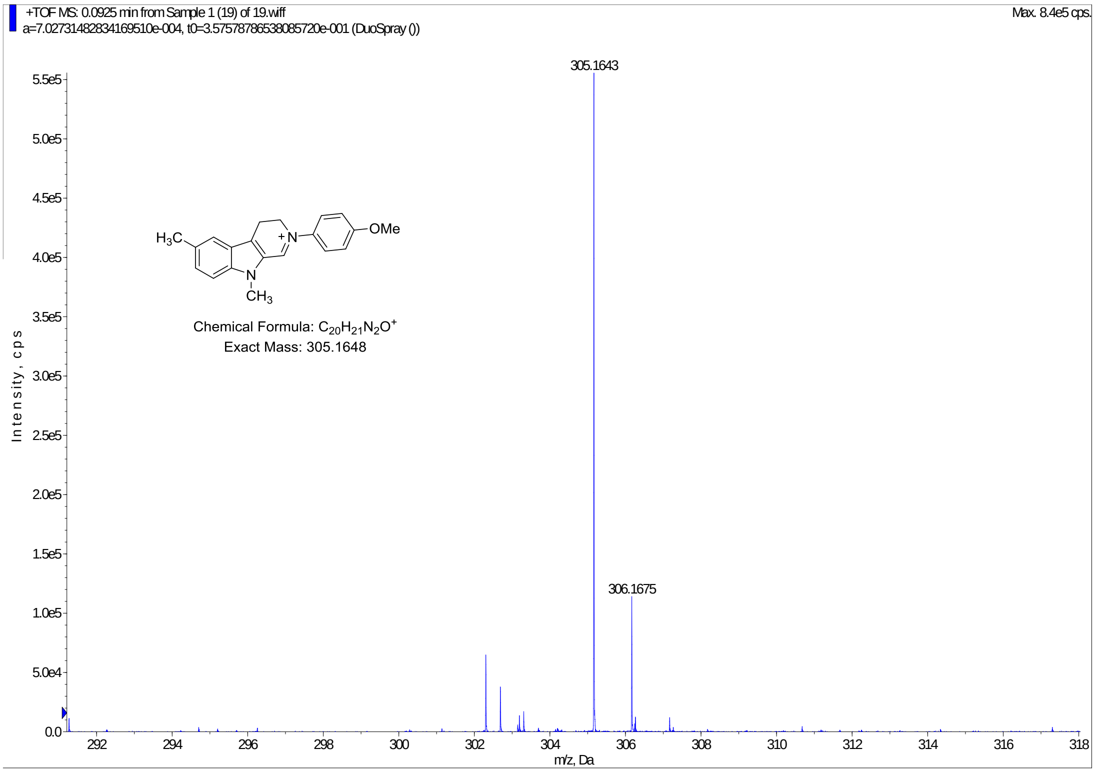

Positive ESI-HR-MS of Compound 6-19

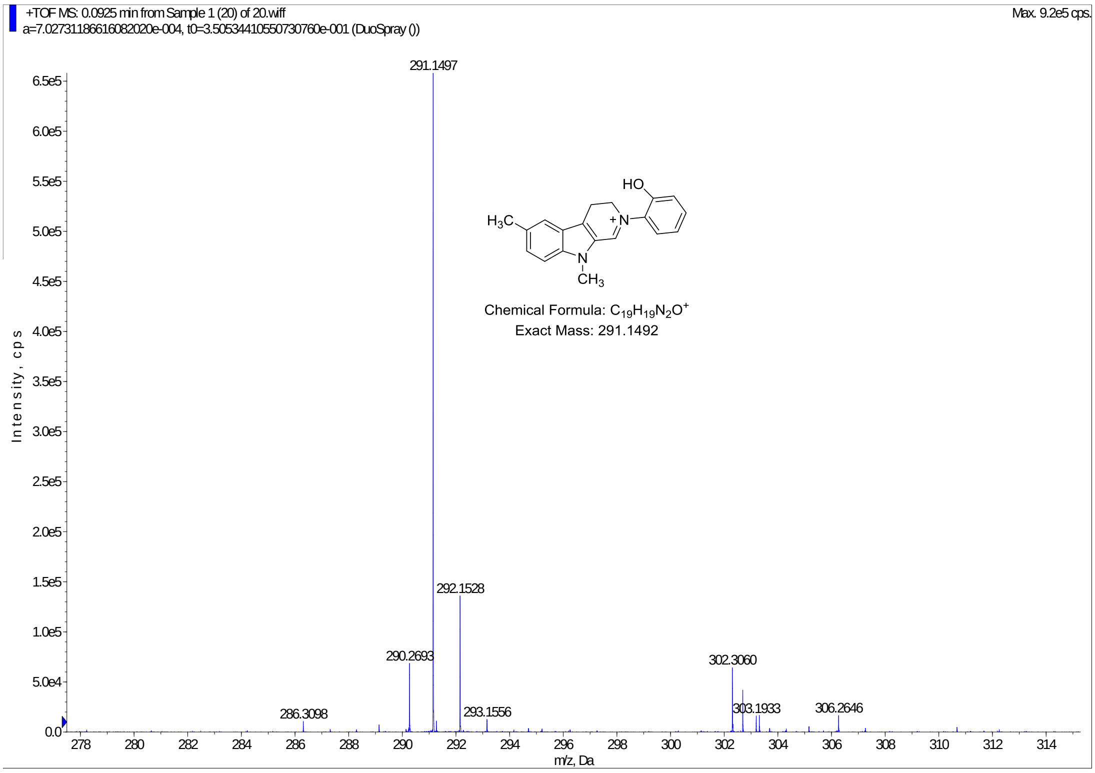

Positive ESI-HR-MS of Compound 6-20

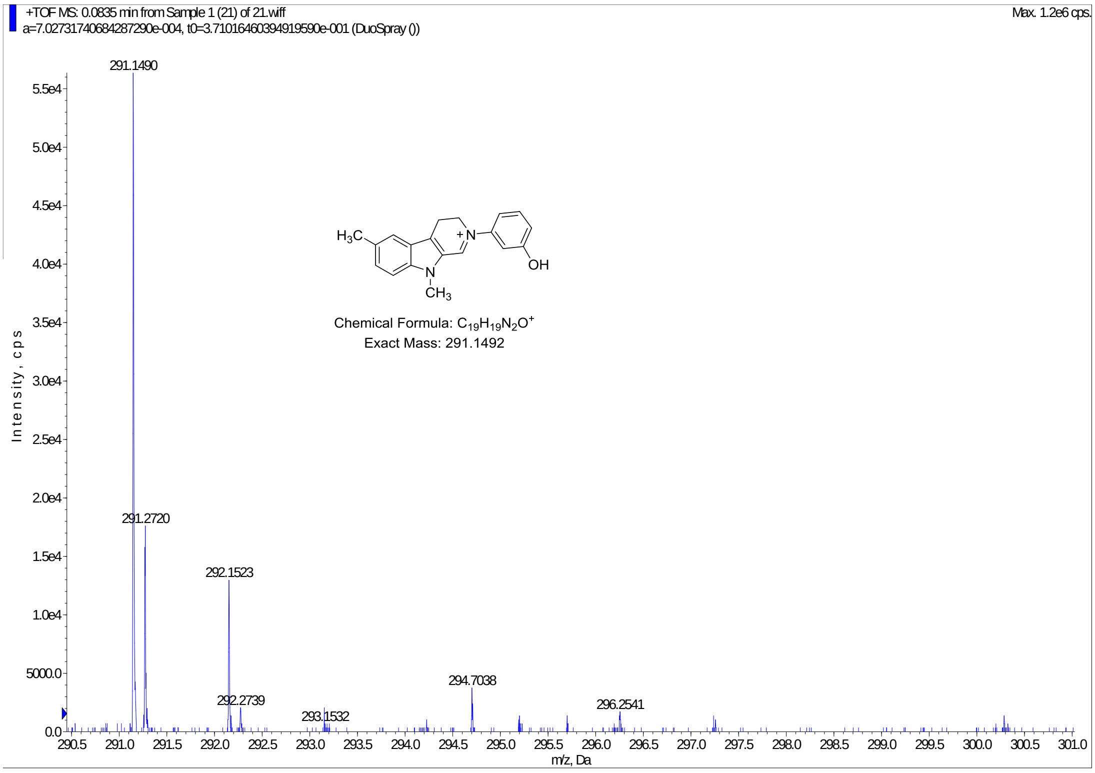

Positive ESI-HR-MS of Compound 6-21

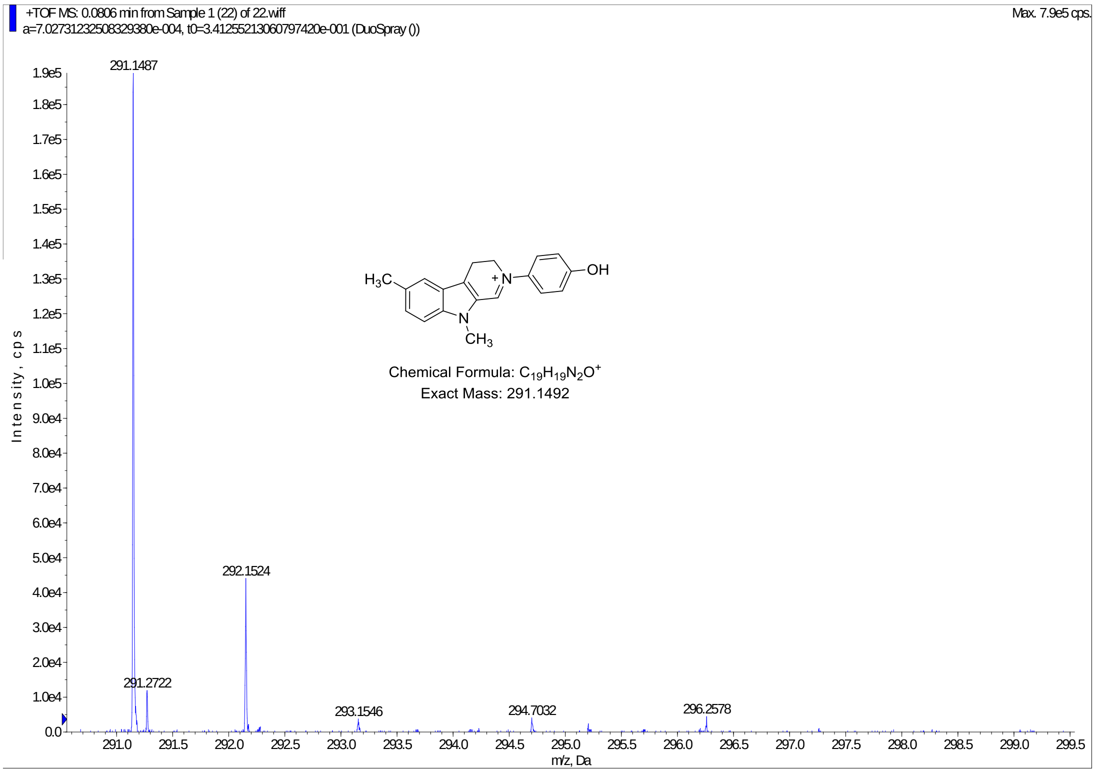

Positive ESI-HR-MS of Compound 6-22

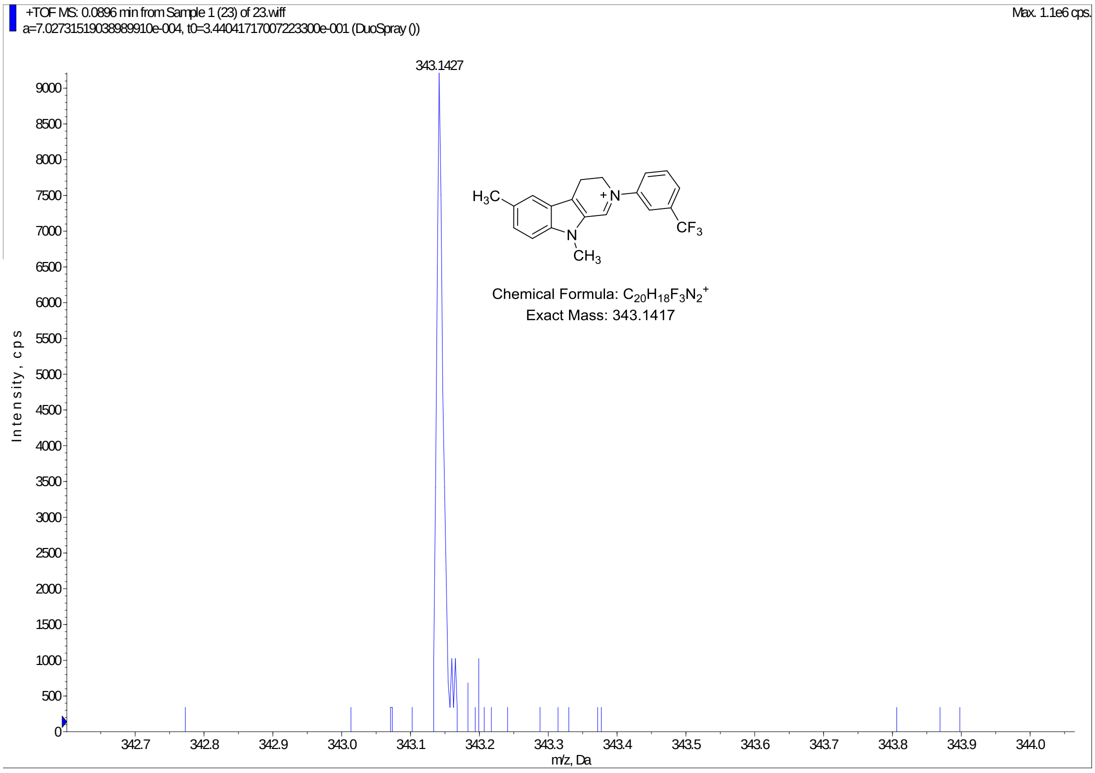

Positive ESI-HR-MS of Compound 6-23

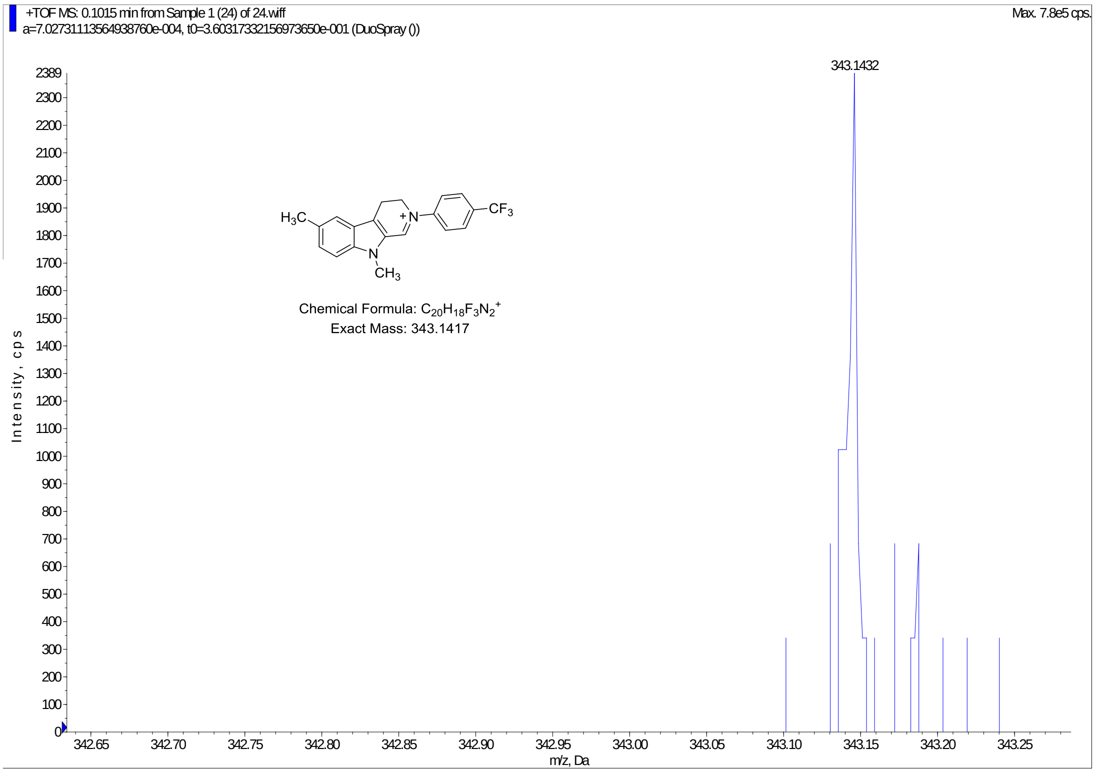

Positive ESI-HR-MS of Compound 6-24

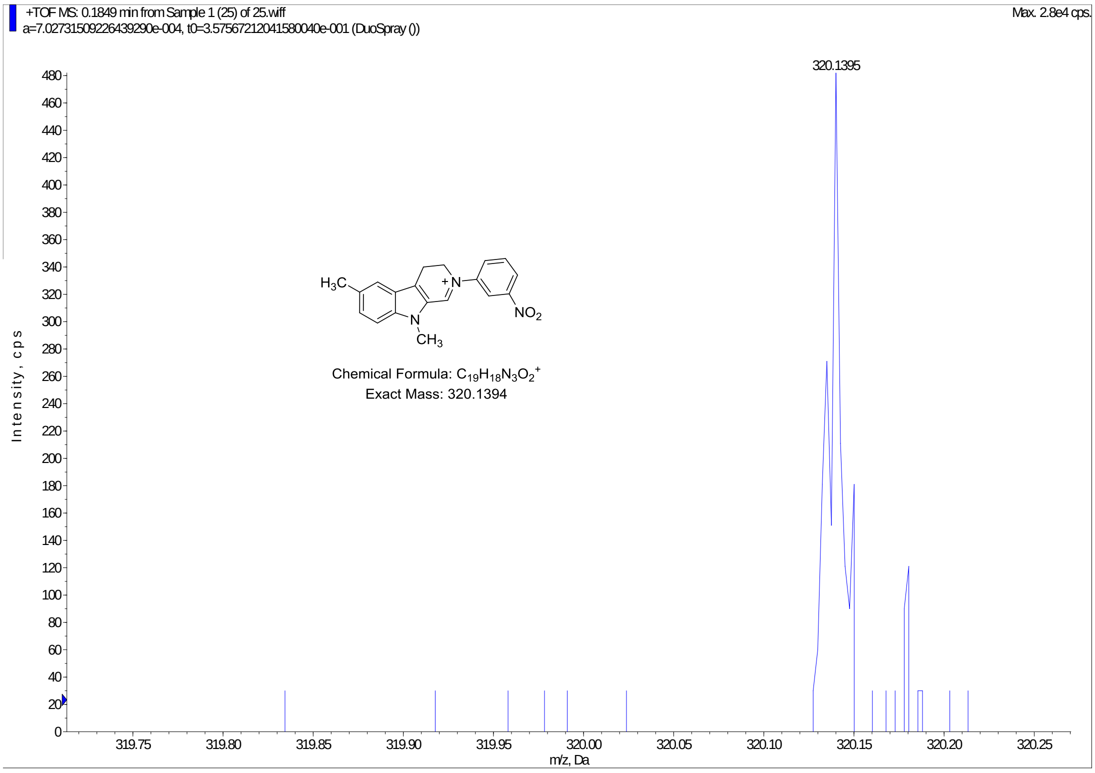

Positive ESI-HR-MS of Compound 6-25

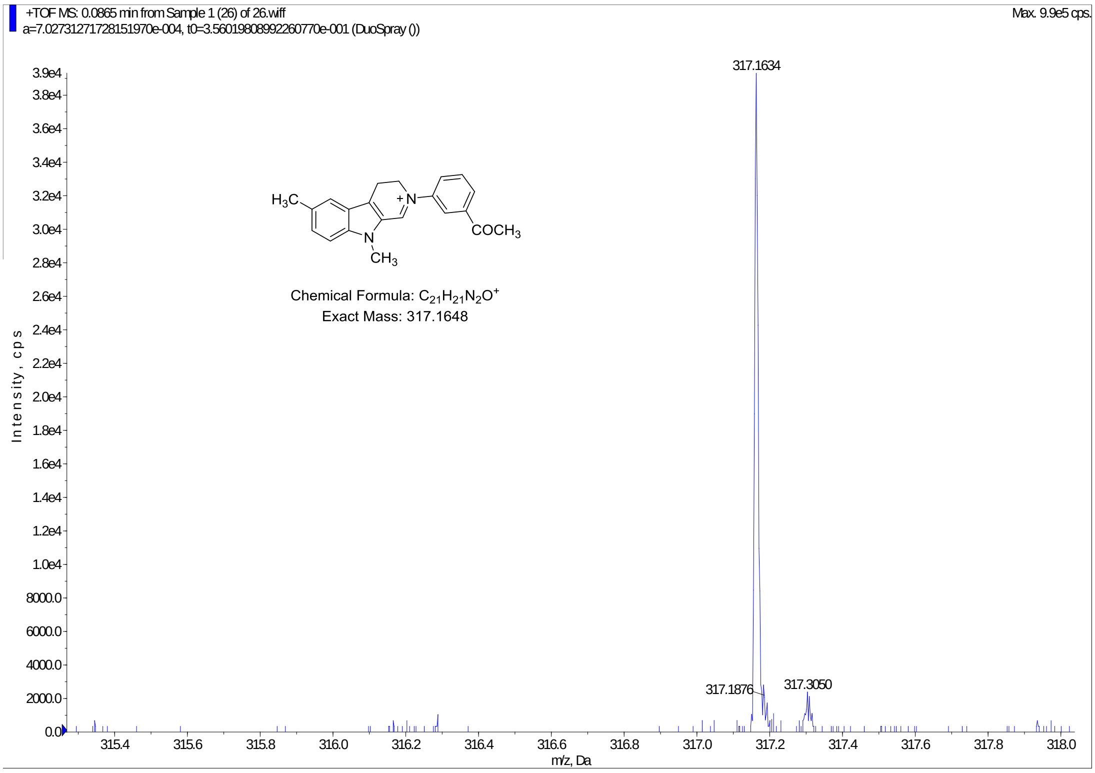

Positive ESI-HR-MS of Compound 6-26

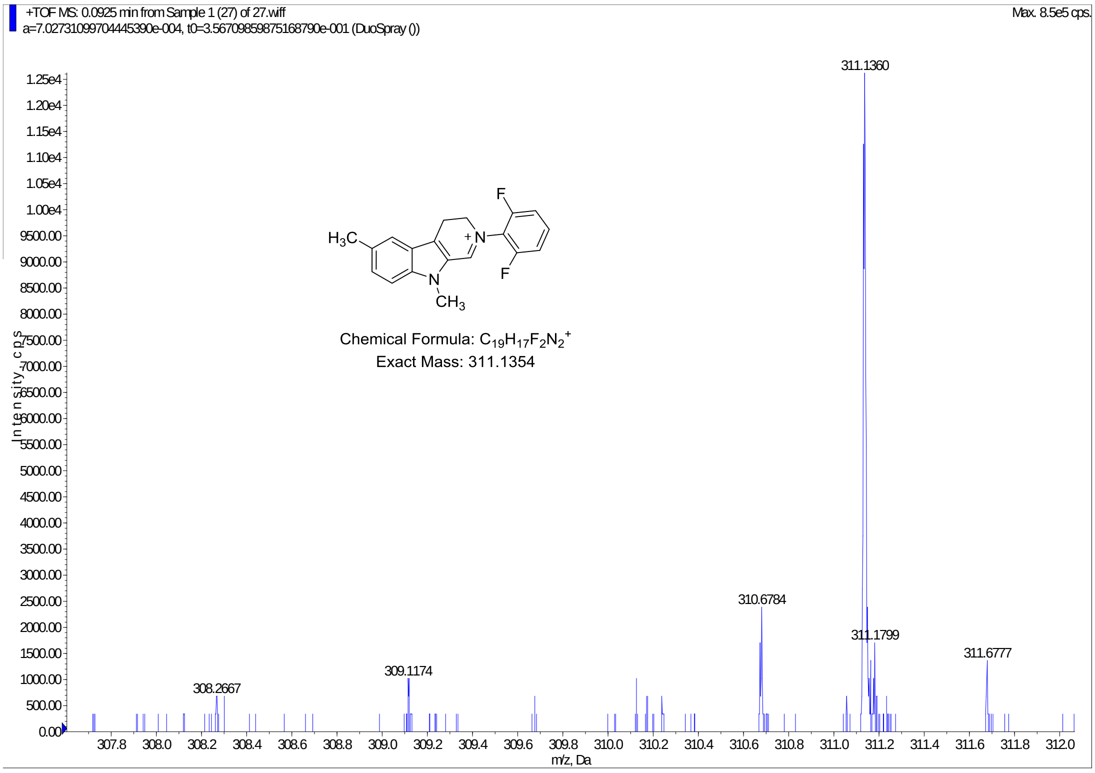

Positive ESI-HR-MS of Compound 6-27

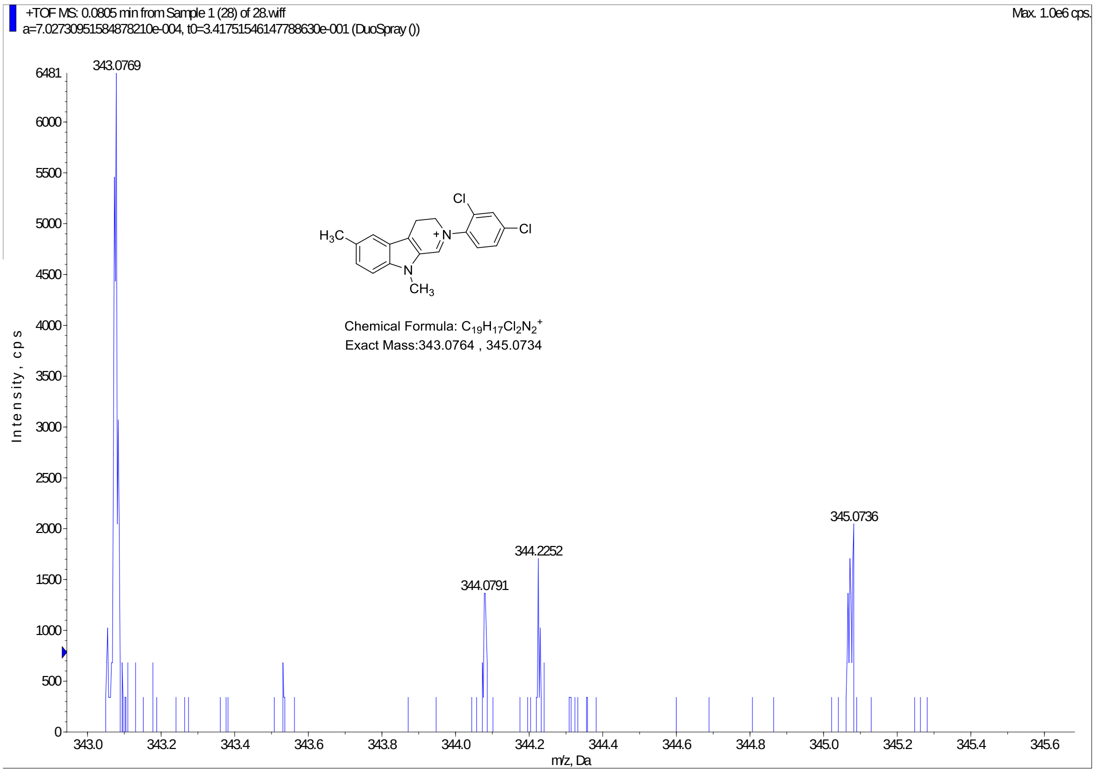

Positive ESI-HR-MS of Compound 6-28

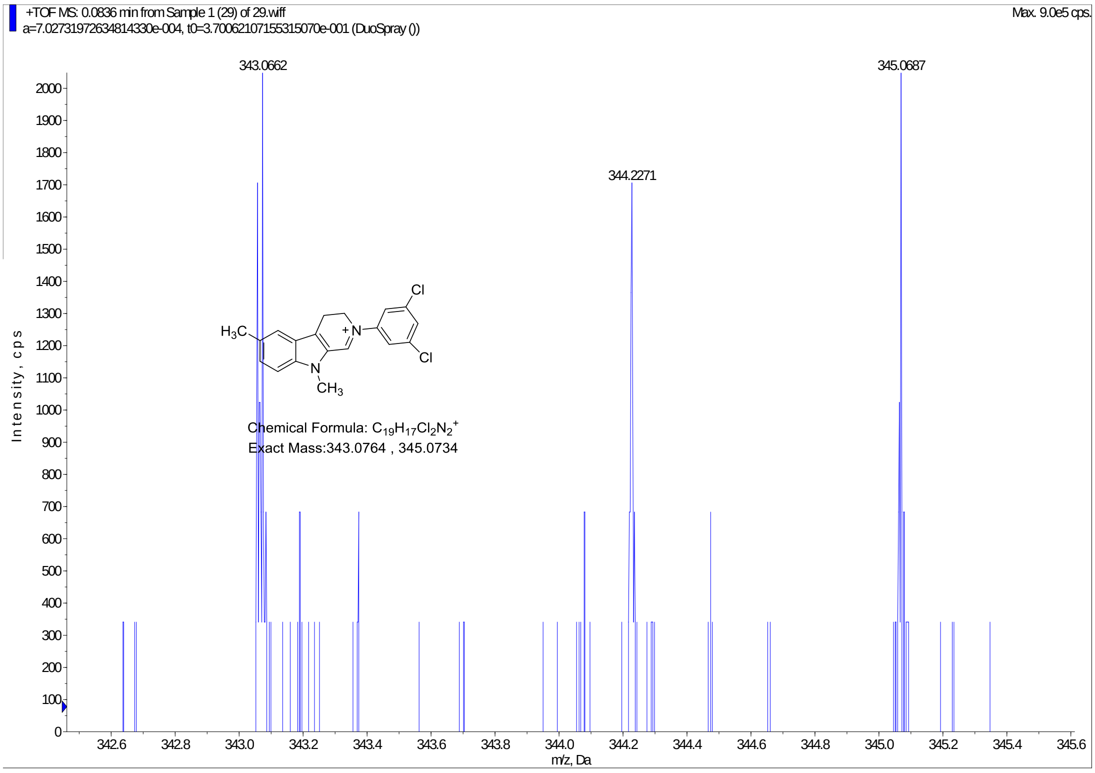

Positive ESI-HR-MS of Compound 6-29

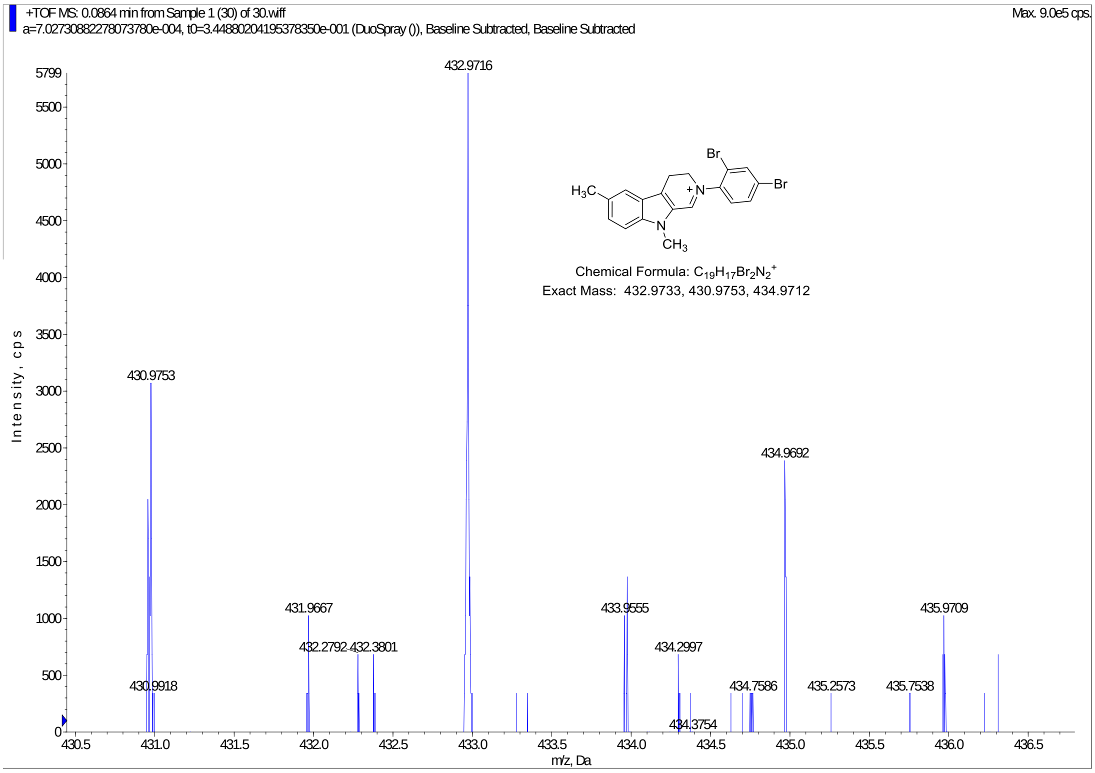

Positive ESI-HR-MS of Compound 6-30

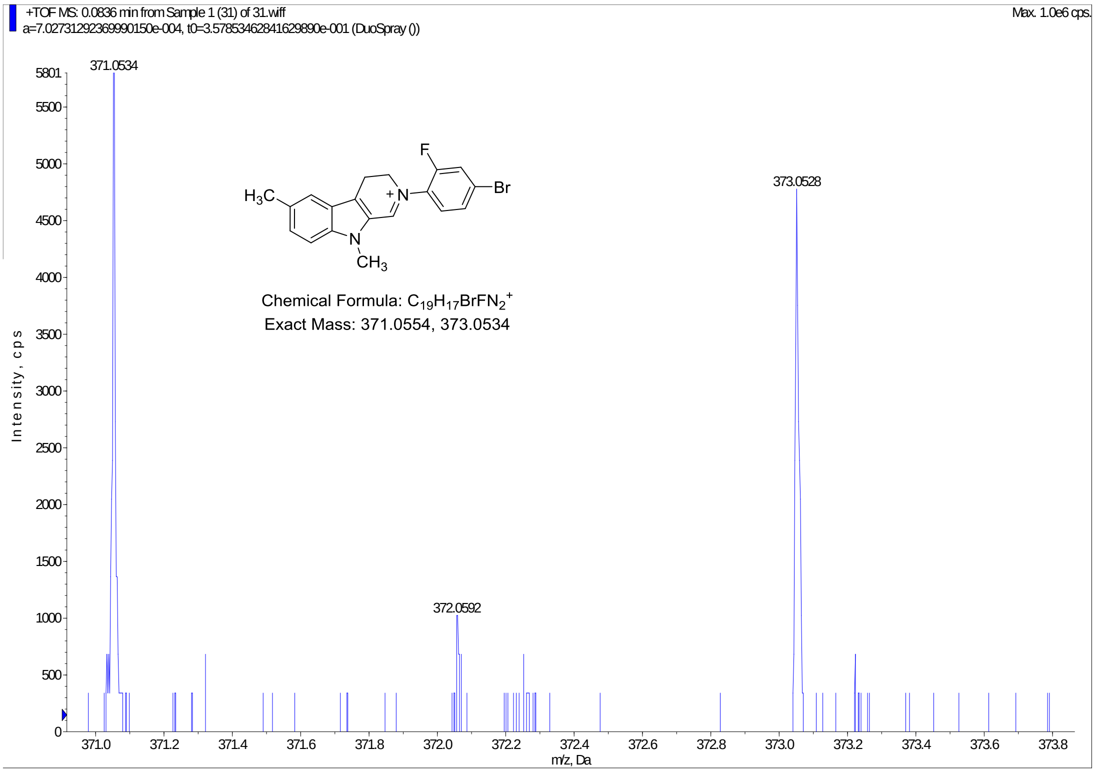

Positive ESI-HR-MS of Compound 6-31

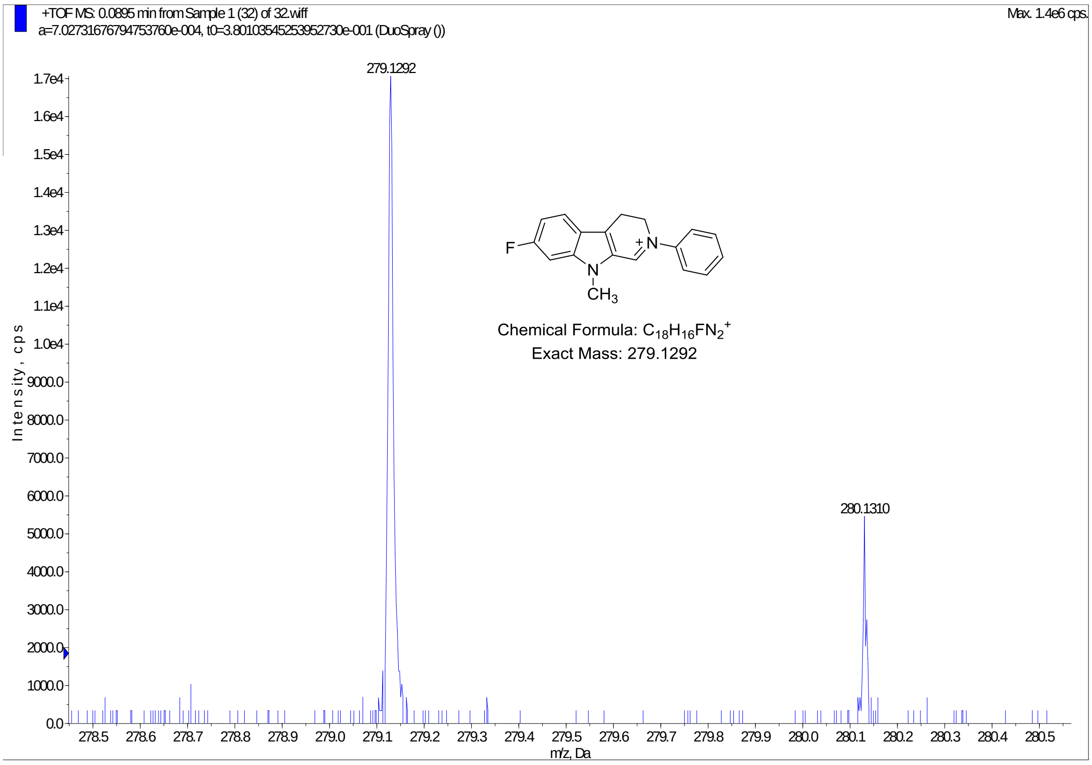

Positive ESI-HR-MS of Compound 6-32

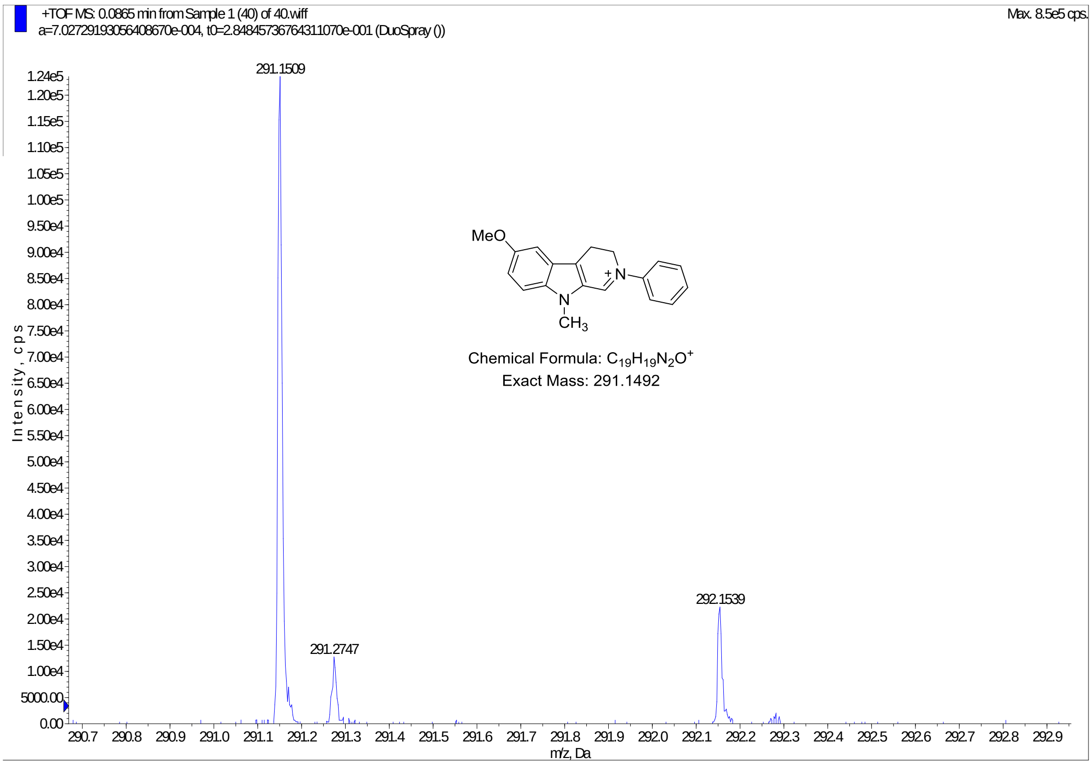

Positive ESI-HR-MS of Compound 6-33
